# Construction of high-resolution genetic maps of *Zoysia matrella* (L.) Merrill and applications to comparative genomic analysis and QTL mapping of resistance to fall armyworm

**DOI:** 10.1186/s12864-016-2969-7

**Published:** 2016-08-08

**Authors:** Xiaoen Huang, Fangfang Wang, Ratnesh Singh, James A. Reinert, M. C. Engelke, Anthony D. Genovesi, Ambika Chandra, Qingyi Yu

**Affiliations:** 1 Texas A&M AgriLife Research Center at Dallas, Texas A&M University System, Dallas, TX 75252 USA; 2 Department of Soil & Crop Sciences, Texas A&M University, College Station, TX 77843 USA; 3 Department of Plant Pathology & Microbiology, Texas A&M University, College Station, TX 77843 USA

**Keywords:** *Zoysia*, Chloridoideae, Genetic map, Resistance to fall armyworm, Restriction site-associated DNA sequencing (RADSeq)

## Abstract

**Background:**

*Zoysia matrella*, widely used in lawns and sports fields, is of great economic and ecological value. *Z. matrella* is an allotetraploid species (2*n* = 4*x* = 40) in the genus zoysia under the subfamily Chloridoideae. Despite its ecological impacts and economic importance, the subfamily Chloridoideae has received little attention in genomics studies. As a result, limited genetic and genomic information are available for this subfamily, which have impeded progress in understanding evolutionary history of grasses in this important lineage. The lack of a high-resolution genetic map has hampered efforts to improve zoysiagrass using molecular genetic tools.

**Results:**

We used restriction site-associated DNA sequencing (RADSeq) approach and a segregating population developed from the cross between *Z. matrella* cultivars ‘Diamond’ and ‘Cavalier’ to construct high-resolution genetic maps of *Z. matrella*. The genetic map of Diamond consists of 2,375 Single Nucleotide Polymorphism (SNP) markers mapped on 20 linkage groups (LGs) with a total length of 1754.48 cM and an average distance between adjacent markers at 0.74 cM. The genetic map of Cavalier contains 3,563 SNP markers on 20 LGs, covering 1824.92 cM, with an average distance between adjacent markers at 0.51 cM. A higher level of genome collinearity between *Z. matrella* and rice than that between *Z. matrella* and sorghum was revealed by comparative genomic analysis. Pairwise comparison revealed that two independent nested chromosome fusion events occurred after *Z. matrella* and sorghum split from a common ancestor. The high-resolution linkage maps were applied into mapping QTLs associated with fall armyworm (FAW) resistance and six loci located on LGs 8 and 20 were detected to be significantly associated with FAW resistance.

**Conclusion:**

The high-resolution linkage maps provide anchor points for comparative genomics analysis between *Z. matrella* and other grass species. Our comparative genomic analysis suggested that the chromosome number reduction from 12 to 10 had occurred independently via a single-step in the subfamilies Chloridoideae and Panicoideae. The high-resolution genetic maps provide an essential framework for mapping QTLs associated with economically and agronomically important traits. The major QTLs mapped on LG8 of the Cavalier map provide a starting point for cloning FAW resistance genes and further studies for a better understanding of FAW resistance in zoysiagrass.

**Electronic supplementary material:**

The online version of this article (doi:10.1186/s12864-016-2969-7) contains supplementary material, which is available to authorized users.

## Background

*Zoysia matrella* (L.) Merrill (2n = 4x = 40), commonly known as Manila Grass, is naturally distributed in South-East Asian countries, along the coasts of Indian Ocean, and in southern Japan (Ryukyu Islands) and northern Australia [1]. *Z. matrella* was first introduced into the United States from Manila in the early 20th century and since then it has been increasingly used as a turfgrass on athletic fields, golf courses, home lawns, and parks [2]. Its fine leaf texture, high density, rhizomatous growth habit, and excellent tolerance to shade, heat, and salinity stresses has made it an ideal choice for high quality playing surfaces. *Z. matrella* has become an economically important warm-season turfgrass, widely growing in the southern United States, Japan, China, Southeast Asia, New Guinea, Australia and New Zealand.

The primary focus of the zoysiagrass (*Zoysia* spp.) breeding program is to improve resistance or tolerance to biotic and abiotic stresses in order to reduce replacement and management costs incurred by the end user. The fall armyworm (FAW), *Spodoptera frugiperda* (J.E. Smith), is a destructive pest in over 60 plant species, including the major cool-season and warm-season turfgrasses, corn, wheat, rice, sugarcane, and sorghum [3]. Outbreaks of FAW are sporadic and unpredictable, making it very difficult to control. Utilization of host plant resistance is the most effective, efficient, and economical approach in pest control. In addition, the use of pest-resistant or tolerant varieties can help to reduce pesticide application and meet the increasing strict environmental quality and safety standards. For this reason, resistance to FAW was evaluated in major zoysiagrass cultivars [4–8]. Among the twelve cultivars or genotypes evaluated, ‘Cavalier’ (*Z. matrella*) exhibited the highest resistance to FAW, regardless of the development stage of the larvae [8]. No FAW larvae were observed to be able to survive more than 17 days on Cavalier [8]. While, cultivar ‘Diamond’ (*Z. matrella*) served as an excellent host and produced the largest larval weight in the test [8]. An F1 segregating population was created from a cross between the FAW resistant cultivar Cavalier and the sensitive cultivar Diamond. In the present study, we developed high-resolution linkage maps of *Z. matrella* and identified major quantitative trait loci (QTLs) associated with FAW resistance utilizing this segregating population.

Effort has been made to map FAW resistance in zoysiagrass by Jessup et al. [9]. However, a limited number of molecular markers were used to develop the linkage map of *Z. matrella*, which could lead to inaccurate genetic mapping of QTLs. The locus of FAW resistance was mapped on the linkage group (LG) 36 that only contains three markers [9], which significantly limited its application in marker-assisted breeding and cloning the genes controlling the resistance to FAW through a positional cloning approach.

The advent of next-generation sequencing (NGS) technologies provides us new opportunities to identify Single Nucleotide Polymorphism (SNP) with high resolution in a cost-effective manner at a much faster speed than ever before. Restriction site-associated DNA sequencing (RADSeq), a method that utilizes NGS and restriction enzyme digestion to reduce the complexity across the targeted genomes enabling discovery of genome-wide genetic markers [10, 11], has become a powerful tool for a wide range of genetic studies in both model and non-model organisms. RADSeq has been applied in genetic map construction as well as detection of QTLs that impact economically and agronomically important traits for a number of plant species, including eggplant [12], barley [13], ryegrass [14], grape [15], lotus [16], pear [17], and *Z. japonica* [18]. In the present work, we developed high-resolution linkage maps of *Z. matrella* using RADSeq markers to serve as foundation for gene mapping, QTL studies, assigning genome sequence to chromosomes, and comparative genomics studies.

The true grasses, family Poaceae (also known as Gramineae), provide for the major source of carbohydrates and proteins need by humans as well as various herbivores and fowl. Panicoideae and Chloridoideae are the two major subfamilies in the family Poaceae, which include mostly C4 grasses that are adapted to warm climates. *Z. matrella* is a member of the subfamily Chloridoideae which has attracted less attention in genomics research due to its relatively smaller economic impact compared to its sister subfamily Panicoideae. The sequence-tagged high-resolution linkage map of *Z. matrella* provides a framework for comparative and evolutionary genomic studies thus allowing us to further investigate the evolutionary history of this underexplored grass family.

## Methods

### Plant materials and DNA isolation

The F_1_ segregation population of 95 individuals created from a cross between *Z. matrella* varieties Cavalier (resistant to FAW) and Diamond (susceptible to FAW) was used for construction of the high-resolution linkage maps of *Z. matrella*. The mapping population was planted in pots and maintained in a greenhouse at Texas A&M AgriLife Research Center at Dallas, Texas, USA.

Young and healthy leaf tissue was harvested from each individual of the mapping population and frozen in liquid nitrogen immediately. The leaf tissue was then stored in a −80 °C freezer until DNA isolation. Approximately 100 mg of leaf tissue of each sample was ground into fine powder using liquid nitrogen, and then used for genomic DNA isolation with DNeasy Plant Mini Kit (Qiagen, Valencia, CA). The extracted DNA samples were run on an agarose gel and measured with a NanoDrop 2000c (Thermo Fisher Scientific, Waltham, MA) to ensure the DNA quality for RADSeq library construction. The high-quality DNA was then quantified by a Qubit fluorometer with the Qubit double-stranded DNA HS Assay kit (Life Technologies, Carlsbad, CA).

### RADSeq library construction and sequencing

The RADSeq libraries were constructed using a modified protocol described by Wang et al. [18]. Approximately 1 μg of genomic DNA was digested with restriction enzymes *Nsi*I (New England Biolabs, Ipswich, MA) and *Mse*I (New England Biolabs, Ipswich, MA). The digested genomic DNA was ligated with P1 and P2 adapters containing unique identifying sequences (molecular identifier, MID, see Additional file 1: Figure S1). The ligation reaction was then deactivated at 65 °C for 20 min and purified with Axyprep Mag PCR clean-up beads (Axygen, Union City, CA) following the manufacturer’s instruction. Purified adaptor-ligated DNA was PCR amplified with12 cycles using Phusion High-Fidelity DNA polymerase (New England Biolabs, Ipswich, MA). Then, size selection of 300–500 bp DNA fragments were performed using Axyprep Mag PCR clean-up beads (Axygen, Union City, CA) following the manufacturer’s instruction. Quantification of recovered DNA was performed on a Qubit fluorometer (Life Technologies, Carlsbad, CA). The RADSeq libraries of the whole mapping population were pooled and sequenced on an Illumina HiSeq 2000 (Illumina, San Diego, CA).

### RADSeq sequence analysis and SNP calling

Raw data processing and SNP identification were performed using modules implemented in the open-source STACKS pipeline v1.19 [19]. Raw reads were de-multiplexed and trimmed to remove low-quality sequences and adapter contamination using the ‘PROCESS_RADTAGS’ module of the Stacks package. Sequences with base call accuracy lower than 99 % (Q20) in a sliding window of 15 % of the read-length were considered to be low quality and trimmed. Filtered reads were then subjected to build stacks using ‘USTACKS” module. A set of *de novo* loci were defined with no more than two nucleotide mismatches and at least six reads in each stack. A catalog of parental loci was created using ‘CSTACKS’ module. Progeny loci were compared to the parental loci in catalog and SNPs were called using maximum likelihood method implemented in ‘SSTACKS’ module. Finally, the haplotypes were determined and genotype data were exported as JoinMap compatible format using the ‘GENOTYPES’ module of STACKS pipeline.

### Construction of genetic maps

Linkage maps were constructed using JoinMap 4.0 [20] and outcross pollinated family (CP) was selected as the population type. Since both parents, Diamond and Cavalier, are tetraploids, we selected single-dose markers for linkage analysis to avoid statistical challenges caused by complicated segregation patterns for the reason as described by Wu et al. [21]. Markers that are heterozygous in Diamond and homozygous Cavalier (‘lm x ll’ type) were selected to build Diamond linkage groups. Markers that are heterozygous in Cavalier and homozygous in Diamond (‘nn x np’ type) were selected to build Cavalier linkage groups. QC-filtered SNPs were further filtered by the following standards: 1) markers must be genotyped in at least 80 out of 95 individuals; 2) Individuals with over 10 % missing data were discarded; 3) Markers segregating at distorted Mendelian ratio (expected ratio for ‘lm x ll’ type and ‘nn x np’ type is 1: 1, χ^2^ test, *P* < 0.05) were discarded; 4) Redundant markers were removed by standard of similarity = 1.

The linkage groups were built using regression mapping algorithm, with a minimum logarithm of odds (LOD) value at 7, and a maximum recombination frequency at 0.35. Marker positioning calculation was performed with a goodness-of-fit jump at 5, followed by a “ripple” procedure (value =1). Kosambi mapping function was used to correct linkage distance. ‘N.N. fit’ function of JoinMap 4.0 was used to check the map quality. MAPCHART 2.2 [22] was used to draw linkage maps.

### Comparison with other grass genomes

We used BLASTN (BLAST 2.2.28+) [23] with default parameter settings and an e-value cutoff of 1 × 10^−8^ to blast the consensus sequences of mapped RADSeq markers against the genome sequences and gene models of rice and sorghum genomes. Markers that showed significant hits to the genome sequences and/or gene models of rice and sorghum genomes were extracted and used for comparative genomics study.

### In vitro insect feeding bioassays

The whole mapping population including the parents were subjected to in vitro insect feeding bioassays using the method described by Reinert and Engelke [8]. The bioassay was a no-choice experiment and conducted in the laboratory with 9-cm diam. x 20-mm deep plastic petri dishes as larvae feeding chambers. Each dish was provided with 3 g of fresh leaf tissue on two layers of water-saturated filter paper. Water was added daily as needed to maintain filter paper saturation and grass-clipping turgor. Grass clippings were added daily as needed during the whole experiment to ensure turgid fresh grass was always available to the developing larvae. The FAW used in the bioassay was a corn strain requested from the USDA-ARS-IBPMRL at Tifton, GA. Each individual of the mapping population was phenotyped using 4-day-old larvae that had first fed on fresh tissue of a susceptible zoysiagrass genotype, DALZ 8516. DALZ 8516 is an excellent host of FAW with near 100 % survival. Three 4-day-old larvae were randomly selected and placed into the feeding chambers with clippings from the respective individuals making up the mapping population, in a randomized complete block design (RCB) replicated 8 times. Each replicate started with 3 larvae for the first 3–4 days. And then the 3 larvae were separated into separate dishes. Larvae survivorship was measured at 7-, 10-, and 17-day-old larvae. The fresh weight was measured at 12-day-old larvae. The number of days to pupation and to adult emergence, and the sex types of larvae were also recorded. The bioassay for the whole population was split into 8 experiments. The two parents and a susceptible zoysiagrass genotype, DALZ 8516, were included in each experiment and used as controls to normalize the data between experiments. Percentage of mortality was adjusted by Abbott’s Formula [24].

### QTL analysis

Phenotypic data which includes mortality rate of the 7-, 10-, and 17-day-old FAW larvae for each individual and the genetic map of Cavalier were used for QTL analysis with MapQTL 5 [25]. The non-parametric Kruskal–Wallis (KW) *K*-test was performed for detection of the presence and locations of QTLs. QTL significance in the Kruskall-Wallis analysis was based on *P* value < 0.001 significance level as suggested by the software.

## Results

### RAD sequencing and SNP discovery

We genotyped all 95 individuals and parents that make up the whole mapping population using RADSeq. A total of 270,460,752 raw sequence reads were obtained (Additional file 2: Table S1, Additional file 1: Figure S1). After filtering low-quality sequence reads, 267,772,333 high-quality reads remained. All the RADSeq sequences can be accessed through the NCBI SRA database under BioProject Accession PRJNA312939 (http://www.ncbi.nlm.nih.gov/bioproject/312939). To maximize the chances of detecting segregating SNPs in the parents, we RADSeq’ed the parents more extensively than the offspring. We obtained 10,652,144 high-quality reads for the parent Diamond and 13,307,302 high-quality reads for Cavalier. The number of sequence reads of the F1 progeny ranged from 0.91 to 4.32 million reads. The average sequence depth for the F1 progeny was 2,566,451 reads per progeny (Additional file 2: Table S1, Additional file 1: Figure S1).

Using ‘STACKS’ software, we identified 11,359 SNP markers. To avoid statistical challenges caused by the complex segregation patterns of polyploid genomes, only single-dose alleles (SDAs) were kept for linkage analysis. Markers having a pattern of heterozygous in Diamond and homozygous in Cavalier were selected to build Diamond linkage groups. Markers that are heterozygous in Cavalier and homozygous in Diamond were selected to construct Cavalier linkage groups. Among the 11,359 SNPs, 8,485 of them were identified as SDAs. A total of 3,323 SNPs proved to be heterozygous in Diamond and homozygous in Cavalier, and a total of 5,162 SNPs were found to be heterozygous in Cavalier and homozygous in Diamond. We further filtered F1 individuals that contained a large number of missing data and SNP markers that showed non-Mendelian and skewed segregation patterns (expected ratio for ‘lm x ll’ type and ‘nn x np’ type is 1: 1, χ^2^ test, *P* < 0.05). Among the 95 F1 individuals, 25 individuals showed more than 10 % missing data or markers with extreme distorted segregation patterns. The remaining 70 individuals were used for linkage map construction. Among the 3,323 Diamond markers, 2,462 markers remained after filtering identical markers and removing markers with more than 10 % missing data and significantly distorted markers. Among the 5,162 Cavalier SNP markers, 3,793 markers remained after filtering identical markers and removing markers with more than 10 % missing data and significantly distorted markers. The detailed filtering process is shown in Table 1. The final dataset of 2,462 and 3,793 SNP markers were used to construct genetic maps for Diamond and Cavalier, respectively.

**Table 1:** Summary of SNP marker filtering procedure

Filtering Step	Number of SNPs Remaining	
Raw RADSeq processing	11,359	
Number of single-dose alleles	8,485	
Type of single-dose alleles	Diamond	Cavalier
Number of single-dose alleles before filtering	3,323	5,162
After removal of identical markers	3,311	5,136
After removal of markers with more than 10 % missing data	2,858	4,307
After removal of significantly segregation distorted markers (*P* < 0.05)	2,462	3,793

### Linkage map construction

A pseudo-testcross strategy was used to develop the genetic maps of *Z. matrella*. Because only single-dose markers were selected for genetic map construction, the segregation ratio of the selected markers should be 1:1 in the F1 progeny. The selected markers were then divided into two data sets based on their segregation patterns. Each data set contains the meiotic segregation information of one parent. Then the two segregation data sets were subjected to construction of two independent linkage maps of *Z. matrella*, one for each parent.

Using JoinMap 4 [20], the selected markers were grouped into distinct LGs. Among the 2,462 single-dose SNP markers selected for linkage analysis in Diamond, 2,455 (99.7 % of the input markers) of them were grouped into 20 major LGs with a LOD score ≥7, 1 marker remained as singleton, and 6 markers were assigned to 2 small LGs with 4 and 2 markers for each LG, respectively. Among the 3,793 SNPs selected for linkage analysis in Cavalier, 3,784 (99.8 % of the input markers) of them were grouped into 20 major LGs with a LOD score ≥7, 2 markers remained unlinked, and 7 markers were assigned to 2 small LGs with 4 and 3 markers for each LG, respectively.

The markers in each LG were positioned and ordered using regression mapping algorithm (see parameters in materials and methods). Markers of “suspect linkages” were inspected for accuracy in scoring and removed from the map calculation if they proved problematic or troublesome. A total of 148 markers from Diamond LGs and 221 markers from Cavalier LGs were identified as “suspect linkages” during mapping and thus were discarded from subsequent linkage analysis.

**Fig. 1:**
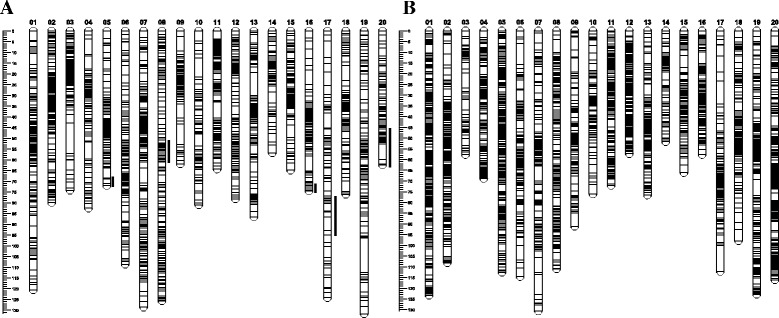
The distribution of RADSeq markers on the high-resolution linkage maps of *Zoysia matrella*. **a** Genetic map of Diamond. The number on the top of each linkage group (LG) shows LG number and the ruler on the left of each genetic map indicates the length of the LG in centiMorgans (cM). The black lines on the right of Diamond LGs indicates segregation distortion regions (SDRs). **b** Genetic map of Cavalier

Compared to the Cavalier linkage map, Diamond LGs contained fewer numbers of markers. We attempted to place the distorted markers back to increase the marker-density of the Diamond linkage map. The distorted markers were kept on the final linkage map only when their presence did not change the orders of surrounding markers. A total of 68 distorted markers, accounting for 2.9 % of the total mapped markers, were placed back on the Diamond linkage map. The distorted markers were not evenly distributed across LGs. The majority of the distorted markers were clustered together and formed segregation distortion regions (SDRs). Five SDRs were detected on LGs5, 8, 16, 17, and 20 and three of these SDRs were located near the ends of the LGs (Fig. 1a). Four distorted markers on LG5 were clustered at one end of the LG, extending the length of LG5 from 63.8 cM to 72 cM. Thirty-eight distorted markers formed the largest cluster in the middle section of LG8 (50.7 cM - 62.2 cM), increasing the length of LG8 from 91.7 cM to 125.6 cM. The final LG16 contained 2 distorted markers at one end of the LG, extending the length of LG16 from 71 cM to 74.5 cM. LG17 (total length 124.2 cM) contained 5 distorted markers between 78.9 cM and 95 cM without changing the length of the LG. LG20 contained 19 distorted markers at one end of the LG, extending the length of LG20 from 49.3 cM to 62.4 cM.

We numbered each LG based on their homology with sorghum chromosomes (see details in ‘comparative genomics analysis’). For each sorghum chromosome, we identified two homologous LGs, eg. both LG1 and LG2 are homologs of sorghum chromsome1 (Sb01) and LG3 and LG4 are homologs of Sb02, etc. (Table 2). The total lengths of the final genetic maps for Diamond and Cavalier were 1,754.48 cM and 1,824.92 cM, respectively. Considering that Diamond and Cavalier share almost the same genome size, the average recombination ratio between the two was estimated at 1: 1.04 and no significant difference for overall recombination rates was observed between the two genomes. The mapped markers are distributed unevenly along each LG. In general, the middle sections of LGs contained the highest density of markers, while, both ends of each LG had the lowest density of markers. The ‘Gap <5 cM’ value, which reflects linkage degree between adjacent markers, was 98 and 99 % for Diamond and Cavalier genetic maps, respectively.

**Table 2:** Summary statistics for Diamond and Cavalier linkage maps

Sorghum Chr. no.	Diamond inkage Groups							Cavalier Linkage Groups						
	LG	Size (cM)	No. of markers	Density (cM)	Gaps <5 cM (%)	Max. gap size (cM)	No. of distorted markers	LG	Size (cM)	No. of markers	Density (cM)	Gaps <5 cM (%)	Max. gap size (cM)	No. of distorted markers
Chr01	01	120.48	151	0.80	98 %	5.3	0	01	123.34	351	0.35	100 %	4.0	0
	02	79.86	207	0.39	100 %	3.7	0	02	108.09	277	0.39	99 %	5.1	0
Chr02	03	74.24	117	0.63	99 %	14.2	0	03	57.66	53	1.09	98 %	7.0	0
	04	82.51	88	0.94	97 %	7.6	0	04	68.93	193	0.36	100 %	3.8	0
Chr03	05	71.99	99	0.73	100 %	4.6	4	05	112.63	325	0.35	100 %	3.3	0
	06	108.60	128	0.85	100 %	4.0	0	06	114.60	152	0.75	98 %	9.9	0
Chr04	07	128.61	226	0.57	99 %	6.9	0	07	130.62	195	0.67	98 %	9.2	0
	08	125.59	171	0.73	100 %	2.8	38	08	111.04	157	0.71	99 %	6.0	0
Chr05	09	61.85	81	0.76	96 %	9.6	0	09	91.20	102	0.89	97 %	6.4	0
	10	80.99	72	1.12	97 %	8.9	0	10	76.10	92	0.83	100 %	4.5	0
Chr06	11	64.34	156	0.41	99 %	7.3	0	11	72.10	196	0.37	100 %	3.6	0
	12	78.19	118	0.66	100 %	4.1	0	12	57.34	183	0.31	100 %	2.3	0
Chr07	13	86.58	141	0.61	99 %	10.7	0	13	76.62	152	0.50	99 %	5.0	0
	14	56.80	73	0.78	99 %	5.2	0	14	51.73	73	0.71	100 %	3.8	0
Chr08	15	64.82	108	0.60	99 %	5.8	0	15	66.05	118	0.56	99 %	5.7	0
	16	74.54	97	0.77	98 %	7.0	2	16	57.75	162	0.36	99 %	5.4	0
Chr09	17	124.24	67	1.85	92 %	7.8	5	17	112.21	119	0.94	97 %	12.2	0
	18	76.11	95	0.80	99 %	6.9	0	18	97.90	144	0.68	99 %	5.2	0
Chr10	19	131.72	110	1.20	99 %	15.9	0	19	122.92	242	0.51	99 %	7.3	0
	20	62.43	70	0.89	98 %	7.3	19	20	116.11	277	0.42	100 %	4.6	0
Total	20	1754.48	2375	0.74	98 %		68	20	1824.92	3563	0.51	99 %		0

*LG* Linkage group, Density: average distance between adjacent markers; Gaps <5 cM: Percentage of locus intervals where the distance between adjacent loci was smaller than 5 cM

The genetic map of Diamond consisted of 2,375 SNP markers distributed on 20 LGs, with an average interval of 0.74 cM (Fig. 1, Table 2). In the Diamond genetic map, the length of individual LG ranged from 56.8 cM (LG14) to 131.7 cM (LG19), and the number of markers mapped on each LG ranged from 67 (LG17) to 226 (LG7). LG2 showed the highest density of markers at 0.39 cM between adjacent markers, almost twice of the density at genome level. While, LG17 had the lowest density of markers at 1.85 cM between adjacent markers, 150 % reduction of the marker density compared with genome-wide average. No major interruption was observed on most LGs except three relatively large gaps (>10 cM), a 14.2 cM gap on LG3, a 10.7 cM gap on LG13, and a 15.9 cM gap on LG19. The detailed information of Diamond genetic map are given in Additional file 3: Figure S2 and Additional file 4: Table S2.

The final genetic map of Cavalier consisted of 3,563 SNP markers mapped on 20 major LGs. Compared with the Diamond linkage map, the Cavalier map had a much higher density with an average interval of 0.51 cM (Fig. 1, Table 2). The number of markers mapped on each LG varied from 53 (LG3) to 351 (LG1). The average interval of individual LG ranged from 0.31 cM (LG12) to 1.09 cM (LG3), and the length of individual LG varied from 51.73 cM (LG14) to 130.62 cM (LG7). The longest LG was LG7, which contains 195 loci spanning 130.62 cM. The shortest was LG14, which contains 73 loci spanning 51.73 cM. Owing to the high density, only 1 gap >10 cM, a 12.2 cM gap on LG17, was found in Cavalier linkage map. The detailed information of Cavalier genetic map are given in Additional file 5: Figure S3 and Additional file 6: Table S3.

### Conserved synteny relationships between *Z. matrella* and other grass genomes

Genetically mapped high-density RAD tags provide anchor points for comparative genomics analysis between *Z. matrella* and other grass species. One of the most important research topics in comparative and evolutionary genomics studies is karyotype evolution. Rice represents the most extensively studied and complete annotated genome among grasses. And it was suggested that the two major clades of grasses, Panicoideae-Aristidoideae-Centhothecoideae-Chloridoideae- Micrairoideae-Arundinoideae-Danthoideae (PACCMAD) and Bambusoideae-Ehrhartoidea-Pooideae (BEP), evolved from a common paleo-ancestor genome having a base chromosome number of *n* = 12 [26–30]. Comparative genomic analysis revealed the rice genome resembled the most of the ancestral form of the paleo-ancestor genome [26–30]. To address the question how *Z. matrella* genome obtained its reduced chromosome count from 12 to 10, we conducted comparative genomics analysis between *Z. matrella* and rice and identified the synteny relationships between these two genomes.

**Fig. 2:**
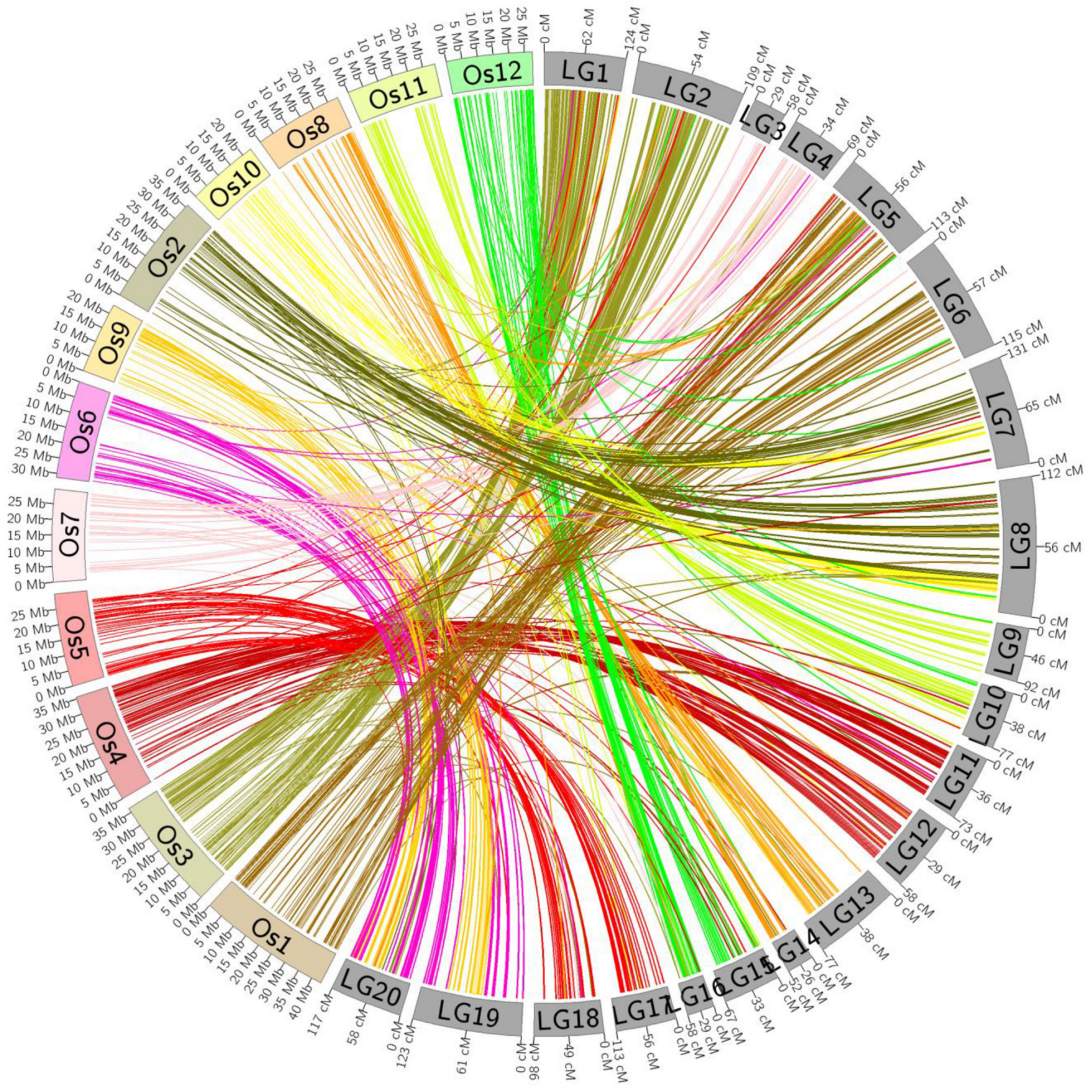
Genome comparison between *Zoysia matrella* and rice. Circos plot revealed the rice chromosome Os 10 inserted into the centromere region of Os 2 to form Cavalier LGs 7 and 8, and the rice chromosome Os 9 inserted into the centromere region of Os 6 to form Cavalier LGs 19 and 20

**Fig. 3:**
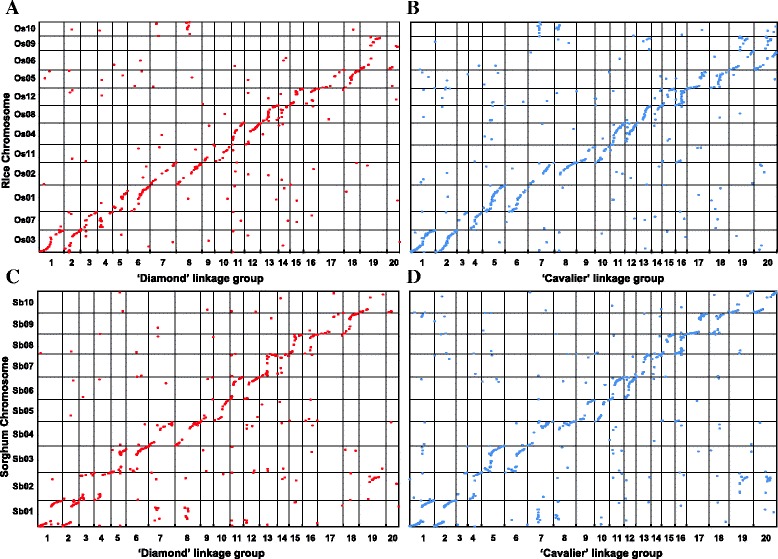
Genome comparison between *Zoysia matrella* and rice, and between *Z. matrella* and sorghum. **a** Comparison between Diamond linkage groups and rice chromosomes. The x-axis indicates the genetic location of markers on Diamond linkage groups in centiMorgans (cM); the y-axis shows the physical position of markers on rice chromosomes in megabases. Each dot corresponds to a RADSeq marker. **b** Comparison between Cavalier linkage groups and rice chromosomes. **c** Comparison between Diamond linkage groups and sorghum chromosomes. The x-axis indicates the genetic location of RADSeq markers on Diamond linkage groups in centiMorgans (cM); the y-axis indicates the physical position of markers on sorghum chromosomes in megabases. Each dot corresponds to a RADSeq marker. **d** Comparison between Cavalier linkage groups and sorghum chromosomes

We investigated synteny relationships between *Z. matrella* and rice genomes. Consensus sequences of the mapped RADSeq markers were blasted against the genome sequences and gene models of the rice genome. Markers showing significant hits were then extracted for comparison study. Among the 2,375 RAD tags positioned on Diamond LGs, 413 (17.4 % of the mapped RAD tags) were found to have significant sequence similarities to gene models or genome sequences on the rice chromosomes (Additional file 7: Table S4). Among the 3,563 RAD tags mapped on Cavalier LGs, 684 (19.2 % of the mapped tags) can be associated with gene models or genome sequences on the rice chromosomes (Additional file 8: Table S5). In order to have a whole genome comparison view of the two species, we generated a Circos plot (Fig. 2) and a dot plot (Fig. 3a and b) by plotting the linkage map positions of *Z. matrella* RAD markers against the physical positions of aligned sequences in the rice genome. The dot plot and the Circos plot illustrated considerable collinearity between the two genomes. For each rice chromosome, we identified two corresponding LGs or regions in *Z. matrella* genome, consistent with an expected 1:2 ratio between the diploid rice and tetraploid *Z. matrella* genomes (Figs. 2 and 3a, b). The high levels of conservation of gene content and orders along the entire chromosome were observed between eight rice chromosomes and their orthologous chromosomes in *Z. matrella* (Figs. 2 and 3a, b). The remaining four rice chromosomes, Os2, Os6, Os9, and Os10, were involved in chromosome fusion events reducing the base chromosome number of *n* = 12 in the paleo-ancestor genome to *n* = 10 in *Z. matrella* genome (Figs. 2 and 3a, b). *Z. matrella* LG7 and its homologous LG8 correspond to two rice chromosomes, Os2 and Os10. The two arms of LG7 and 8 are orthologous to Os2, while the middle sections of LG7 and LG8 correspond to a truncated Os10 (Figs. 2 and 3a, b). We couldn’t identify the orthologous regions to the distal ends of Os10 in *Z. matrella* genome, suggesting the terminal parts of Os10 might have been lost during evolution. Similarly, *Z. matrella* LG19 and its homologous LG20 correspond to two rice chromosomes, Os6 and Os9. The two arms of LG19 and 20 are orthologous to Os6, while the middle sections of LG19 and LG20 correspond to Os9. Our comparative analysis indicated the paleo-ancestor genome with the base chromosome number of *n* = 12 had undergone two nested chromosomal fusion (NCF) events to reduce the base chromosome number from 12 to 10 in *Z. matrella* genome. Since rice genome has largely preserved the paleo-ancestral karyotype, we numbered the paleo-ancestral chromosomes (ρ1, ρ2…ρ12) based on rice karyotype. Therefore, two paleo-ancestral chromosomes ρ2 and ρ6 were invaded by paleo-ancestral chromosomes ρ10 and ρ9 in the centromeric regions, respectively, in the two NCF events reducing the base chromosome number from 12 to 10 in *Z. matrella* genome.

*Z. matrella* and Sorghum are closely related plant species and belong to sister subfamilies, Chloridoideae and Panicoideae, respectively, under PACCMAD clade. Like *Z. matrella*, sorghum has a base chromosome number of *n* = 10. Did the same pairs of ancestral chromosomes fuse in the karyotype evolution of the sorghum genome as fused in the *Z. matrella* lineage? To address this question, we conducted comparative genomics analysis between *Z. matrella* and sorghum genomes.

We identified anchor points for comparative analysis by searching the consensus sequences of the mapped RAD tags on *Z. matrella* genetic maps against sorghum genome sequences and gene models. Among the 3,563 RAD tags mapped on the Cavalier LGs, 801 (22.5 %) of them can be placed on sorghum chromosomes (Additional file 9: Table S6). Among the 2,375 RAD markers positioned on the Diamond LGs, 495 (20.8 %) of them can be found homologous positions on sorghum chromosomes (Additional file 10: Table S7). Dot-plot diagrams were drawn by plotting the genetic positions of anchored markers on *Z. matrella* maps against the physical positions of their homologs on sorghum chromosomes (Fig. 3c, d). The dot-plot diagrams revealed extensive collinearity along chromosome arms between genomes of *Z. matrella* and *S. bicolor*, but interruption of collinearity occurred at centromeric and pericentromeric regions of sorghum chromosomes for each pair of orthologous chromosomes. For each sorghum chromosome, we identified two orthologous LGs in *Z. matrella*, reinforcing the tetraploid genome nature of *Z. matrella*. We numbered the *Z. matrella* LGs according to their homology to sorghum chromosomes to simplify the synteny relationship for comparative genomics studies.

Comparative genomics analysis has revealed that sorghum chromosomes Sb1 and Sb2 derived from the paleo-ancestral chromosomes ρ10 and ρ3, and ρ9 and ρ7, respectively, via NCFs [26, 29]. These two NCF events, ρ3- ρ10- ρ3 and ρ7- ρ9- ρ7, occurred in sorghum lineage are different with the two NCF events we observed in the *Z. matrella* lineage, ρ2- ρ10- ρ2 and ρ6- ρ9- ρ6. Our comparative analysis between *Z. matrella* and sorghum revealed genome rearrangements occurred between Sb1 and Sb4 (orthologous of ρ2), and between Sb2 and Sb10 (orthologous of ρ6) (Fig. 3c, d), reinforcing our observations that the two NCF events occurred independently in *Z. matrella* and sorghum lineages during the karyotype evolution. Interestingly, the paleo-ancestral chromosomes ρ9 and ρ10 served as invading chromosomes in the NCF events in both *Z. matrella* and sorghum lineages. Table 3 summarizes detailed synteny relationships between *Z. matrella* LGs and sorghum and rice chromosomes.

**Table 3:** Syntenic relationships between *Zoysia matrella* LGs and rice and sorghum chromosomes. Numbers of markers that can be positioned on rice and sorghum chromosomes were present and the number of markers that hit rice or sorghum gene models were present in the bracket

Diamond LG	Cavalier LG	Orthologous rice chromosome	Number of markers that can be positioned on rice chromosomes		Orthologous sorghum chromosome	Number of markers that can be positioned on sorghum chromosomes	
			Diamond	Cavalier		Diamond	Cavalier
LG 01, 02	LG 01, 02	Os03	58(47)	122(108)	Sb01	79(57)	155(131)
LG 03, 04	LG 03, 04	Os07	34(27)	36(31)	Sb02	35(34)	47(37)
LG 05, 06	LG 05, 06	Os01	48(39)	89(76)	Sb03	60(44)	98(86)
LG 07, 08	LG 07, 08	Os02 + Os10	54(41)	76(63)	Sb04 + Sb01	75(60)	87(77)
LG 09, 10	LG 09, 10	Os11	21(19)	36(32)	Sb05	25(21)	38(33)
LG 11, 12	LG 11, 12	Os04	52(44)	79(60)	Sb06	60(53)	95(83)
LG 13, 14	LG 13, 14	Os08	46(33)	43(38)	Sb07	48(41)	44(35)
LG 15, 16	LG 15, 16	Os12	33(28)	58(51)	Sb08	40(34)	69(60)
LG 17, 18	LG 17, 18	Os05	33(30)	55(44)	Sb09	38(35)	66(57)
LG 19, 20	LG 19, 20	Os06 + Os09	30(22)	85(70)	Sb10 + Sb02	36(27)	101(86)
Total			409(330)	679(573)		496(406)	800(686)

Besides inter-chromosome rearrangements, dot-plot also revealed few intra-chromosomal rearrangements between *Z. matrella* and sorghum genomes. One inversion event was observed near one ends of LG5 and LG6 when the comparison was done between *Z. matrella* and sorghum genomes, but not between *Z. matrella* and rice genomes. This inversion event was also revealed by the comparison between sorghum and rice genomes. Similarly, another two inversion events were identified between *Z. matrella* LG7 and LG8 and their corresponding sorghum chromosome Sb4, and between Z*. matrella* LG13 and LG14 and their corresponding sorghum chromosome Sb7. These two inversion events were also observed when we compared sorghum chromosomes and their orthologous chromosomes in rice genome, but not between *Z. matrella* LGs and their rice homologous chromosomes.

### Identification of QTLs associated with resistance to FAW

We evaluated the FAW resistance in the whole mapping population of 221 individuals using in vitro insect feeding bioassays. The bioassay for the whole population was split into 8 experiments and the two parents and a susceptible zoysiagrass genotype, DALZ 8516, were included in each experiment. The data between experiments were normalized using the susceptible genotype, DALZ 8516, by Abbott’s Formula [24]. The mortality rates of larvae that were fed with leaf tissue of each individual of the mapping population were recorded at 7-, 10-, and 17-day. Significant difference in resistance to FAW between the two parents was observed during the whole procedure of in vitro insect feeding bioassay. Overall, the frequency distribution of the mortality rate of larvae fed leaf tissue from each individual of the mapping population exhibited non-normal distribution (Additional file 11: Figure S4). After 7 days of feeding, the mortality of larvae that were fed leaf tissue from the FAW resistant parent Cavalier was 57.7 % versus 1.7 % for the sensitive parent Diamond. The F1 population showed a skewed distribution toward FAW sensitive (Additional file 11: Figure S4A). A total of 88 F1 (39.8 % of the whole population) exhibited similar level of lethality rate (<10 %) as the FAW sensitive parent Diamond at 7-day. While, a total of 33 F1 individuals (14.9 % of the whole population) showed higher lethality rates than the FAW resistant parent Cavalier, indicating transgressive segregation of the mapping population for FAW resistance. After 10 days of feeding, the mortality of larvae that were fed leaf tissue from the FAW resistant parent Cavalier was at 87.47 %, a significant increase over the data collected at 7-day. For the FAW sensitive parent Diamond, the mortality of larvae remained low at 3.97 %. Among the 221 F1 individuals, almost equal number of individuals exhibited extreme tolerance and extreme sensitive to FAW (Additional file 11: Figure S4B). After 17 days of feeding, the mortality rate of larvae that were fed leaf tissue from Cavalier had increased to 100 %, while these that fed on Diamond tissue remained at similar levels as the one collected at 10-day (we want to clarify that the slightly lower mortality rate on Diamond at 17-day was caused by data normalization.). The frequency distribution of the mortality rate of larvae fed leaf tissue from the F1 individuals shifted toward FAW resistance at 17-day (Additional file 11: Figure S4C).

**Fig. 4:**
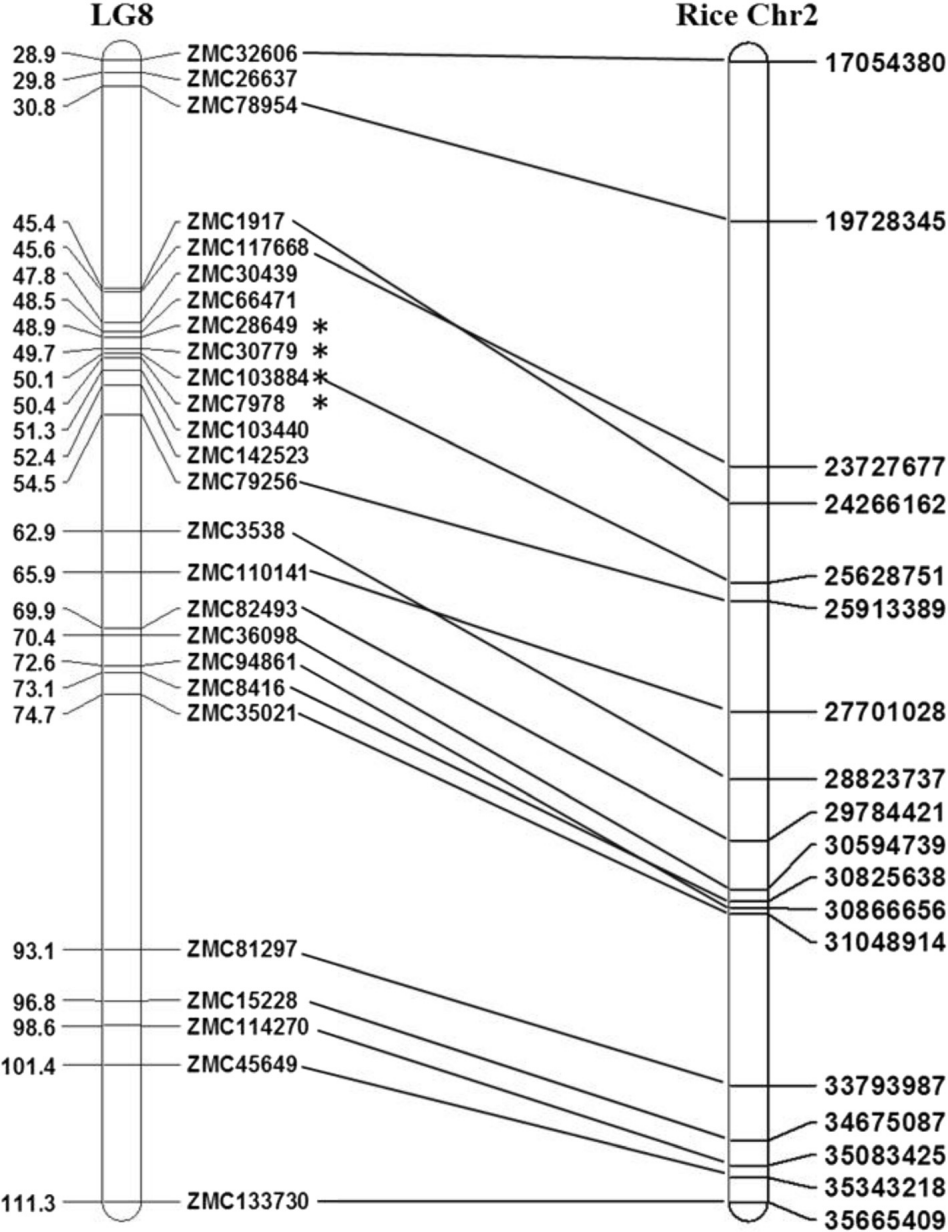
A syntenic block on rice chromosome Os2 (23,727,677–25,913,389) was identified corresponding to the major QTL region of FAW resistance in Cavalier

We took non-parametric genomic scan based on Kruskal-Wallis (KW) test to identify loci statistically (*P* < 0.001) associated with FAW resistance. The data sets of mortality rates of larvae collected at 7-, 10-, and 17-day were subjected to single marker association analysis individually. Using the data set collected at 7-day, two loci, 48.9 cM on LG8 and 71.9 cM on LG20, were detected to be significantly associated with FAW resistance (*P* < 0.0005) (Table 4). Using the data set collected at 10-day, we identified three additional loci, 49.7 cM and 50 cM on LG8 and 27.3 cM on LG20, besides the two loci detected by the 7-day data set (Table 4). The 17-day data set detected three loci on LG8 with a significance level at *P* < 0.001 (Table 4). The locus at 48.9 cM on LG8 was consistently detected by all three data set, indicating this locus play important roles in resistance to FAW at a wide range of insect growth and developmental stages. The locus at 71.9 cM on LG20 was detected by both 7-day and 10-day data sets, but not detected by 17-day data set, suggesting this locus might provide resistance to FAW at the relatively early insect growth and developmental stages. Similarly, two loci on LG8 at 50 cM and 50.3 cM were detected by 10-day and 17-day data sets, not by 7-day data set, suggesting this locus might provide resistance to FAW at the relatively late insect growth and developmental stages. Three loci on LG8 ranging within 1.4 cM (48.9 cM to 50.3 cM) were detected to be significantly associated with FAW resistance, providing us a major target for cloning genes controlling FAW resistance. To saturate the major QTL region on LG8, we mapped the distorted markers back on LG8 and the order of markers remained same after adding distorted markers (Additional file 12: Figure S5). The major QTL region, 48.9 cM to 50.3 cM on LG8, was aligned on rice genome (Fig. 4). A syntenic block on rice chromosome Os2 (23,727,677–25,913,389) was identified corresponding to the major QTL region.

**Table 4:** List of QTLs detected to be significantly associated with FAW resistance in zoysiagrass Cavalier

Trait	LG	Position (cM)	Locus	K	Significance levels
7-d-old larvae mortality rate	8	48.933	ZMC28649	12.28	******
	20	71.851	ZMC123031	13.111	******
10-d-old larvae mortality rate	8	48.933	ZMC28649	13.44	******
	20	27.274	ZMC37044	14.601	******
	20	71.851	ZMC123031	12.963	******
	8	49.689	ZMC30779	11.368	*****
	8	50.052	ZMC103884	11.258	*****
17-d-old larvae mortality rate	8	48.933	ZMC28649	11.458	*****
	8	50.052	ZMC103884	10.97	*****
	8	50.373	ZMC7978	11.684	*****

*LG*, linkage group, K: the Kruskal-Wallis test statistic; *****: *P* < 0.001; ******: *P* < 0.0005

## Discussion

Grasses are the most important plant family to humans. They not only provide more than ¾ of our food, but also serve as a major producer of our oxygen due to their ecological dominance, wide geographic range, and enormous biomass. The Chloridoideae, consisting of more than 1,600 species, is one of the three largest subfamilies of the grasses, along with Pooideae and Panicoideae [31]. Chloridoideae species share unusual features of leaf anatomy, and many of them exhibit extreme levels of tolerance to drought and high soil salinity. Furthermore, the transition from C3 to C4 photosynthesis that confers ecological success in many biomes firstly occurred in subfamily Chloridoideae, approximately 32.0–25.0 Mya [32]. And the subfamily Chloridoideae has been identified as the largest wholly C4 clade in plants [33]. Despite their ecological and economic importance, Chloridoideae species have received little attention on genomics studies, resulting in limited genetic and genomic information available for this important plant family. As a Chloridoideae species, *Z. matrella* has a base chromosome number of *n* = 10 which represents the predominant karyotypic number among the Chloridoideae. The sequence-tagged high-density linkage maps of *Z. matrella* developed in the present study provide a high-resolution framework for understanding evolutionary processes of this underexplored plant family, and serve as a foundation for future comparative genomic studies to expand our understanding of the origin of C4 photosynthesis in grasses.

### Karyotype evolution in the subfamily Chloridoideae

**Fig. 5:**
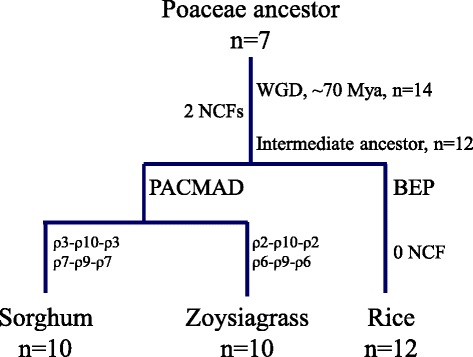
The karyotype evolution of Panicoideae and Chloridoideae subfamilies from the *n* = 12 intermediate ancestral genome

Karyotype evolution and genome size variation have been of significant interest, not only in terms of evolutionary relationships, but also in determining the evolutionary events that have shaped grass genomes. The two distinct evolutionary lineages in the grass family Poaceae, the BEP and PACCMAD clades, diverged from one another approximately 65–50 Mya [34, 35]. Comparative genomic analysis revealed that grasses evolved from an *n* = 7 ancestral karyotype, which subsequently underwent whole genome duplication (WGD) and chromosome fusion (CF) to become *n* = 12 [26, 27, 29, 36]. Compared with other existing grass species, the modern rice (*n* = 12) genome has retained the karyotype structure of the *n* = 12 ancestral genome the most [26, 27, 29, 36]. The *n* = 12 intermediate ancestral genome subsequently went through two NCFs, giving the progenitor genome of the Panicoideae ancestor with *n* = 10 [30, 36]. To elucidate the karyotype evolution from the *n* = 12 intermediate ancestral genome toward the karyotype of Chloridoideae, we compared the genome structure of *Z. matrella* to rice and sorghum. Our *Z. matrella*-rice-sorghum comparison studies revealed that the *Z. matrella* genome evolved from the *n* = 12 intermediate ancestral genome through two NCF events, ρ2-ρ10-ρ2 and ρ6-ρ9-ρ6, which are different from the two NCF events (ρ3- ρ10- ρ3 and ρ7- ρ9- ρ7) occurred in sorghum genome. Both Panicoideae and Chloridoideae ancestors were derived from the paleo-ancestor of *n* = 12 though two NCF events. However, different sets of paleo-ancestral chromosomes were involved in the NCF events, indicating different karyotype evolutionary paths led to the separation of the two sister subfamilies, Panicoideae and Chloridoideae. In the process of karyotype evolution in Chloridoideae, the paleo-ancestral chromosomes ρ9 and ρ10 served as ‘invading’ chromosomes and ρ2 and ρ6 served as ‘invaded’ chromosomes. While, two different chromosomes, ρ3 and ρ7, played as ‘invaded’ chromosomes in the process of karyotype evolution in Panicoideae although ρ9 and ρ10 still played as ‘invading’ chromosomes. Paleo-ancestral chromosomes ρ9 and ρ10 served as ‘invading’ chromosomes twice in the two distinct karyotype evolutionary pathways, indicating that their unique and common structure is favored in the NCF process. Interestingly, the two ‘invaded’ chromosomes, ρ2 and ρ6 in Chloridoideae, and ρ3 and ρ7 in Panicoideae, were paired homologous chromosomes, suggesting the NCF process might be facilitated by the specific structure of the complex formed between the paired homologous chromosomes. If our hypothesis is true, the two NCFs in both Panicoideae and Chloridoideae should be completed in a single step instead of two steps. In fact, there is no basic chromosome number of *n* = 11 observed in either Panicoideae or Chloridoideae [37], strongly supporting our hypothesis. In addition, the karyotype evolution analysis in this study is strongly supported by an earlier study using an independent population of *Z. japonica* [18]. Based on our results, we summarized the karyotype evolution of Panicoideae and Chloridoideae subfamilies from the *n* = 12 intermediate ancestral genome in Fig. 5.

### Genome collinearity between zoysiagrass and rice, and between zoysiagrass and sorghum

Modern grass genomes have diverged at the level of genome size tremendously since they evolved from a common ancestor. The common ancestor genome had undergone a genome-wide duplication approximately 70 Mya [38–40]. Diversification of the two main grass clades, BEP and PACCMAD, occurred approximately 55 Mya following the genome-wide duplication [41–43]. Both zoysiagrass and sorghum are under the PACCMAD clade and rice belongs to the BEP clade. Although zoysiagrass is evolutionarily closer to sorghum than rice, our comparative analysis revealed a higher level of conservation between zoysiagrass and rice than that between zoysiagrass and sorghum. The genome size of the ancestral genome at the base of Poales was reconstructed as 1C = 1.7 pg [44], and the genome size of rice was estimated at 1C = 0.43–0.46 pg [45], indicating extensive DNA loss during the diploidization following the genome-wide duplication. The genome sizes of zoysiagrass and sorghum were estimated by flow cytometry at 0.86 pg/2C [46] and 1.56–1.74 pg/2C [45], respectively. Zoysiagrass is a tetraploid genome, and both rice and sorghum are diploid genomes. Therefore, the monoploid genome size of zoysiagrass would be the smallest among the three genomes, approximately 0.215 pg, only half of the size of the monoploid genome of rice and a quarter of the monoploid genome of sorghum. In general, Chloridoideae species have relatively smaller genome sizes and a narrower range of chromosome sizes compared to Panicoideae species [44], which may suggest the two subfamilies followed two distinctive paths toward genome size evolution after they diverged from a common ancestor. Genome size differences in plants are mainly caused by amplification and removal of repetitive DNAs [47, 48]. Therefore, we predicted that zoysiagrass genome contained much less repetitive sequences than sorghum genome. Mutations and genetic rearrangements are frequently associated with repetitive sequences. Large chromosomal rearrangements can be caused by ectopic recombination between repeat sequences in different genomic regions [49]. Thus, the sorghum genome might have undergone more frequent genome rearrangements than did zoysiagrass due to its higher amount of repetitive sequences. On the other hand, the ancestor genome structure might have been better preserved in zoysiagrass than in sorghum.

### FAW resistance in zoysiagrass influenced by a major gene effect

High-resolution genetic map provides an essential tool for efficient detection of QTLs of economically and agronomically important traits. The accuracy of QTL mapping relies to some extent on the density of the genetic maps. In our present study, the genetic maps consisted of 2,375 and 3,563 SNP markers with average interval of 0.74 cM and 0.51 cM, respectively. Considering the genome size of zoysiagrass estimated at 421 Mbp [46], the average physical distance between adjacent markers is 177 kb for the Diamond map and 118 kb for the Cavalier map. The total lengths of the genetic maps are 1745.48 cM and 1824.94 cM for Diamond and Cavalier, respectively. Thus, the global ratio of physical distance (bp)/genetic distance (cM) is 241 kb/cM for the Diamond map and 231 kb/cM for the Cavalier map. The major QTL for FAW resistance was mapped on the 1.4 cM region (48.9 cM to 50.3 cM) of the LG8 of Cavalier map. Based on this estimation, the major target of FAW resistance encompasses approximately 320 kb.

Plant resistance to herbivores can be attributed to plant secondary metabolites and plant physical traits. Studies have shown that leaf tensile strength and lignin concentration were positively correlated with FAW resistance in varieties such as Cavalier and Emerald [50]. In addition, the secondary metabolite luteolin-3 was found in zoysiagrass that has an inverse relationship with FAW mortality, while luteolin-9 was positively correlated with FAW mortality in zoysiagrass [51]. We evaluated the FAW resistance in the whole mapping population of 221 individuals using in vitro no choice insect feeding bioassays. Our phenotype evaluation revealed a clearly non-normal distribution, implying the possibility that FAW resistance in zoysiagrass was influenced by specific genes with major effects. After 10 days of feeding, the ratio of extremely tolerant F1s to extremely sensitive F1s fitted 1:1 ratio, reinforcing our hypothesis that the FAW resistance in zoysiagrass is a quantitative trait with a major gene effect.

Our QTL analysis provides us a major target for cloning FAW resistance gene in zoysiagrass, which will serve as a basis for a better understanding of host resistance adaptations in zoysiagrass. The major QTLs associated with FAW resistance will be validated using different mapping populations and a large germplasm collection, which will help refine estimates of the genetic structure of FAW resistance in zoysiagrass. Molecular markers developed from the present study offer breeders a more efficient approach to select for targeted chromosome regions during zoysiagrass improvement efforts.

## Conclusions

We constructed sequence-based high-resolution genetic maps of *Z. matrella*. These maps offer valuable resource for comparative genomic analysis between *Z. matrella* and other plant species. Our comparative analysis revealed that *Z. matrella* shared a higher level of genome collinearity with rice than that with sorghum. Our result suggested that the sister subfamilies Chloridoideae and Panicoideae followed separate karyotype evolutionary pathways reducing the chromosome number from 12 to 10. These high-resolution maps also provide us important tools for detection of QTLs associated with economically and agronomically important traits. Six loci located on LGs 6, 8, and 20 were detected to be significantly associated with FAW resistance, providing us a major target for cloning FAW resistance gene in zoysiagrass.

## Abbreviations

BEP, Bambusoideae-Ehrhartoidea-Pooideae; CF, chromosome fusion; cM, centiMorgan; FAW, fall armyworm; KW, Kruskal-Wallis; LG, linkage group; Mya, million years ago; NCF, nested chromosomal fusion; NGS, next-generation sequencing; PACCMAD, Panicoideae-Aristidoideae-Centhothecoideae-Chloridoideae-Micrairoideae-Arundinoideae-Danthoideae; QTL, quantitative trait loci; RADSeq, restriction site-associated DNA sequencing; RCB, randomized complete block; SDA, single-dose allele; SDR, segregation distortion region; SNP, single nucleotide polymorphism; WGD, whole genome duplication

## Additional files

Additional file 1: Figure S1. The number of RADSeq reads of the parents and F1 progeny. (PDF 94 kb) Additional file 2: Table S1. Summary of RADSeq. Sample name, barcode, number of reads each individual of the mapping population are listed. (XLSX 17 kb) Additional file 3: Figure S2. Detailed genetic map of Diamond. Numbers on top of the maps: linkage group (LG); numbers on the left side of each LG: genetic distance (cM); numbers on the right side of each LG: marker name. (PDF 204 kb) Additional file 4: Table S2. Sequences of RAD tags mapped on Diamond linkage groups (LG). (XLSX 187 kb) Additional file 5: Figure S3. Detailed genetic map of Cavalier. Numbers on top of the maps: linkage group (LG); numbers on the left side of each LG: genetic distance (cM); numbers on the right side of each LG: marker name. (PDF 252 kb) Additional file 6: Table S3. Sequences of RAD tags mapped on Cavalier linkage groups (LG). (XLS 729 kb) Additional file 7: Table S4. Syntenic relationship of Diamond linkage groups and rice chromosomes. (XLS 119 kb) Additional file 8: Table S5. Syntenic relationship of Cavalier linkage groups and rice chromosomes. (XLS 177 kb) Additional file 9: Table S6. Syntenic relationship of Cavalier linkage groups and sorghum chromosomes. (XLS 202 kb) Additional file 10: Table S7. Syntenic relationship of Diamond linkage groups and sorghum chromosomes. (XLS 137 kb) Additional file 11: Figure S4. The frequency distribution of the mortality rate of larvae fed leaf tissue from each individual of the mapping population. D: cultivar Diamond (susceptible); C: cultivar Cavalier (resistant). (PDF 311 kb) Additional file 12: Figure S5. Cavalier LG8 without distorted marekrs and after adding distorted markers. 8: LG8 without distorted markers; 8’: LG8 after adding distorted markers. (PDF 179 kb)
