# Supplementary figures and images for: Construction of high-resolution genetic maps of Zoysia matrella (L.) Merrill and applications to comparative genomic analysis and QTL mapping of resistance to fall armyworm
Source: BMC Genomics. 2016 Aug 8;17:562. doi: 10.1186/s12864-016-2969-7 (PMC4977732; doi:10.1186/s12864-016-2969-7)

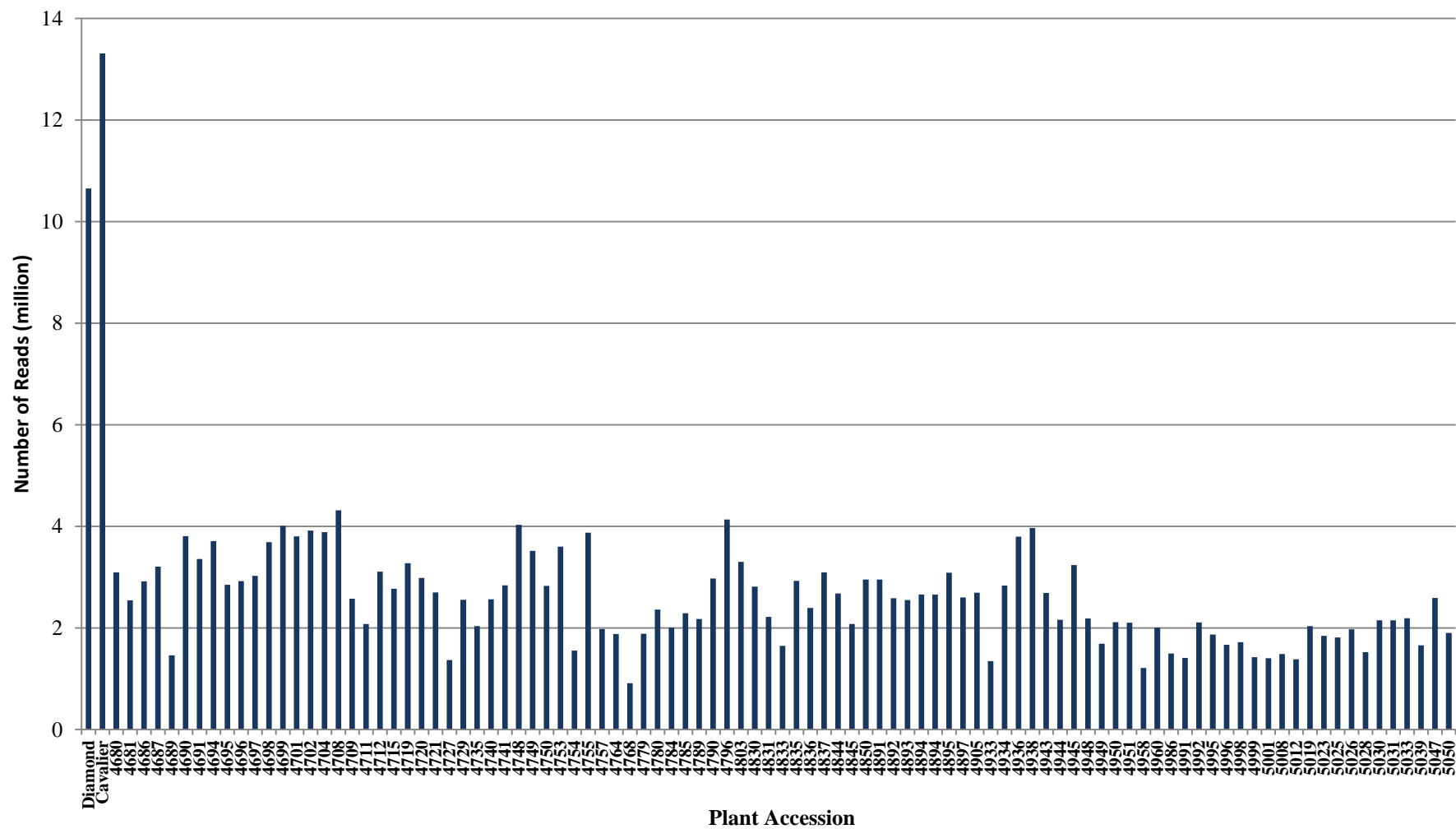

**Figure S1** The number of RADSeq reads of the parents and F1 progeny.

Supplement: Additional file 1: Figure S1. — The number of RADSeq reads of the parents and F1 progeny. (PDF 94 kb) [file 12864_2016_2969_MOESM1_ESM.pdf]
