# Supplementary material for: Construction of high-resolution genetic maps of Zoysia matrella (L.) Merrill and applications to comparative genomic analysis and QTL mapping of resistance to fall armyworm
Source: BMC Genomics. 2016 Aug 8;17:562. doi: 10.1186/s12864-016-2969-7 (PMC4977732; doi:10.1186/s12864-016-2969-7)
Supplement: Additional file 3: Figure S2. — Detailed genetic map of Diamond. Numbers on top of the maps: linkage group (LG); numbers on the left side of each LG: genetic distance (cM); numbers on the right side of each LG: marker name. (PDF 204 kb) [file 12864_2016_2969_MOESM3_ESM.pdf]

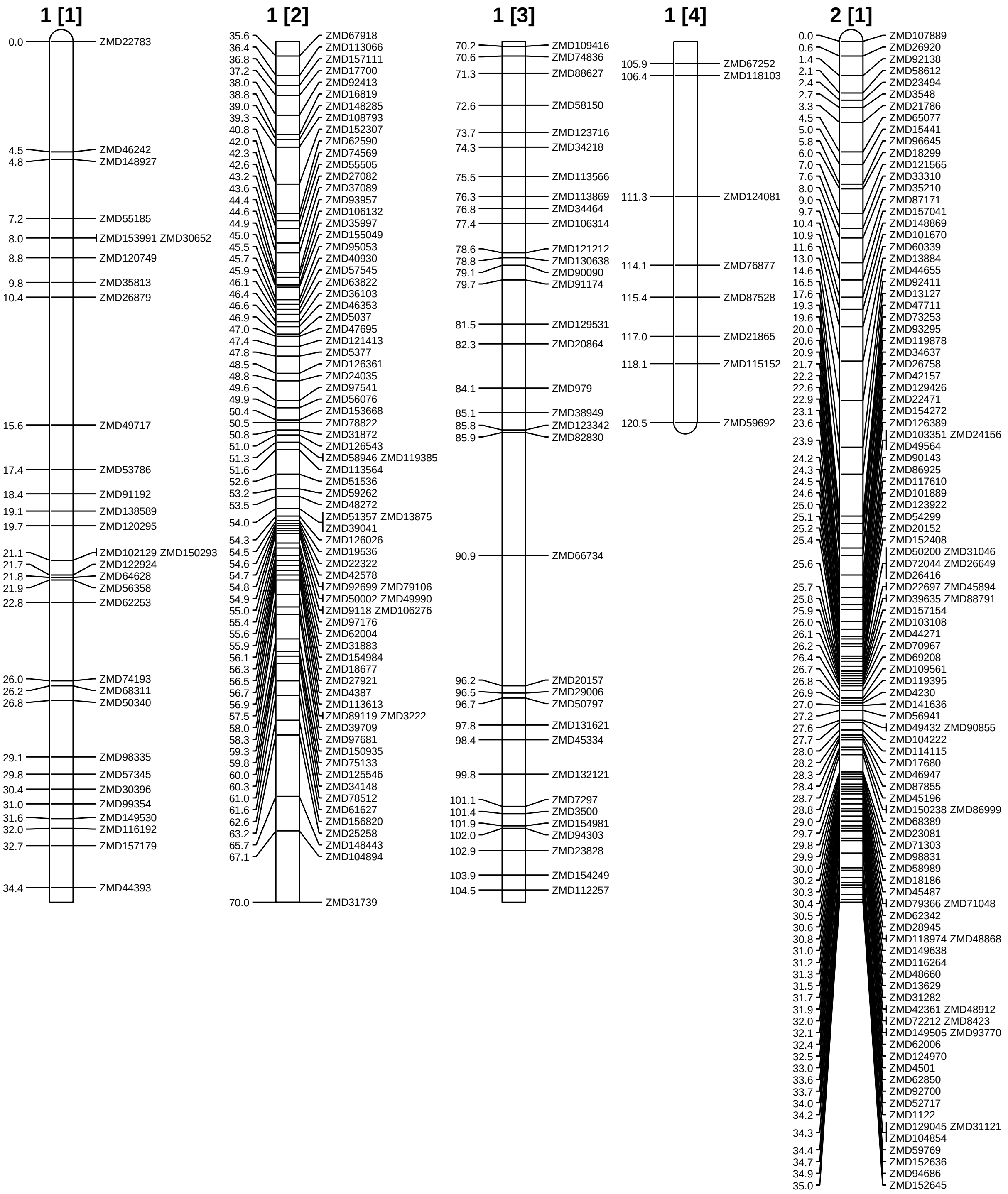

1 [1]
0.0 ZMD22783
4.5 ZMD46242
4.8 ZMD148927
7.2 ZMD55185
8.0 ZMD153991 ZMD30652
8.8 ZMD120749
9.8 ZMD35813
10.4 ZMD26879
15.6 ZMD49717
17.4 ZMD53786
18.4 ZMD91192
19.1 ZMD138589
19.7 ZMD120295
21.1 ZMD102129 ZMD150293
21.7 ZMD122924
21.8 ZMD64628
21.9 ZMD56358
22.8 ZMD62253
26.0 ZMD74193
26.2 ZMD68311
26.8 ZMD50340
29.1 ZMD98335
29.8 ZMD57345
30.4 ZMD30396
31.0 ZMD99354
31.6 ZMD149530
32.0 ZMD116192
32.7 ZMD157179
34.4 ZMD44393
1 [2]
35.6 ZMD67918
36.4 ZMD113066
36.8 ZMD157111
37.2 ZMD17700
38.0 ZMD92413
38.8 ZMD16819
39.0 ZMD148285
39.3 ZMD108793
40.8 ZMD152307
42.0 ZMD62590
42.3 ZMD74569
42.6 ZMD55505
43.2 ZMD27082
43.6 ZMD37089
44.4 ZMD93957
44.6 ZMD106132
44.9 ZMD35997
45.0 ZMD155049
45.5 ZMD95053
45.7 ZMD40930
45.9 ZMD57545
46.1 ZMD63822
46.4 ZMD36103
46.6 ZMD46353
46.9 ZMD5037
47.0 ZMD47695
47.4 ZMD121413
47.8 ZMD5377
48.5 ZMD126361
48.8 ZMD24035
49.6 ZMD97541
49.9 ZMD56076
50.4 ZMD153668
50.5 ZMD78822
50.8 ZMD31872
51.0 ZMD126543
51.3 ZMD58946 ZMD119385
51.6 ZMD113564
52.6 ZMD51536
53.2 ZMD59262
53.5 ZMD48272
54.0 ZMD51357 ZMD13875
ZMD39041
54.3 ZMD126026
54.5 ZMD19536
54.6 ZMD22322
54.7 ZMD42578
54.8 ZMD92699 ZMD79106
54.9 ZMD50002 ZMD49990
55.0 ZMD9118 ZMD106276
55.4 ZMD97176
55.6 ZMD62004
55.9 ZMD31883
56.1 ZMD154984
56.3 ZMD18677
56.5 ZMD27921
56.7 ZMD4387
56.9 ZMD113613
57.5 ZMD89119 ZMD3222
58.0 ZMD39709
58.3 ZMD97681
59.3 ZMD150935
59.8 ZMD75133
60.0 ZMD125546
60.3 ZMD34148
61.0 ZMD78512
61.6 ZMD61627
62.6 ZMD156820
63.2 ZMD25258
65.7 ZMD148443
67.1 ZMD104894
70.0 ZMD31739
1 [3]
70.2 ZMD109416
70.6 ZMD74836
71.3 ZMD88627
72.6 ZMD58150
73.7 ZMD123716
74.3 ZMD34218
75.5 ZMD113566
76.3 ZMD113869
76.8 ZMD34464
77.4 ZMD106314
78.6 ZMD121212
78.8 ZMD130638
79.1 ZMD90090
79.7 ZMD91174
81.5 ZMD129531
82.3 ZMD20864
84.1 ZMD979
85.1 ZMD38949
85.8 ZMD123342
85.9 ZMD82830
90.9 ZMD66734
96.2 ZMD20157
96.5 ZMD29006
96.7 ZMD50797
97.8 ZMD131621
98.4 ZMD45334
99.8 ZMD132121
101.1 ZMD7297
101.4 ZMD3500
101.9 ZMD154981
102.0 ZMD94303
102.9 ZMD23828
103.9 ZMD154249
104.5 ZMD112257
1 [4]
105.9 ZMD67252
106.4 ZMD118103
111.3 ZMD124081
114.1 ZMD76877
115.4 ZMD87528
117.0 ZMD21865
118.1 ZMD115152
120.5 ZMD59692
2 [1]
0.0 ZMD107889
0.6 ZMD26920
1.4 ZMD92138
2.1 ZMD58612
2.4 ZMD23494
2.7 ZMD3548
3.3 ZMD21786
4.5 ZMD65077
5.0 ZMD15441
5.8 ZMD96645
6.0 ZMD18299
7.0 ZMD121565
7.6 ZMD33310
8.0 ZMD35210
9.0 ZMD87171
9.7 ZMD157041
10.4 ZMD148869
10.9 ZMD101670
11.6 ZMD60339
13.0 ZMD13884
14.6 ZMD44655
16.5 ZMD92411
17.6 ZMD13127
19.3 ZMD47711
19.6 ZMD73253
20.0 ZMD93295
20.6 ZMD119878
20.9 ZMD34637
21.7 ZMD26758
22.2 ZMD42157
22.6 ZMD129426
22.9 ZMD22471
23.1 ZMD154272
23.6 ZMD126389
23.9 ZMD103351 ZMD24156
ZMD49564
24.2 ZMD90143
24.3 ZMD86925
24.5 ZMD117610
24.6 ZMD101889
25.0 ZMD123922
25.1 ZMD54299
25.2 ZMD20152
25.4 ZMD152408
25.6 ZMD50200 ZMD31046
ZMD72044 ZMD26649
ZMD26416
25.7 ZMD22697 ZMD45894
25.8 ZMD39635 ZMD88791
25.9 ZMD157154
26.0 ZMD103108
26.1 ZMD44271
26.2 ZMD70967
26.4 ZMD69208
26.7 ZMD109561
26.8 ZMD119395
26.9 ZMD4230
27.0 ZMD141636
27.2 ZMD56941
27.6 ZMD49432 ZMD90855
27.7 ZMD104222
28.0 ZMD114115
28.2 ZMD17680
28.3 ZMD46947
28.4 ZMD87855
28.7 ZMD45196
28.8 ZMD150238 ZMD86999
29.0 ZMD68389
29.7 ZMD23081
29.8 ZMD71303
29.9 ZMD98831
30.0 ZMD58989
30.2 ZMD18186
30.3 ZMD45487
30.4 ZMD79366 ZMD71048
30.5 ZMD62342
30.6 ZMD28945
30.8 ZMD118974 ZMD48868
31.0 ZMD149638
31.2 ZMD116264
31.3 ZMD48660
31.5 ZMD13629
31.7 ZMD31282
31.9 ZMD42361 ZMD48912
32.0 ZMD72212 ZMD8423
32.1 ZMD149505 ZMD93770
32.4 ZMD62006
32.5 ZMD124970
33.0 ZMD4501
33.6 ZMD62850
33.7 ZMD92700
34.0 ZMD52717
34.2 ZMD1122
34.3 ZMD129045 ZMD31121
ZMD104854
34.4 ZMD59769
34.7 ZMD152636
34.9 ZMD94686
35.0 ZMD152645

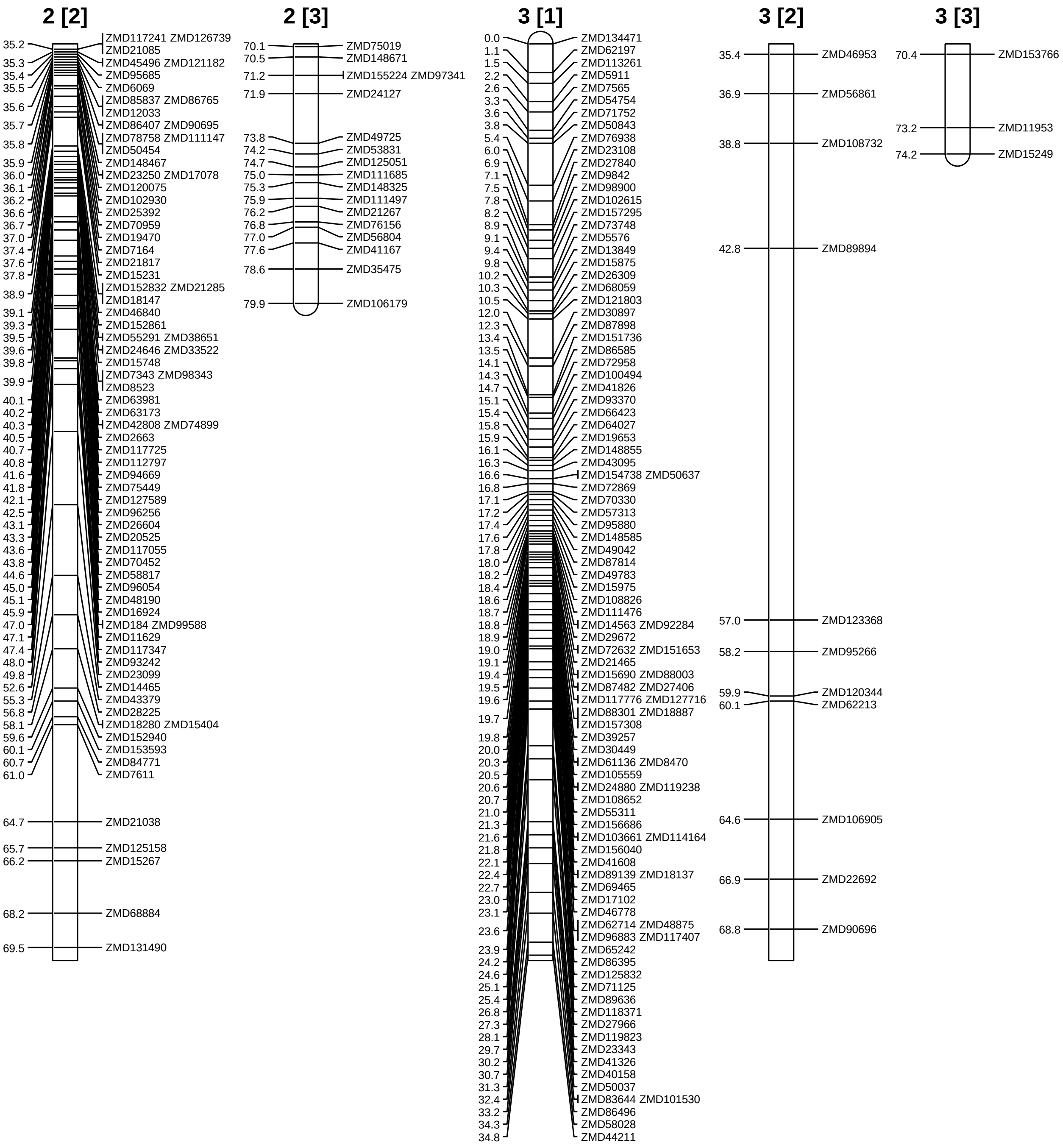
2 [2]
35.2
35.3
35.4
35.5
35.6
35.7
35.8
35.9
36.0
36.1
36.2
36.6
36.7
37.0
37.4
37.6
37.8
38.9
39.1
39.3
39.5
39.6
39.8
39.9
40.1
40.2
40.3
40.5
40.7
40.8
41.6
41.8
42.1
42.5
43.1
43.3
43.6
43.8
44.6
45.0
45.1
45.9
47.0
47.1
47.4
48.0
49.8
52.6
55.3
56.8
58.1
59.6
60.1
60.7
61.0
64.7
65.7
66.2
68.2
69.5
ZMD117241 ZMD126739
ZMD21085
ZMD45496 ZMD121182
ZMD95685
ZMD6069
ZMD85837 ZMD86765
ZMD12033
ZMD86407 ZMD90695
ZMD78758 ZMD111147
ZMD50454
ZMD148467
ZMD23250 ZMD17078
ZMD120075
ZMD102930
ZMD25392
ZMD70959
ZMD19470
ZMD7164
ZMD21817
ZMD15231
ZMD152832 ZMD21285
ZMD18147
ZMD46840
ZMD152861
ZMD55291 ZMD38651
ZMD24646 ZMD33522
ZMD15748
ZMD7343 ZMD98343
ZMD8523
ZMD63981
ZMD63173
ZMD42808 ZMD74899
ZMD2663
ZMD117725
ZMD112797
ZMD94669
ZMD75449
ZMD127589
ZMD96256
ZMD26604
ZMD20525
ZMD117055
ZMD70452
ZMD58817
ZMD96054
ZMD48190
ZMD16924
ZMD184 ZMD99588
ZMD11629
ZMD117347
ZMD93242
ZMD23099
ZMD14465
ZMD43379
ZMD28225
ZMD18280 ZMD15404
ZMD152940
ZMD153593
ZMD84771
ZMD7611
ZMD21038
ZMD125158
ZMD15267
ZMD68884
ZMD131490
2 [3]
70.1 ZMD75019
70.5 ZMD148671
71.2 ZMD155224 ZMD97341
71.9 ZMD24127
73.8 ZMD49725
74.2 ZMD53831
74.7 ZMD125051
75.0 ZMD111685
75.3 ZMD148325
75.9 ZMD111497
76.2 ZMD21267
76.8 ZMD76156
77.0 ZMD56804
77.6 ZMD41167
78.6 ZMD35475
79.9 ZMD106179
3 [1]
0.0
1.1
1.5
2.2
2.6
3.3
3.6
3.8
5.4
6.0
6.9
7.1
7.5
7.8
8.2
8.9
9.1
9.4
9.8
10.2
10.3
10.5
12.0
12.3
13.4
13.5
14.1
14.3
14.7
15.1
15.4
15.8
15.9
16.1
16.3
16.6
16.8
17.1
17.2
17.4
17.6
17.8
18.0
18.2
18.4
18.6
18.7
18.8
18.9
19.0
19.1
19.4
19.5
19.6
19.7
19.8
20.0
20.3
20.5
20.6
20.7
21.0
21.3
21.6
21.8
22.1
22.4
22.7
23.0
23.1
23.6
23.9
24.2
24.6
25.1
25.4
26.8
27.3
28.1
29.7
30.2
30.7
31.3
32.4
33.2
34.3
34.8
ZMD134471
ZMD62197
ZMD113261
ZMD5911
ZMD7565
ZMD54754
ZMD71752
ZMD50843
ZMD76938
ZMD23108
ZMD27840
ZMD9842
ZMD98900
ZMD102615
ZMD157295
ZMD73748
ZMD5576
ZMD13849
ZMD15875
ZMD26309
ZMD68059
ZMD121803
ZMD30897
ZMD87898
ZMD151736
ZMD86585
ZMD72958
ZMD100494
ZMD41826
ZMD93370
ZMD66423
ZMD64027
ZMD19653
ZMD148855
ZMD43095
ZMD154738 ZMD50637
ZMD72869
ZMD70330
ZMD57313
ZMD95880
ZMD148585
ZMD49042
ZMD87814
ZMD49783
ZMD15975
ZMD108826
ZMD111476
ZMD14563 ZMD92284
ZMD29672
ZMD72632 ZMD151653
ZMD21465
ZMD15690 ZMD88003
ZMD87482 ZMD27406
ZMD117776 ZMD127716
ZMD88301 ZMD18887
ZMD157308
ZMD39257
ZMD30449
ZMD61136 ZMD8470
ZMD105559
ZMD24880 ZMD119238
ZMD108652
ZMD55311
ZMD156686
ZMD103661 ZMD114164
ZMD156040
ZMD41608
ZMD89139 ZMD18137
ZMD69465
ZMD17102
ZMD46778
ZMD62714 ZMD48875
ZMD96883 ZMD117407
ZMD65242
ZMD86395
ZMD125832
ZMD71125
ZMD89636
ZMD118371
ZMD27966
ZMD119823
ZMD23343
ZMD41326
ZMD40158
ZMD50037
ZMD83644 ZMD101530
ZMD86496
ZMD58028
ZMD44211
3 [2]
35.4 ZMD46953
36.9 ZMD56861
38.8 ZMD108732
42.8 ZMD89894
57.0 ZMD123368
58.2 ZMD95266
59.9 ZMD120344
60.1 ZMD62213
64.6 ZMD106905
66.9 ZMD22692
68.8 ZMD90696
3 [3]
70.4 ZMD153766
73.2 ZMD11953
74.2 ZMD15249

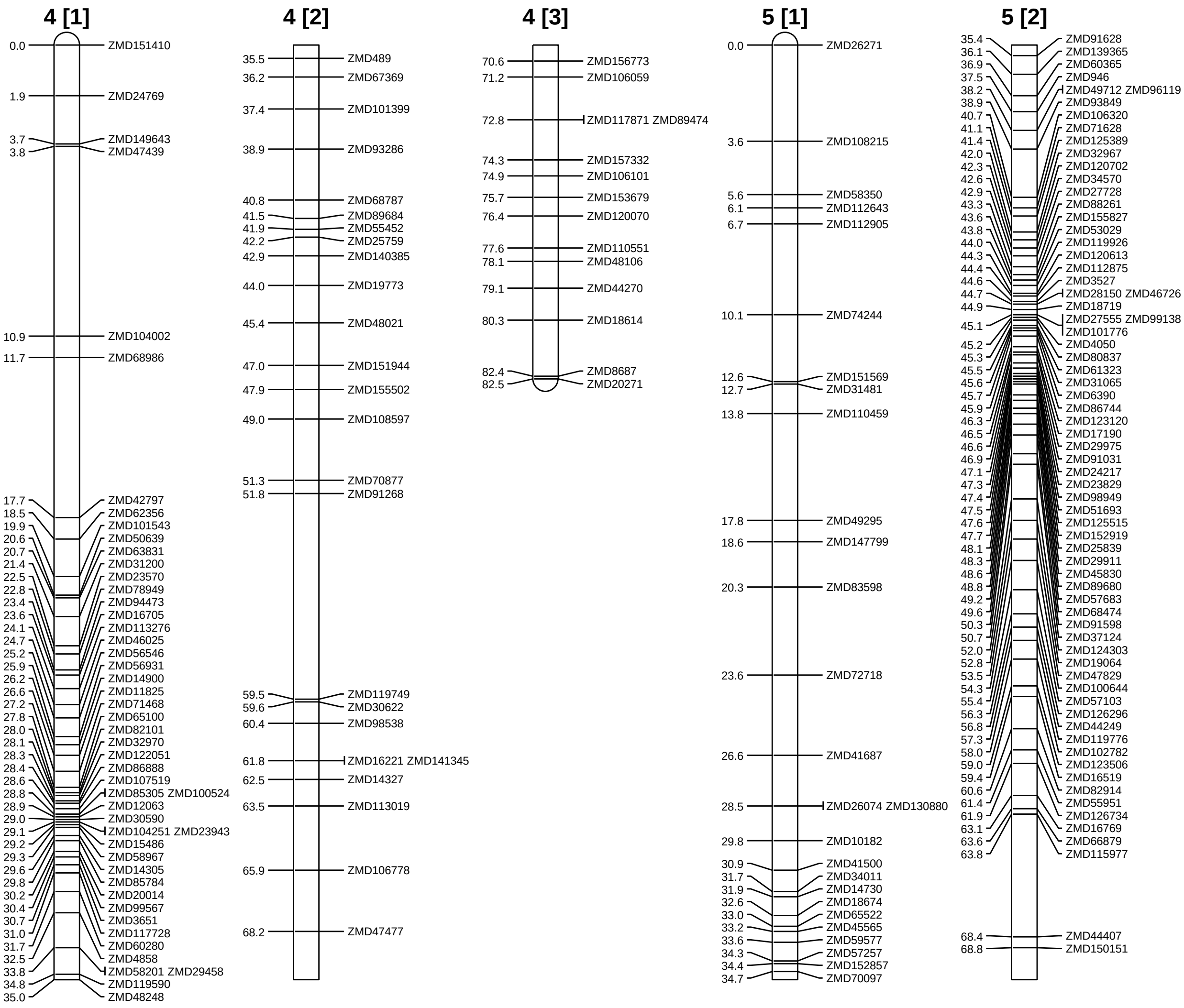

4 [1]
0.0 ZMD151410
1.9 ZMD24769
3.7 ZMD149643
3.8 ZMD47439
10.9 ZMD104002
11.7 ZMD68986
17.7 ZMD42797
18.5 ZMD62356
19.9 ZMD101543
20.6 ZMD50639
20.7 ZMD63831
21.4 ZMD31200
22.5 ZMD23570
22.8 ZMD78949
23.4 ZMD94473
23.6 ZMD16705
24.1 ZMD113276
24.7 ZMD46025
25.2 ZMD56546
25.9 ZMD56931
26.2 ZMD14900
26.6 ZMD11825
27.2 ZMD71468
27.8 ZMD65100
28.0 ZMD82101
28.1 ZMD32970
28.3 ZMD122051
28.4 ZMD86888
28.6 ZMD107519
28.8 ZMD85305 ZMD100524
28.9 ZMD12063
29.0 ZMD30590
29.1 ZMD104251 ZMD23943
29.2 ZMD15486
29.3 ZMD58967
29.6 ZMD14305
29.8 ZMD85784
30.2 ZMD20014
30.4 ZMD99567
30.7 ZMD3651
31.0 ZMD117728
31.7 ZMD60280
32.5 ZMD4858
33.8 ZMD58201 ZMD29458
34.8 ZMD119590
35.0 ZMD48248
4 [2]
35.5 ZMD489
36.2 ZMD67369
37.4 ZMD101399
38.9 ZMD93286
40.8 ZMD68787
41.5 ZMD89684
41.9 ZMD55452
42.2 ZMD25759
42.9 ZMD140385
44.0 ZMD19773
45.4 ZMD48021
47.0 ZMD151944
47.9 ZMD155502
49.0 ZMD108597
51.3 ZMD70877
51.8 ZMD91268
59.5 ZMD119749
59.6 ZMD30622
60.4 ZMD98538
61.8 ZMD16221 ZMD141345
62.5 ZMD14327
63.5 ZMD113019
65.9 ZMD106778
68.2 ZMD47477
4 [3]
70.6 ZMD156773
71.2 ZMD106059
72.8 ZMD117871 ZMD89474
74.3 ZMD157332
74.9 ZMD106101
75.7 ZMD153679
76.4 ZMD120070
77.6 ZMD110551
78.1 ZMD48106
79.1 ZMD44270
80.3 ZMD18614
82.4 ZMD8687
82.5 ZMD20271
5 [1]
0.0 ZMD26271
3.6 ZMD108215
5.6 ZMD58350
6.1 ZMD112643
6.7 ZMD112905
10.1 ZMD74244
12.6 ZMD151569
12.7 ZMD31481
13.8 ZMD110459
17.8 ZMD49295
18.6 ZMD147799
20.3 ZMD83598
23.6 ZMD72718
26.6 ZMD41687
28.5 ZMD26074 ZMD130880
29.8 ZMD10182
30.9 ZMD41500
31.7 ZMD34011
31.9 ZMD14730
32.6 ZMD18674
33.0 ZMD65522
33.2 ZMD45565
33.6 ZMD59577
34.3 ZMD57257
34.4 ZMD152857
34.7 ZMD70097
5 [2]
35.4 ZMD91628
36.1 ZMD139365
36.9 ZMD60365
37.5 ZMD946
38.2 ZMD49712 ZMD96119
38.9 ZMD93849
40.7 ZMD106320
41.1 ZMD71628
41.4 ZMD125389
42.0 ZMD32967
42.3 ZMD120702
42.6 ZMD34570
42.9 ZMD27728
43.3 ZMD88261
43.6 ZMD155827
43.8 ZMD53029
44.0 ZMD119926
44.3 ZMD120613
44.4 ZMD112875
44.6 ZMD3527
44.7 ZMD28150 ZMD46726
44.9 ZMD18719
45.1 ZMD27555 ZMD99138
ZMD101776
45.2 ZMD4050
45.3 ZMD80837
45.5 ZMD61323
45.6 ZMD31065
45.7 ZMD6390
45.9 ZMD86744
46.3 ZMD123120
46.5 ZMD17190
46.6 ZMD29975
46.9 ZMD91031
47.1 ZMD24217
47.3 ZMD23829
47.4 ZMD98949
47.5 ZMD51693
47.6 ZMD125515
47.7 ZMD152919
48.1 ZMD25839
48.3 ZMD29911
48.6 ZMD45830
48.8 ZMD89680
49.2 ZMD57683
49.6 ZMD68474
50.3 ZMD91598
50.7 ZMD37124
52.0 ZMD124303
52.8 ZMD19064
53.5 ZMD47829
54.3 ZMD100644
55.4 ZMD57103
56.3 ZMD126296
56.8 ZMD44249
57.3 ZMD119776
58.0 ZMD102782
59.0 ZMD123506
59.4 ZMD16519
60.6 ZMD82914
61.4 ZMD55951
61.9 ZMD126734
63.1 ZMD16769
63.6 ZMD66879
63.8 ZMD115977
68.4 ZMD44407
68.8 ZMD150151

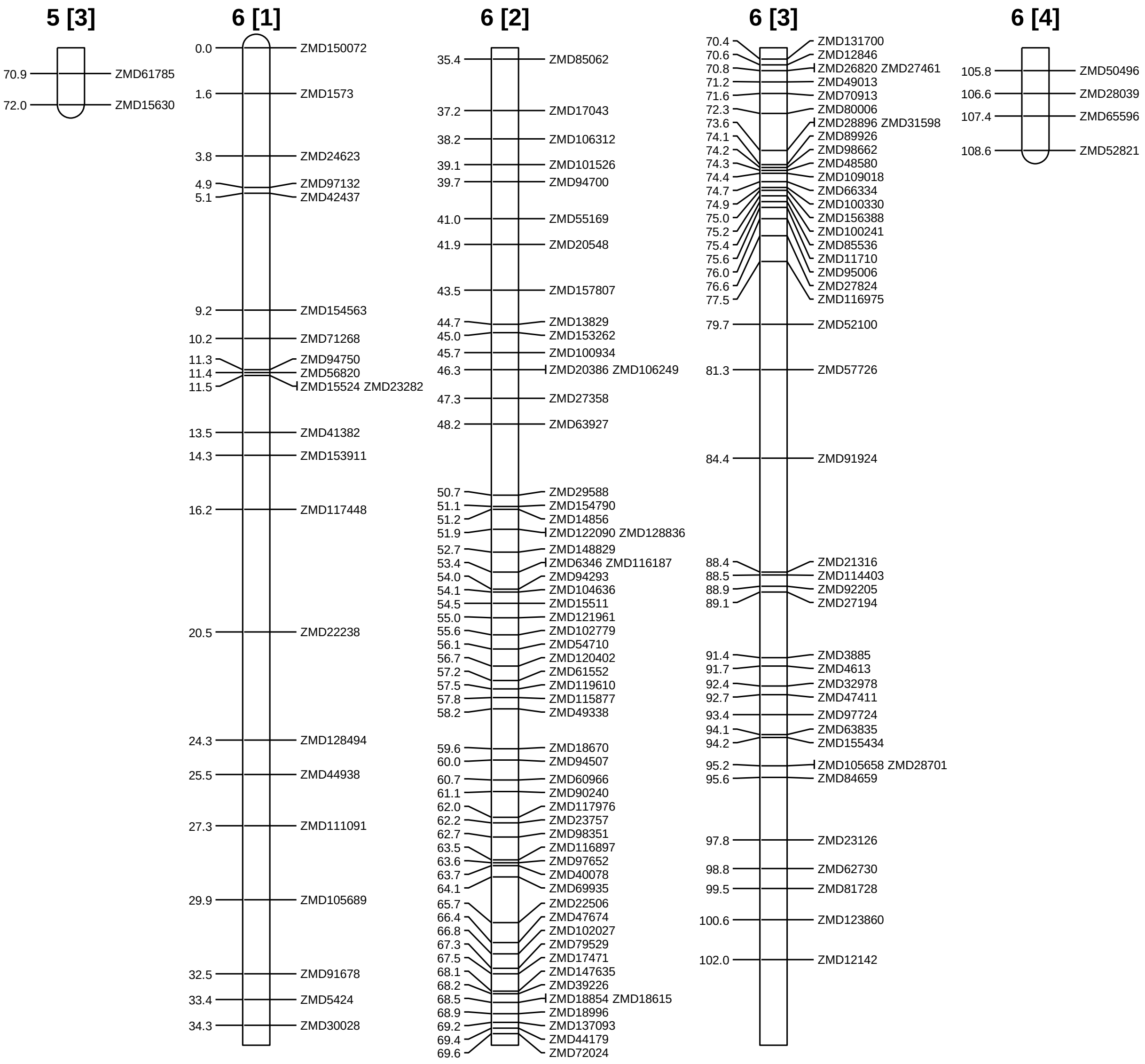
5 [3]
70.9 ZMD61785
72.0 ZMD15630
6 [1]
0.0 ZMD150072
1.6 ZMD1573
3.8 ZMD24623
4.9 ZMD97132
5.1 ZMD42437
9.2 ZMD154563
10.2 ZMD71268
11.3 ZMD94750
11.4 ZMD56820
11.5 ZMD15524 ZMD23282
13.5 ZMD41382
14.3 ZMD153911
16.2 ZMD117448
20.5 ZMD22238
24.3 ZMD128494
25.5 ZMD44938
27.3 ZMD111091
29.9 ZMD105689
32.5 ZMD91678
33.4 ZMD5424
34.3 ZMD30028
6 [2]
35.4 ZMD85062
37.2 ZMD17043
38.2 ZMD106312
39.1 ZMD101526
39.7 ZMD94700
41.0 ZMD55169
41.9 ZMD20548
43.5 ZMD157807
44.7 ZMD13829
45.0 ZMD153262
45.7 ZMD100934
46.3 ZMD20386 ZMD106249
47.3 ZMD27358
48.2 ZMD63927
50.7 ZMD29588
51.1 ZMD154790
51.2 ZMD14856
51.9 ZMD122090 ZMD128836
52.7 ZMD148829
53.4 ZMD6346 ZMD116187
54.0 ZMD94293
54.1 ZMD104636
54.5 ZMD15511
55.0 ZMD121961
55.6 ZMD102779
56.1 ZMD54710
56.7 ZMD120402
57.2 ZMD61552
57.5 ZMD119610
57.8 ZMD115877
58.2 ZMD49338
59.6 ZMD18670
60.0 ZMD94507
60.7 ZMD60966
61.1 ZMD90240
62.0 ZMD117976
62.2 ZMD23757
62.7 ZMD98351
63.5 ZMD116897
63.6 ZMD97652
63.7 ZMD40078
64.1 ZMD69935
65.7 ZMD22506
66.4 ZMD47674
66.8 ZMD102027
67.3 ZMD79529
67.5 ZMD17471
68.1 ZMD147635
68.2 ZMD39226
68.5 ZMD18854 ZMD18615
68.9 ZMD18996
69.2 ZMD137093
69.4 ZMD44179
69.6 ZMD72024
6 [3]
70.4 ZMD131700
70.6 ZMD12846
70.8 ZMD26820 ZMD27461
71.2 ZMD49013
71.6 ZMD70913
72.3 ZMD80006
73.6 ZMD28896 ZMD31598
74.1 ZMD89926
74.2 ZMD98662
74.3 ZMD48580
74.4 ZMD109018
74.7 ZMD66334
74.9 ZMD100330
75.0 ZMD156388
75.2 ZMD100241
75.4 ZMD85536
75.6 ZMD11710
76.0 ZMD95006
76.6 ZMD27824
77.5 ZMD116975
79.7 ZMD52100
81.3 ZMD57726
84.4 ZMD91924
88.4 ZMD21316
88.5 ZMD114403
88.9 ZMD92205
89.1 ZMD27194
91.4 ZMD3885
91.7 ZMD4613
92.4 ZMD32978
92.7 ZMD47411
93.4 ZMD97724
94.1 ZMD63835
94.2 ZMD155434
95.2 ZMD105658 ZMD28701
95.6 ZMD84659
97.8 ZMD23126
98.8 ZMD62730
99.5 ZMD81728
100.6 ZMD123860
102.0 ZMD12142
6 [4]
105.8 ZMD50496
106.6 ZMD28039
107.4 ZMD65596
108.6 ZMD52821

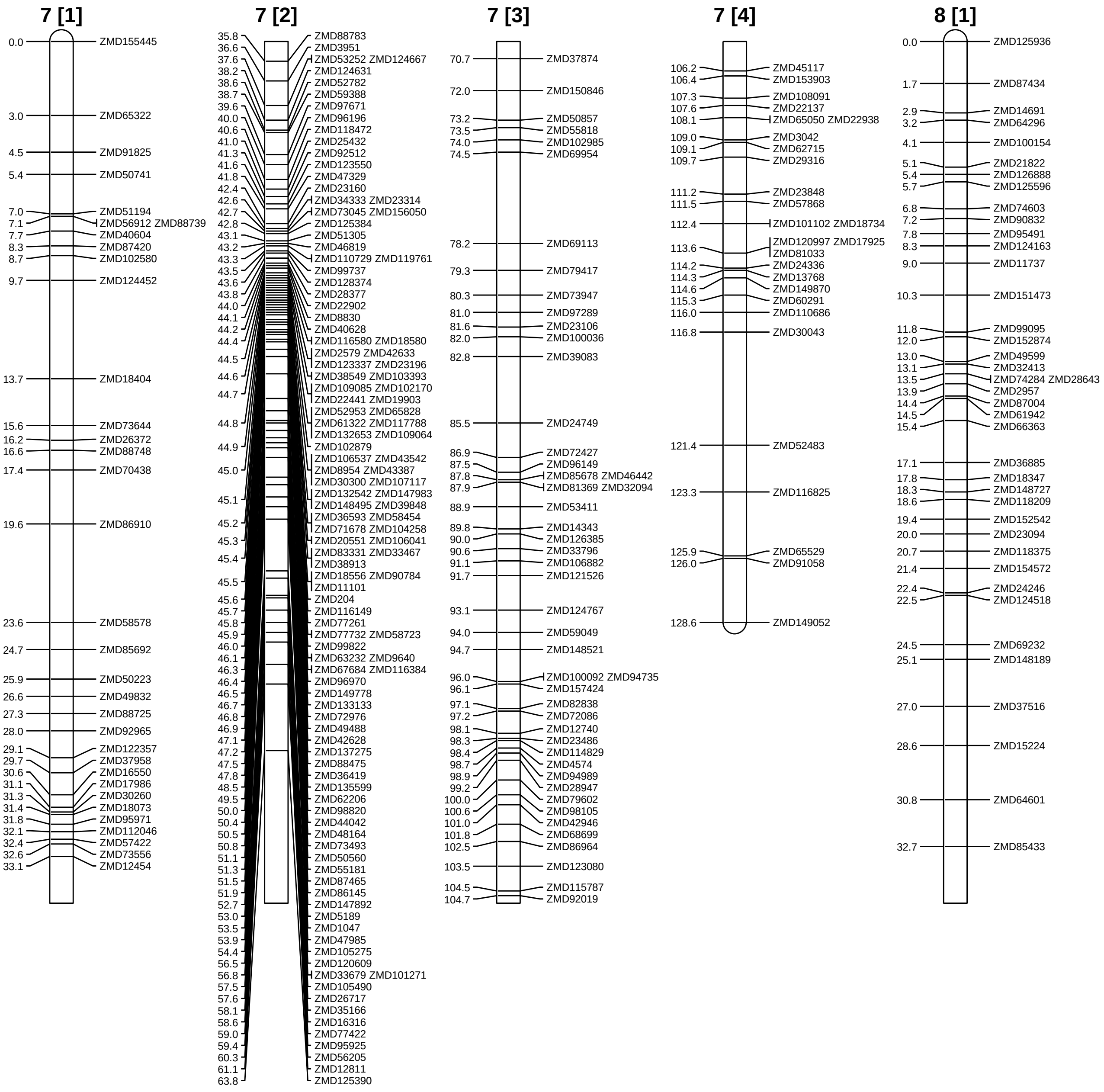
7 [1]
7 [2]
7 [3]
7 [4]
8 [1]

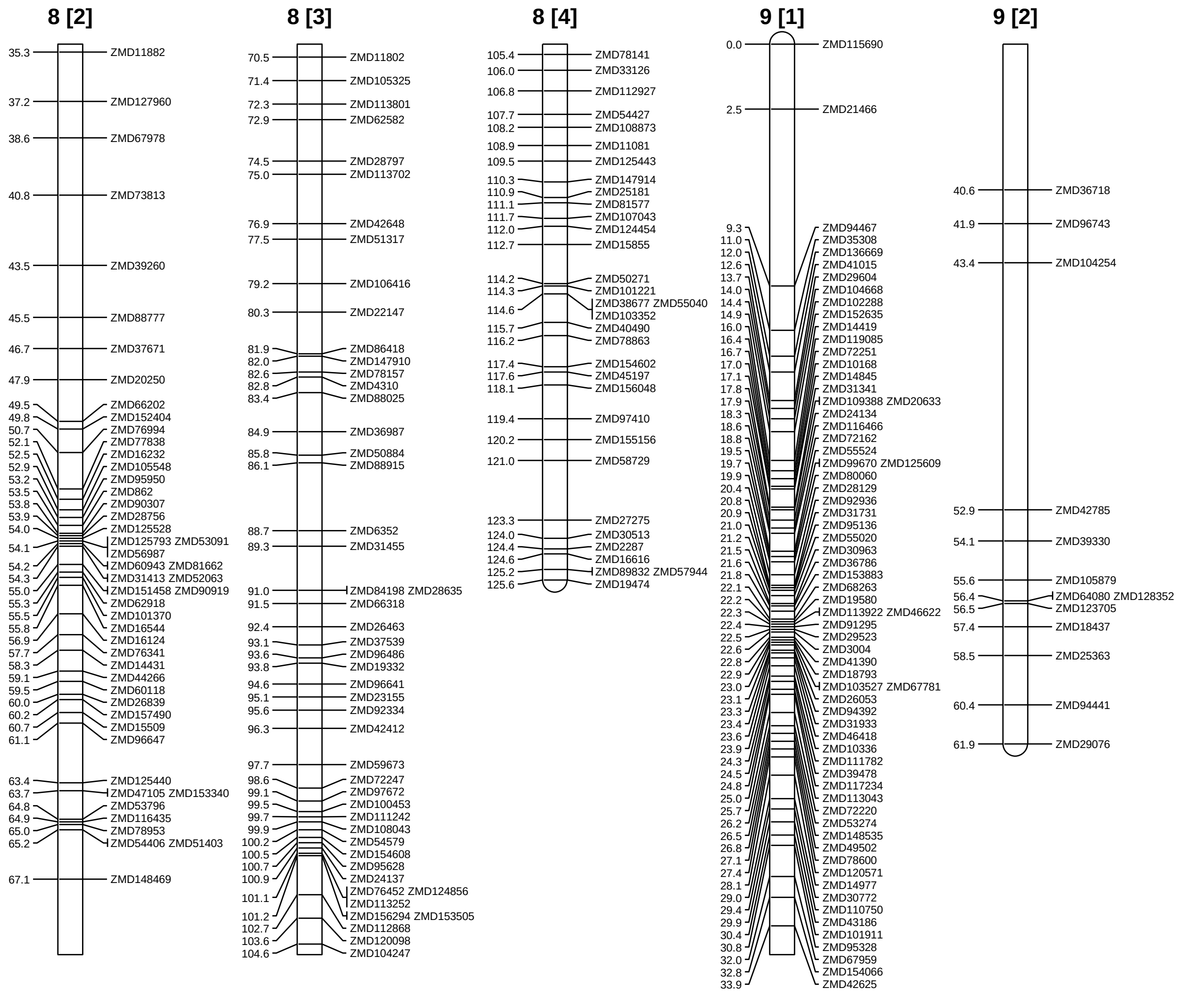
8 [2]
8 [3]
8 [4]
9 [1]
9 [2]

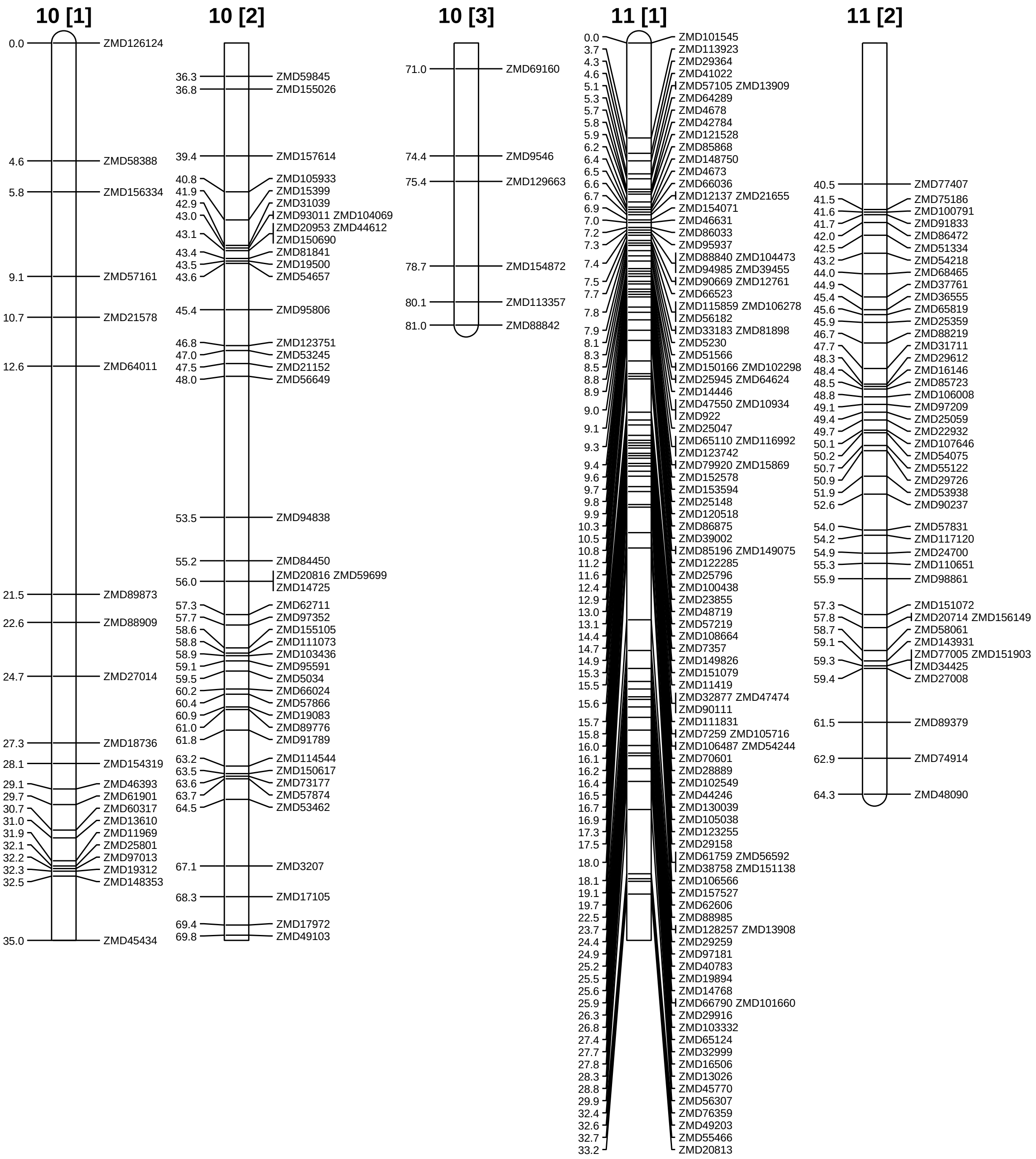
10 [1]
0.0 ZMD126124
4.6 ZMD58388
5.8 ZMD156334
9.1 ZMD57161
10.7 ZMD21578
12.6 ZMD64011
21.5 ZMD89873
22.6 ZMD88909
24.7 ZMD27014
27.3 ZMD18736
28.1 ZMD154319
29.1 ZMD46393
29.7 ZMD61901
30.7 ZMD60317
31.0 ZMD13610
31.9 ZMD11969
32.1 ZMD25801
32.2 ZMD97013
32.3 ZMD19312
32.5 ZMD148353
35.0 ZMD45434
10 [2]
36.3 ZMD59845
36.8 ZMD155026
39.4 ZMD157614
40.8 ZMD105933
41.9 ZMD15399
42.9 ZMD31039
43.0 ZMD93011 ZMD104069
43.1 ZMD20953 ZMD44612
ZMD150690
43.4 ZMD81841
43.5 ZMD19500
43.6 ZMD54657
45.4 ZMD95806
46.8 ZMD123751
47.0 ZMD53245
47.5 ZMD21152
48.0 ZMD56649
53.5 ZMD94838
55.2 ZMD84450
56.0 ZMD20816 ZMD59699
ZMD14725
57.3 ZMD62711
57.7 ZMD97352
58.6 ZMD155105
58.8 ZMD111073
58.9 ZMD103436
59.1 ZMD95591
59.5 ZMD5034
60.2 ZMD66024
60.4 ZMD57866
60.9 ZMD19083
61.0 ZMD89776
61.8 ZMD91789
63.2 ZMD114544
63.5 ZMD150617
63.6 ZMD73177
63.7 ZMD57874
64.5 ZMD53462
67.1 ZMD3207
68.3 ZMD17105
69.4 ZMD17972
69.8 ZMD49103
10 [3]
71.0 ZMD69160
74.4 ZMD9546
75.4 ZMD129663
78.7 ZMD154872
80.1 ZMD113357
81.0 ZMD88842
11 [1]
0.0
3.7
4.3
4.6
5.1
5.3
5.7
5.8
5.9
6.2
6.4
6.5
6.6
6.7
6.9
7.0
7.2
7.3
7.4
7.5
7.7
7.8
7.9
8.1
8.3
8.5
8.8
8.9
9.0
9.1
9.3
9.4
9.6
9.7
9.8
9.9
10.3
10.5
10.8
11.2
11.6
12.4
12.9
13.0
13.1
14.4
14.7
14.9
15.3
15.5
15.6
15.7
15.8
16.0
16.1
16.2
16.4
16.5
16.7
16.9
17.3
17.5
18.0
18.1
19.1
19.7
22.5
23.7
24.4
24.9
25.2
25.5
25.6
25.9
26.3
26.8
27.4
27.7
27.8
28.3
28.8
29.9
32.4
32.6
32.7
33.2
ZMD101545
ZMD113923
ZMD29364
ZMD41022
ZMD57105 ZMD13909
ZMD64289
ZMD4678
ZMD42784
ZMD121528
ZMD85868
ZMD148750
ZMD4673
ZMD66036
ZMD12137 ZMD21655
ZMD154071
ZMD46631
ZMD86033
ZMD95937
ZMD88840 ZMD104473
ZMD94985 ZMD39455
ZMD90669 ZMD12761
ZMD66523
ZMD115859 ZMD106278
ZMD56182
ZMD33183 ZMD81898
ZMD5230
ZMD51566
ZMD150166 ZMD102298
ZMD25945 ZMD64624
ZMD14446
ZMD47550 ZMD10934
ZMD922
ZMD25047
ZMD65110 ZMD116992
ZMD123742
ZMD79920 ZMD15869
ZMD152578
ZMD153594
ZMD25148
ZMD120518
ZMD86875
ZMD39002
ZMD85196 ZMD149075
ZMD122285
ZMD25796
ZMD100438
ZMD23855
ZMD48719
ZMD57219
ZMD108664
ZMD7357
ZMD149826
ZMD151079
ZMD11419
ZMD32877 ZMD47474
ZMD90111
ZMD111831
ZMD7259 ZMD105716
ZMD106487 ZMD54244
ZMD70601
ZMD28889
ZMD102549
ZMD44246
ZMD130039
ZMD105038
ZMD123255
ZMD29158
ZMD61759 ZMD56592
ZMD38758 ZMD151138
ZMD106566
ZMD157527
ZMD62606
ZMD88985
ZMD128257 ZMD13908
ZMD29259
ZMD97181
ZMD40783
ZMD19894
ZMD14768
ZMD66790 ZMD101660
ZMD29916
ZMD103332
ZMD65124
ZMD32999
ZMD16506
ZMD13026
ZMD45770
ZMD56307
ZMD76359
ZMD49203
ZMD55466
ZMD20813
11 [2]
40.5 ZMD77407
41.5 ZMD75186
41.6 ZMD100791
41.7 ZMD91833
42.0 ZMD86472
42.5 ZMD51334
43.2 ZMD54218
44.0 ZMD68465
44.9 ZMD37761
45.4 ZMD36555
45.6 ZMD65819
45.9 ZMD25359
46.7 ZMD88219
47.7 ZMD31711
48.3 ZMD29612
48.4 ZMD16146
48.5 ZMD85723
48.8 ZMD106008
49.1 ZMD97209
49.4 ZMD25059
49.7 ZMD22932
50.1 ZMD107646
50.2 ZMD54075
50.7 ZMD55122
50.9 ZMD29726
51.9 ZMD53938
52.6 ZMD90237
54.0 ZMD57831
54.2 ZMD117120
54.9 ZMD24700
55.3 ZMD110651
55.9 ZMD98861
57.3 ZMD151072
57.8 ZMD20714 ZMD156149
58.7 ZMD58061
59.1 ZMD143931
59.3 ZMD77005 ZMD151903
ZMD34425
59.4 ZMD27008
61.5 ZMD89379
62.9 ZMD74914
64.3 ZMD48090

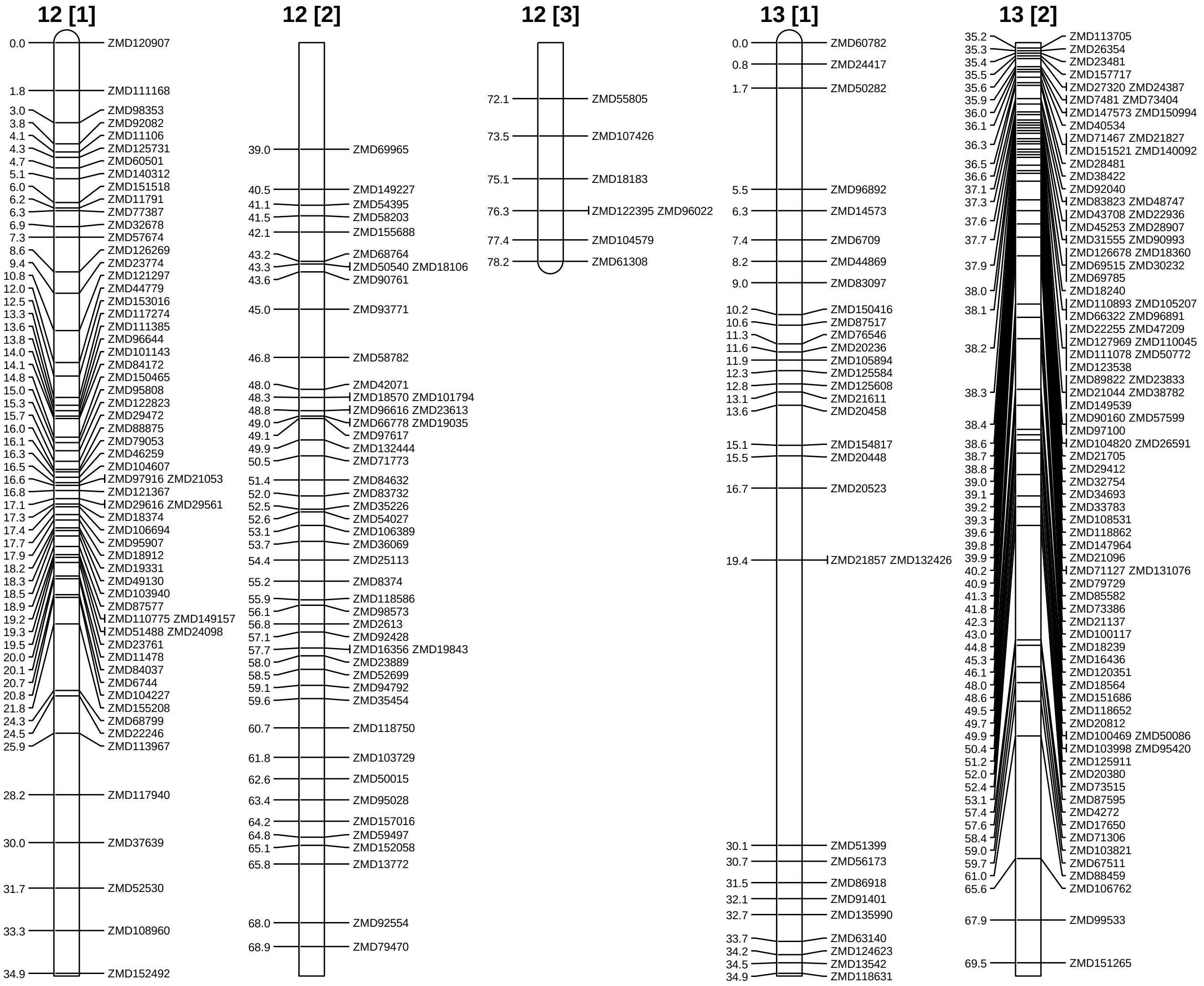
12 [1]
0.0 ZMD120907
1.8 ZMD111168
3.0 ZMD98353
3.8 ZMD92082
4.1 ZMD11106
4.3 ZMD125731
4.7 ZMD60501
5.1 ZMD140312
6.0 ZMD151518
6.2 ZMD11791
6.3 ZMD77387
6.9 ZMD32678
7.3 ZMD57674
8.6 ZMD126269
9.4 ZMD23774
10.8 ZMD121297
12.0 ZMD44779
12.5 ZMD153016
13.3 ZMD117274
13.6 ZMD111385
13.8 ZMD96644
14.0 ZMD101143
14.1 ZMD84172
14.8 ZMD150465
15.0 ZMD95808
15.3 ZMD122823
15.7 ZMD29472
16.0 ZMD88875
16.1 ZMD79053
16.3 ZMD46259
16.5 ZMD104607
16.6 ZMD97916 ZMD21053
16.8 ZMD121367
17.1 ZMD29616 ZMD29561
17.3 ZMD18374
17.4 ZMD106694
17.7 ZMD95907
17.9 ZMD18912
18.2 ZMD19331
18.3 ZMD49130
18.5 ZMD103940
18.9 ZMD87577
19.2 ZMD110775 ZMD149157
19.3 ZMD51488 ZMD24098
19.5 ZMD23761
20.0 ZMD11478
20.1 ZMD84037
20.7 ZMD6744
20.8 ZMD104227
21.8 ZMD155208
24.3 ZMD68799
24.5 ZMD22246
25.9 ZMD113967
28.2 ZMD117940
30.0 ZMD37639
31.7 ZMD52530
33.3 ZMD108960
34.9 ZMD152492
12 [2]
39.0 ZMD69965
40.5 ZMD149227
41.1 ZMD54395
41.5 ZMD58203
42.1 ZMD155688
43.2 ZMD68764
43.3 ZMD50540 ZMD18106
43.6 ZMD90761
45.0 ZMD93771
46.8 ZMD58782
48.0 ZMD42071
48.3 ZMD18570 ZMD101794
48.8 ZMD96616 ZMD23613
49.0 ZMD66778 ZMD19035
49.1 ZMD97617
49.9 ZMD132444
50.5 ZMD71773
51.4 ZMD84632
52.0 ZMD83732
52.5 ZMD35226
52.6 ZMD54027
53.1 ZMD106389
53.7 ZMD36069
54.4 ZMD25113
55.2 ZMD8374
55.9 ZMD118586
56.1 ZMD98573
56.8 ZMD2613
57.1 ZMD92428
57.7 ZMD16356 ZMD19843
58.0 ZMD23889
58.5 ZMD52699
59.1 ZMD94792
59.6 ZMD35454
60.7 ZMD118750
61.8 ZMD103729
62.6 ZMD50015
63.4 ZMD95028
64.2 ZMD157016
64.8 ZMD59497
65.1 ZMD152058
65.8 ZMD13772
68.0 ZMD92554
68.9 ZMD79470
12 [3]
72.1 ZMD55805
73.5 ZMD107426
75.1 ZMD18183
76.3 ZMD122395 ZMD96022
77.4 ZMD104579
78.2 ZMD61308
13 [1]
0.0 ZMD60782
0.8 ZMD24417
1.7 ZMD50282
5.5 ZMD96892
6.3 ZMD14573
7.4 ZMD6709
8.2 ZMD44869
9.0 ZMD83097
10.2 ZMD150416
10.6 ZMD87517
11.3 ZMD76546
11.6 ZMD20236
11.9 ZMD105894
12.3 ZMD125584
12.8 ZMD125608
13.1 ZMD21611
13.6 ZMD20458
15.1 ZMD154817
15.5 ZMD20448
16.7 ZMD20523
19.4 ZMD21857 ZMD132426
30.1 ZMD51399
30.7 ZMD56173
31.5 ZMD86918
32.1 ZMD91401
32.7 ZMD135990
33.7 ZMD63140
34.2 ZMD124623
34.5 ZMD13542
34.9 ZMD118631
13 [2]
35.2
35.3
35.4
35.5
35.6
35.9
36.0
36.1
36.3
36.5
36.6
37.1
37.3
37.6
37.7
37.9
38.0
38.1
38.2
38.3
38.4
38.6
38.7
38.8
39.0
39.1
39.2
39.3
39.6
39.8
39.9
40.2
40.9
41.3
41.8
42.3
43.0
44.8
45.3
46.1
48.0
48.6
49.5
49.7
49.9
50.4
51.2
52.0
52.4
53.1
57.4
57.6
58.4
59.0
59.7
61.0
65.6
67.9
69.5
ZMD113705
ZMD26354
ZMD23481
ZMD157717
ZMD27320 ZMD24387
ZMD7481 ZMD73404
ZMD147573 ZMD150994
ZMD40534
ZMD71467 ZMD21827
ZMD151521 ZMD140092
ZMD28481
ZMD38422
ZMD92040
ZMD83823 ZMD48747
ZMD43708 ZMD22936
ZMD45253 ZMD28907
ZMD31555 ZMD90993
ZMD126678 ZMD18360
ZMD69515 ZMD30232
ZMD69785
ZMD18240
ZMD110893 ZMD105207
ZMD66322 ZMD96891
ZMD22255 ZMD47209
ZMD127969 ZMD110045
ZMD111078 ZMD50772
ZMD123538
ZMD89822 ZMD23833
ZMD21044 ZMD38782
ZMD149539
ZMD90160 ZMD57599
ZMD97100
ZMD104820 ZMD26591
ZMD21705
ZMD29412
ZMD32754
ZMD34693
ZMD33783
ZMD108531
ZMD118862
ZMD147964
ZMD21096
ZMD71127 ZMD131076
ZMD79729
ZMD85582
ZMD73386
ZMD21137
ZMD100117
ZMD18239
ZMD16436
ZMD120351
ZMD18564
ZMD151686
ZMD118652
ZMD20812
ZMD100469 ZMD50086
ZMD103998 ZMD95420
ZMD125911
ZMD20380
ZMD73515
ZMD87595
ZMD4272
ZMD17650
ZMD71306
ZMD103821
ZMD67511
ZMD88459
ZMD106762
ZMD99533
ZMD151265

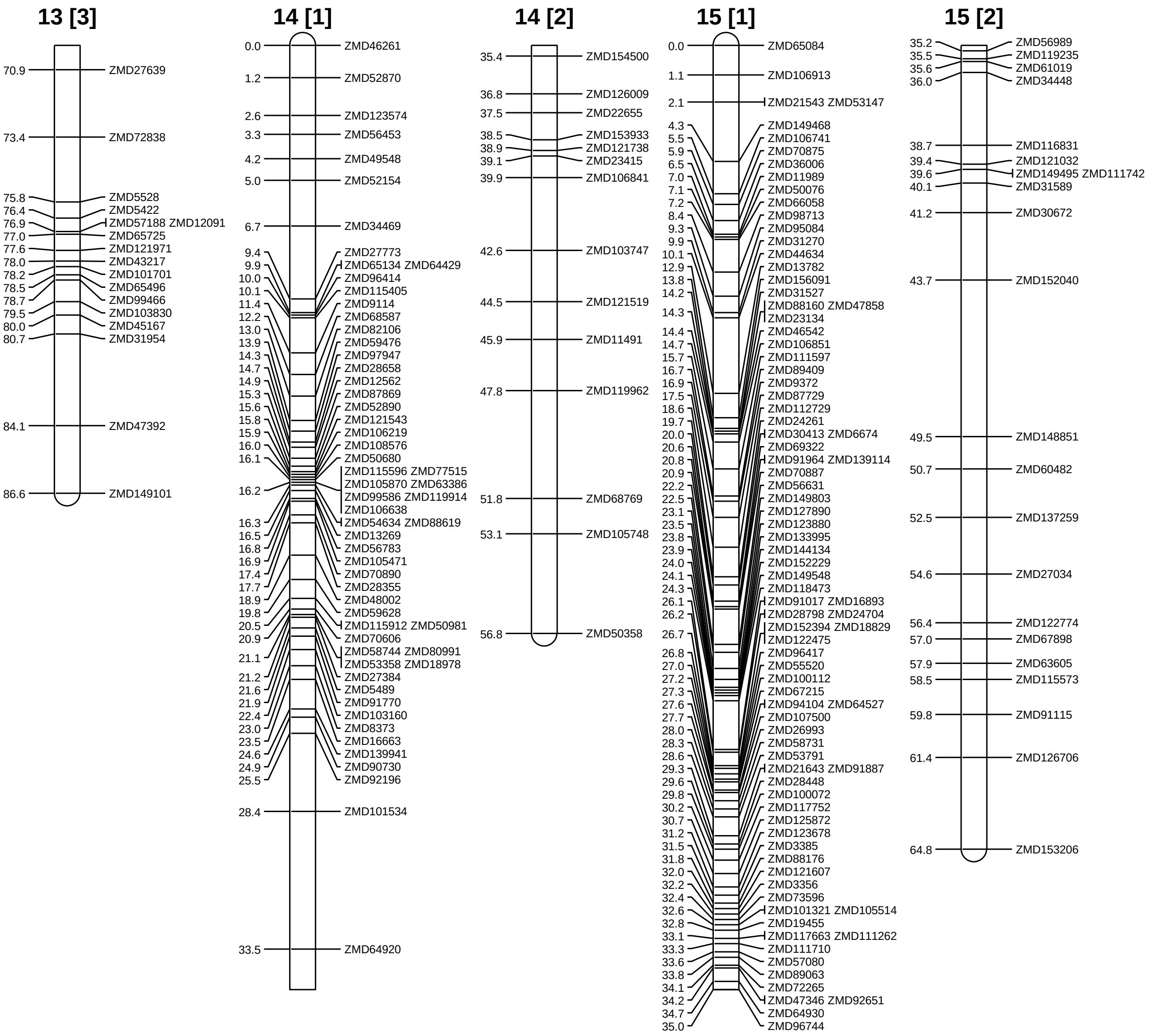
13 [3]
70.9 ZMD27639
73.4 ZMD72838
75.8 ZMD5528
76.4 ZMD5422
76.9 ZMD57188 ZMD12091
77.0 ZMD65725
77.6 ZMD121971
78.0 ZMD43217
78.2 ZMD101701
78.5 ZMD65496
78.7 ZMD99466
79.5 ZMD103830
80.0 ZMD45167
80.7 ZMD31954
84.1 ZMD47392
86.6 ZMD149101
14 [1]
0.0 ZMD46261
1.2 ZMD52870
2.6 ZMD123574
3.3 ZMD56453
4.2 ZMD49548
5.0 ZMD52154
6.7 ZMD34469
9.4 ZMD27773
9.9 ZMD65134 ZMD64429
10.0 ZMD96414
10.1 ZMD115405
11.4 ZMD9114
12.2 ZMD68587
13.0 ZMD82106
13.9 ZMD59476
14.3 ZMD97947
14.7 ZMD28658
14.9 ZMD12562
15.3 ZMD87869
15.6 ZMD52890
15.8 ZMD121543
15.9 ZMD106219
16.0 ZMD108576
16.1 ZMD50680
16.2 ZMD115596 ZMD77515 ZMD105870 ZMD63386 ZMD99586 ZMD119914 ZMD106638
16.3 ZMD54634 ZMD88619
16.5 ZMD13269
16.8 ZMD56783
16.9 ZMD105471
17.4 ZMD70890
17.7 ZMD28355
18.9 ZMD48002
19.8 ZMD59628
20.5 ZMD115912 ZMD50981
20.9 ZMD70606
21.1 ZMD58744 ZMD80991 ZMD53358 ZMD18978
21.2 ZMD27384
21.6 ZMD5489
21.9 ZMD91770
22.4 ZMD103160
23.0 ZMD8373
23.5 ZMD16663
24.6 ZMD139941
24.9 ZMD90730
25.5 ZMD92196
28.4 ZMD101534
33.5 ZMD64920
14 [2]
35.4 ZMD154500
36.8 ZMD126009
37.5 ZMD22655
38.5 ZMD153933
38.9 ZMD121738
39.1 ZMD23415
39.9 ZMD106841
42.6 ZMD103747
44.5 ZMD121519
45.9 ZMD11491
47.8 ZMD119962
51.8 ZMD68769
53.1 ZMD105748
56.8 ZMD50358
15 [1]
0.0 ZMD65084
1.1 ZMD106913
2.1 ZMD21543 ZMD53147
4.3 ZMD149468
5.5 ZMD106741
5.9 ZMD70875
6.5 ZMD36006
7.0 ZMD11989
7.1 ZMD50076
7.2 ZMD66058
8.4 ZMD98713
9.3 ZMD95084
9.9 ZMD31270
10.1 ZMD44634
12.9 ZMD13782
13.8 ZMD156091
14.2 ZMD31527
14.3 ZMD88160 ZMD47858 ZMD23134
14.4 ZMD46542
14.7 ZMD106851
15.7 ZMD111597
16.7 ZMD89409
16.9 ZMD9372
17.5 ZMD87729
18.6 ZMD112729
19.7 ZMD24261
20.0 ZMD30413 ZMD6674
20.6 ZMD69322
20.8 ZMD91964 ZMD139114
20.9 ZMD70887
22.2 ZMD56631
22.5 ZMD149803
23.1 ZMD127890
23.5 ZMD123880
23.8 ZMD133995
23.9 ZMD144134
24.0 ZMD152229
24.1 ZMD149548
24.3 ZMD118473
26.1 ZMD91017 ZMD16893
26.2 ZMD28798 ZMD24704
26.7 ZMD152394 ZMD18829 ZMD122475
26.8 ZMD96417
27.0 ZMD55520
27.2 ZMD100112
27.3 ZMD67215
27.6 ZMD94104 ZMD64527
27.7 ZMD107500
28.0 ZMD26993
28.3 ZMD58731
28.6 ZMD53791
29.3 ZMD21643 ZMD91887
29.6 ZMD28448
29.8 ZMD100072
30.2 ZMD117752
30.7 ZMD125872
31.2 ZMD123678
31.5 ZMD3385
31.8 ZMD88176
32.0 ZMD121607
32.2 ZMD3356
32.4 ZMD73596
32.6 ZMD101321 ZMD105514
32.8 ZMD19455
33.1 ZMD117663 ZMD111262
33.3 ZMD111710
33.6 ZMD57080
33.8 ZMD89063
34.1 ZMD72265
34.2 ZMD47346 ZMD92651
34.7 ZMD64930
35.0 ZMD96744
15 [2]
35.2 ZMD56989
35.5 ZMD119235
35.6 ZMD61019
36.0 ZMD34448
38.7 ZMD116831
39.4 ZMD121032
39.6 ZMD149495 ZMD111742
40.1 ZMD31589
41.2 ZMD30672
43.7 ZMD152040
49.5 ZMD148851
50.7 ZMD60482
52.5 ZMD137259
54.6 ZMD27034
56.4 ZMD122774
57.0 ZMD67898
57.9 ZMD63605
58.5 ZMD115573
59.8 ZMD91115
61.4 ZMD126706
64.8 ZMD153206

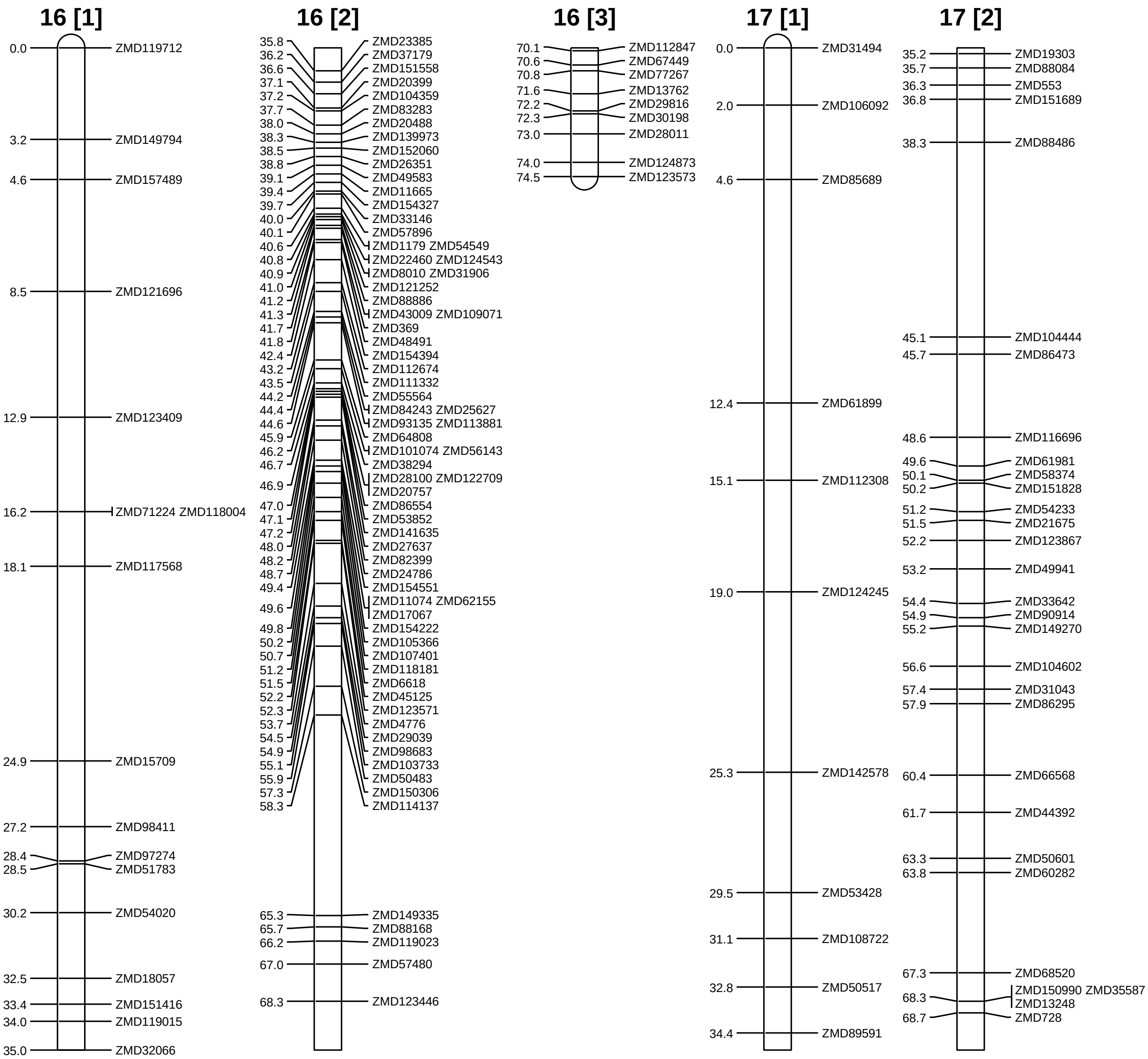
16 [1]
0.0 ZMD119712
3.2 ZMD149794
4.6 ZMD157489
8.5 ZMD121696
12.9 ZMD123409
16.2 ZMD71224 ZMD118004
18.1 ZMD117568
24.9 ZMD15709
27.2 ZMD98411
28.4 ZMD97274
28.5 ZMD51783
30.2 ZMD54020
32.5 ZMD18057
33.4 ZMD151416
34.0 ZMD119015
35.0 ZMD32066
16 [2]
35.8 ZMD23385
36.2 ZMD37179
36.6 ZMD151558
37.1 ZMD20399
37.2 ZMD104359
37.7 ZMD83283
38.0 ZMD20488
38.3 ZMD139973
38.5 ZMD152060
38.8 ZMD26351
39.1 ZMD49583
39.4 ZMD11665
39.7 ZMD154327
40.0 ZMD33146
40.1 ZMD57896
40.6 ZMD1179 ZMD54549
40.8 ZMD22460 ZMD124543
40.9 ZMD8010 ZMD31906
41.0 ZMD121252
41.2 ZMD88886
41.3 ZMD43009 ZMD109071
41.7 ZMD369
41.8 ZMD48491
42.4 ZMD154394
43.2 ZMD112674
43.5 ZMD111332
44.2 ZMD55564
44.4 ZMD84243 ZMD25627
44.6 ZMD93135 ZMD113881
45.9 ZMD64808
46.2 ZMD101074 ZMD56143
46.7 ZMD38294
46.9 ZMD28100 ZMD122709
ZMD20757
47.0 ZMD86554
47.1 ZMD53852
47.2 ZMD141635
48.0 ZMD27637
48.2 ZMD82399
48.7 ZMD24786
49.4 ZMD154551
49.6 ZMD11074 ZMD62155
ZMD17067
49.8 ZMD154222
50.2 ZMD105366
50.7 ZMD107401
51.2 ZMD118181
51.5 ZMD6618
52.2 ZMD45125
52.3 ZMD123571
53.7 ZMD4776
54.5 ZMD29039
54.9 ZMD98683
55.1 ZMD103733
55.9 ZMD50483
57.3 ZMD150306
58.3 ZMD114137
65.3 ZMD149335
65.7 ZMD88168
66.2 ZMD119023
67.0 ZMD57480
68.3 ZMD123446
16 [3]
70.1 ZMD112847
70.6 ZMD67449
70.8 ZMD77267
71.6 ZMD13762
72.2 ZMD29816
72.3 ZMD30198
73.0 ZMD28011
74.0 ZMD124873
74.5 ZMD123573
17 [1]
0.0 ZMD31494
2.0 ZMD106092
4.6 ZMD85689
12.4 ZMD61899
15.1 ZMD112308
19.0 ZMD124245
25.3 ZMD142578
29.5 ZMD53428
31.1 ZMD108722
32.8 ZMD50517
34.4 ZMD89591
17 [2]
35.2 ZMD19303
35.7 ZMD88084
36.3 ZMD553
36.8 ZMD151689
38.3 ZMD88486
45.1 ZMD104444
45.7 ZMD86473
48.6 ZMD116696
49.6 ZMD61981
50.1 ZMD58374
50.2 ZMD151828
51.2 ZMD54233
51.5 ZMD21675
52.2 ZMD123867
53.2 ZMD49941
54.4 ZMD33642
54.9 ZMD90914
55.2 ZMD149270
56.6 ZMD104602
57.4 ZMD31043
57.9 ZMD86295
60.4 ZMD66568
61.7 ZMD44392
63.3 ZMD50601
63.8 ZMD60282
67.3 ZMD68520
68.3 ZMD150990 ZMD35587
ZMD13248
68.7 ZMD728

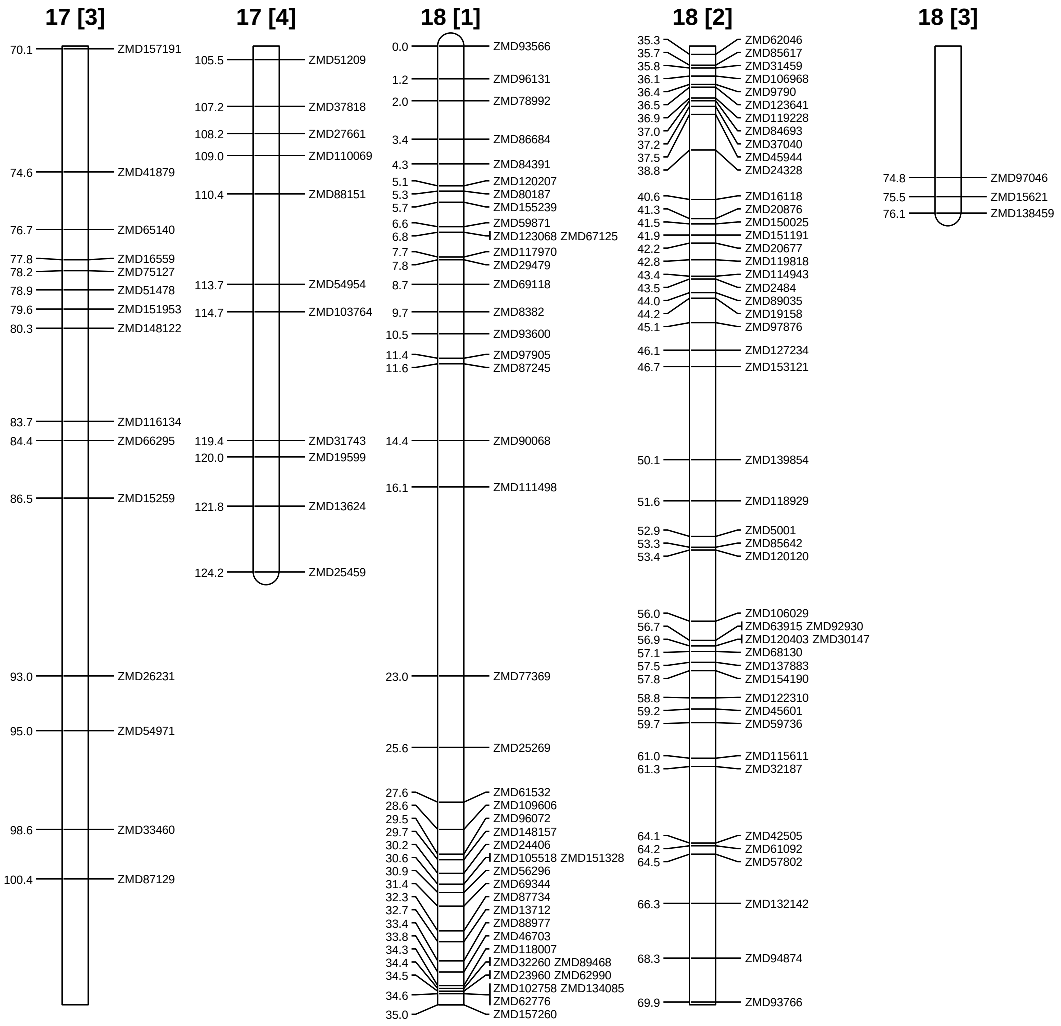

17 [3]
70.1 ZMD157191
74.6 ZMD41879
76.7 ZMD65140
77.8 ZMD16559
78.2 ZMD75127
78.9 ZMD51478
79.6 ZMD151953
80.3 ZMD148122
83.7 ZMD116134
84.4 ZMD66295
86.5 ZMD15259
93.0 ZMD26231
95.0 ZMD54971
98.6 ZMD33460
100.4 ZMD87129
17 [4]
105.5 ZMD51209
107.2 ZMD37818
108.2 ZMD27661
109.0 ZMD110069
110.4 ZMD88151
113.7 ZMD54954
114.7 ZMD103764
119.4 ZMD31743
120.0 ZMD19599
121.8 ZMD13624
124.2 ZMD25459
18 [1]
0.0 ZMD93566
1.2 ZMD96131
2.0 ZMD78992
3.4 ZMD86684
4.3 ZMD84391
5.1 ZMD120207
5.3 ZMD80187
5.7 ZMD155239
6.6 ZMD59871
6.8 ZMD123068 ZMD67125
7.7 ZMD117970
7.8 ZMD29479
8.7 ZMD69118
9.7 ZMD8382
10.5 ZMD93600
11.4 ZMD97905
11.6 ZMD87245
14.4 ZMD90068
16.1 ZMD111498
23.0 ZMD77369
25.6 ZMD25269
27.6 ZMD61532
28.6 ZMD109606
29.5 ZMD96072
29.7 ZMD148157
30.2 ZMD24406
30.6 ZMD105518 ZMD151328
30.9 ZMD56296
31.4 ZMD69344
32.3 ZMD87734
32.7 ZMD13712
33.4 ZMD88977
33.8 ZMD46703
34.3 ZMD118007
34.4 ZMD32260 ZMD89468
34.5 ZMD23960 ZMD62990
34.6 ZMD102758 ZMD134085
ZMD62776
35.0 ZMD157260
18 [2]
35.3 ZMD62046
35.7 ZMD85617
35.8 ZMD31459
36.1 ZMD106968
36.4 ZMD9790
36.5 ZMD123641
36.9 ZMD119228
37.0 ZMD84693
37.2 ZMD37040
37.5 ZMD45944
38.8 ZMD24328
40.6 ZMD16118
41.3 ZMD20876
41.5 ZMD150025
41.9 ZMD151191
42.2 ZMD20677
42.8 ZMD119818
43.4 ZMD114943
43.5 ZMD2484
44.0 ZMD89035
44.2 ZMD19158
45.1 ZMD97876
46.1 ZMD127234
46.7 ZMD153121
50.1 ZMD139854
51.6 ZMD118929
52.9 ZMD5001
53.3 ZMD85642
53.4 ZMD120120
56.0 ZMD106029
56.7 ZMD63915 ZMD92930
56.9 ZMD120403 ZMD30147
57.1 ZMD68130
57.5 ZMD137883
57.8 ZMD154190
58.8 ZMD122310
59.2 ZMD45601
59.7 ZMD59736
61.0 ZMD115611
61.3 ZMD32187
64.1 ZMD42505
64.2 ZMD61092
64.5 ZMD57802
66.3 ZMD132142
68.3 ZMD94874
69.9 ZMD93766
18 [3]
74.8 ZMD97046
75.5 ZMD15621
76.1 ZMD138459
19 [1]
0.0 ZMD30938
2.0 ZMD87338
2.2 ZMD68628
4.1 ZMD63991
5.2 ZMD23679
6.1 ZMD119380
6.3 ZMD91307
7.3 ZMD122284
8.9 ZMD64875
10.5 ZMD109942
12.0 ZMD88279
13.6 ZMD43418
14.0 ZMD149463
14.9 ZMD147611
15.0 ZMD32321
16.2 ZMD149784
16.6 ZMD22219
18.4 ZMD19677
18.9 ZMD100437
20.0 ZMD102806
23.0 ZMD150065
24.3 ZMD157520
25.6 ZMD155512
27.2 ZMD150406
32.0 ZMD129595
33.5 ZMD64137

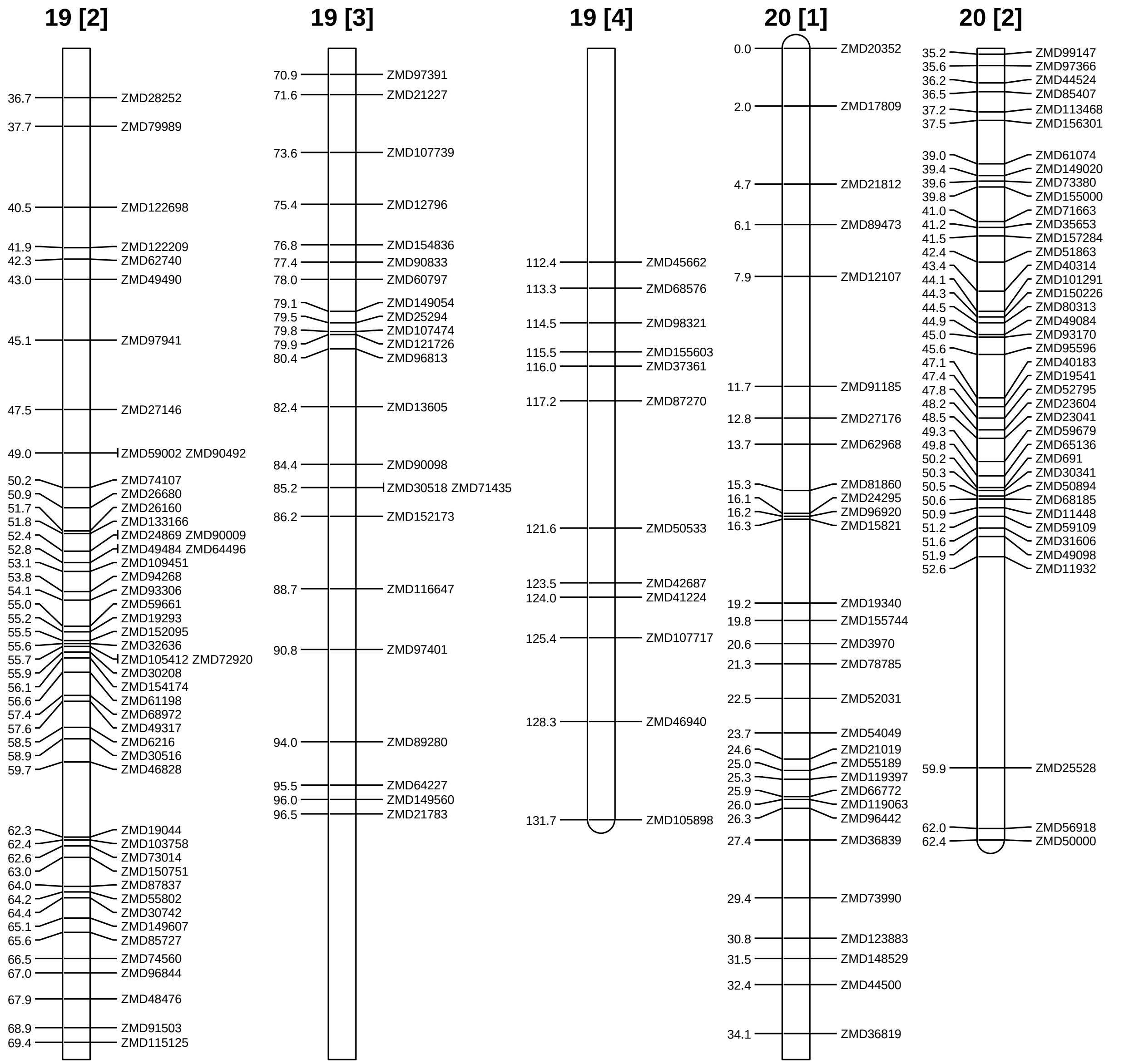

Figure S2 Detailed genetic map of Diamond

Numbers on top of the maps: linkage group (LG); numbers on the left side of each LG: genetic distance (cM); numbers on the right side of each LG: marker name.
