# Supplementary material for: Construction of high-resolution genetic maps of Zoysia matrella (L.) Merrill and applications to comparative genomic analysis and QTL mapping of resistance to fall armyworm
Source: BMC Genomics. 2016 Aug 8;17:562. doi: 10.1186/s12864-016-2969-7 (PMC4977732; doi:10.1186/s12864-016-2969-7)
Supplement: Additional file 5: Figure S3. — Detailed genetic map of Cavalier. Numbers on top of the maps: linkage group (LG); numbers on the left side of each LG: genetic distance (cM); numbers on the right side of each LG: marker name. (PDF 252 kb) [file 12864_2016_2969_MOESM5_ESM.pdf]

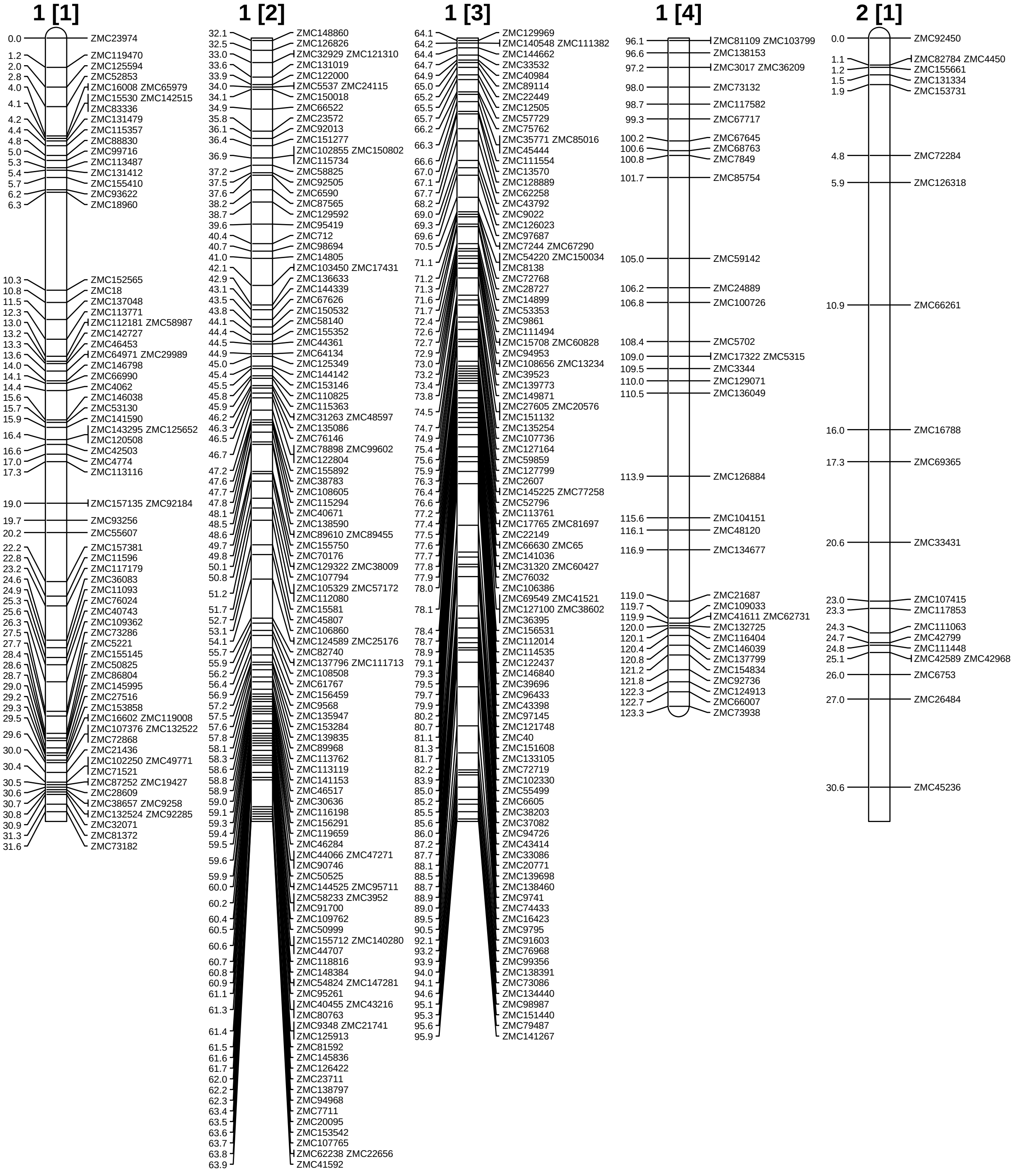
1 [1]
0.0
1.2
2.0
2.8
4.0
4.1
4.2
4.4
4.8
5.0
5.3
5.4
5.7
6.2
6.3
10.3
10.8
11.5
12.3
13.0
13.2
13.3
13.6
14.0
14.1
14.4
15.6
15.7
15.9
16.4
16.6
17.0
17.3
19.0
19.7
20.2
22.2
22.8
23.2
24.6
24.9
25.3
25.6
26.3
27.5
27.7
28.4
28.6
28.7
29.0
29.2
29.3
29.5
29.6
30.0
30.4
30.5
30.6
30.7
30.8
30.9
31.3
31.6
ZMC23974
ZMC119470
ZMC125594
ZMC52853
ZMC16008 ZMC65979
ZMC15530 ZMC142515
ZMC83336
ZMC131479
ZMC115357
ZMC88830
ZMC99716
ZMC113487
ZMC131412
ZMC155410
ZMC93622
ZMC18960
ZMC152565
ZMC18
ZMC137048
ZMC113771
ZMC112181 ZMC58987
ZMC142727
ZMC46453
ZMC64971 ZMC29989
ZMC146798
ZMC66990
ZMC4062
ZMC146038
ZMC53130
ZMC141590
ZMC143295 ZMC125652
ZMC120508
ZMC42503
ZMC4774
ZMC113116
ZMC157135 ZMC92184
ZMC93256
ZMC55607
ZMC157381
ZMC11596
ZMC117179
ZMC36083
ZMC11093
ZMC76024
ZMC40743
ZMC109362
ZMC73286
ZMC5221
ZMC155145
ZMC50825
ZMC86804
ZMC145995
ZMC27516
ZMC153858
ZMC16602 ZMC119008
ZMC107376 ZMC132522
ZMC72868
ZMC21436
ZMC102250 ZMC49771
ZMC71521
ZMC87252 ZMC19427
ZMC28609
ZMC38657 ZMC9258
ZMC132524 ZMC92285
ZMC32071
ZMC81372
ZMC73182
1 [2]
32.1
32.5
33.0
33.6
33.9
34.0
34.1
34.9
35.8
36.1
36.4
36.9
37.2
37.5
37.6
38.2
38.7
39.6
40.4
40.7
41.0
42.1
42.9
43.1
43.5
43.8
44.1
44.4
44.5
44.9
45.0
45.4
45.5
45.8
45.9
46.2
46.3
46.5
46.7
47.2
47.6
47.7
47.8
48.1
48.5
48.6
49.7
49.8
50.1
50.8
51.2
51.7
52.7
53.1
54.1
55.7
55.9
56.2
56.4
56.9
57.2
57.5
57.6
57.8
58.1
58.3
58.6
58.8
58.9
59.0
59.1
59.3
59.4
59.5
59.6
59.9
60.0
60.2
60.4
60.5
60.6
60.7
60.8
60.9
61.1
61.3
61.4
61.5
61.6
61.7
62.0
62.2
62.3
63.4
63.5
63.6
63.7
63.8
63.9
ZMC148860
ZMC126826
ZMC32929 ZMC121310
ZMC131019
ZMC122000
ZMC5537 ZMC24115
ZMC150018
ZMC66522
ZMC23572
ZMC92013
ZMC151277
ZMC102855 ZMC150802
ZMC115734
ZMC58825
ZMC92505
ZMC6590
ZMC87565
ZMC129592
ZMC95419
ZMC712
ZMC98694
ZMC14805
ZMC103450 ZMC17431
ZMC136633
ZMC144339
ZMC67626
ZMC150532
ZMC58140
ZMC155352
ZMC44361
ZMC64134
ZMC125349
ZMC144142
ZMC153146
ZMC110825
ZMC115363
ZMC31263 ZMC48597
ZMC135086
ZMC76146
ZMC78898 ZMC99602
ZMC122804
ZMC155892
ZMC38783
ZMC108605
ZMC115294
ZMC40671
ZMC138590
ZMC89610 ZMC89455
ZMC155750
ZMC70176
ZMC129322 ZMC38009
ZMC107794
ZMC105329 ZMC57172
ZMC112080
ZMC15581
ZMC45807
ZMC106860
ZMC124589 ZMC25176
ZMC82740
ZMC137796 ZMC111713
ZMC108508
ZMC61767
ZMC156459
ZMC9568
ZMC135947
ZMC153284
ZMC139835
ZMC89968
ZMC113762
ZMC113119
ZMC141153
ZMC46517
ZMC30636
ZMC116198
ZMC156291
ZMC119659
ZMC46284
ZMC44066 ZMC47271
ZMC90746
ZMC50525
ZMC144525 ZMC95711
ZMC58233 ZMC3952
ZMC91700
ZMC109762
ZMC50999
ZMC155712 ZMC140280
ZMC44707
ZMC118816
ZMC148384
ZMC54824 ZMC147281
ZMC95261
ZMC40455 ZMC43216
ZMC80763
ZMC9348 ZMC21741
ZMC125913
ZMC81592
ZMC145836
ZMC126422
ZMC23711
ZMC138797
ZMC94968
ZMC7711
ZMC20095
ZMC153542
ZMC107765
ZMC62238 ZMC22656
ZMC41592
1 [3]
64.1
64.2
64.4
64.7
64.9
65.0
65.2
65.5
65.7
66.2
66.3
66.6
67.0
67.1
67.7
68.2
69.0
69.3
69.6
70.5
71.1
71.2
71.3
71.6
71.7
72.4
72.6
72.7
72.9
73.0
73.2
73.4
73.8
74.5
74.7
74.9
75.4
75.6
75.9
76.3
76.4
76.6
77.2
77.4
77.5
77.6
77.7
77.8
77.9
78.0
78.1
78.4
78.7
78.9
79.1
79.3
79.5
79.7
79.9
80.2
80.7
81.1
81.3
81.7
82.2
83.9
85.0
85.2
85.5
85.6
86.0
87.2
87.7
88.1
88.5
88.7
88.9
89.0
89.5
90.5
92.1
93.2
93.9
94.0
94.1
94.6
95.1
95.3
95.6
95.9
ZMC129969
ZMC140548 ZMC111382
ZMC144662
ZMC33532
ZMC40984
ZMC89114
ZMC22449
ZMC12505
ZMC57729
ZMC75762
ZMC35771 ZMC85016
ZMC45444
ZMC111554
ZMC13570
ZMC128889
ZMC62258
ZMC43792
ZMC9022
ZMC126023
ZMC97687
ZMC7244 ZMC67290
ZMC54220 ZMC150034
ZMC8138
ZMC72768
ZMC28727
ZMC14899
ZMC53353
ZMC9861
ZMC111494
ZMC15708 ZMC60828
ZMC94953
ZMC108656 ZMC13234
ZMC39523
ZMC139773
ZMC149871
ZMC27605 ZMC20576
ZMC151132
ZMC135254
ZMC107736
ZMC127164
ZMC59859
ZMC127799
ZMC2607
ZMC145225 ZMC77258
ZMC52796
ZMC113761
ZMC17765 ZMC81697
ZMC22149
ZMC66630 ZMC65
ZMC141036
ZMC31320 ZMC60427
ZMC76032
ZMC106386
ZMC69549 ZMC41521
ZMC127100 ZMC38602
ZMC36395
ZMC156531
ZMC112014
ZMC114535
ZMC122437
ZMC146840
ZMC39696
ZMC96433
ZMC43398
ZMC97145
ZMC121748
ZMC40
ZMC151608
ZMC133105
ZMC72719
ZMC102330
ZMC55499
ZMC6605
ZMC38203
ZMC37082
ZMC94726
ZMC43414
ZMC33086
ZMC20771
ZMC139698
ZMC138460
ZMC9741
ZMC74433
ZMC16423
ZMC9795
ZMC91603
ZMC76968
ZMC99356
ZMC138391
ZMC73086
ZMC134440
ZMC98987
ZMC151440
ZMC79487
ZMC141267
1 [4]
96.1 ZMC81109 ZMC103799
96.6 ZMC138153
97.2 ZMC3017 ZMC36209
98.0 ZMC73132
98.7 ZMC117582
99.3 ZMC67717
100.2 ZMC67645
100.6 ZMC68763
100.8 ZMC7849
101.7 ZMC85754
105.0 ZMC59142
106.2 ZMC24889
106.8 ZMC100726
108.4 ZMC5702
109.0 ZMC17322 ZMC5315
109.5 ZMC3344
110.0 ZMC129071
110.5 ZMC136049
113.9 ZMC126884
115.6 ZMC104151
116.1 ZMC48120
116.9 ZMC134677
119.0 ZMC21687
119.7 ZMC109033
119.9 ZMC41611 ZMC62731
120.0 ZMC132725
120.1 ZMC116404
120.4 ZMC146039
120.8 ZMC137799
121.2 ZMC154834
121.8 ZMC92736
122.3 ZMC124913
122.7 ZMC66007
123.3 ZMC73938
2 [1]
0.0 ZMC92450
1.1 ZMC82784 ZMC4450
1.2 ZMC155661
1.5 ZMC131334
1.9 ZMC153731
4.8 ZMC72284
5.9 ZMC126318
10.9 ZMC66261
16.0 ZMC16788
17.3 ZMC69365
20.6 ZMC33431
23.0 ZMC107415
23.3 ZMC117853
24.3 ZMC111063
24.7 ZMC42799
24.8 ZMC111448
25.1 ZMC42589 ZMC42968
26.0 ZMC6753
27.0 ZMC26484
30.6 ZMC45236

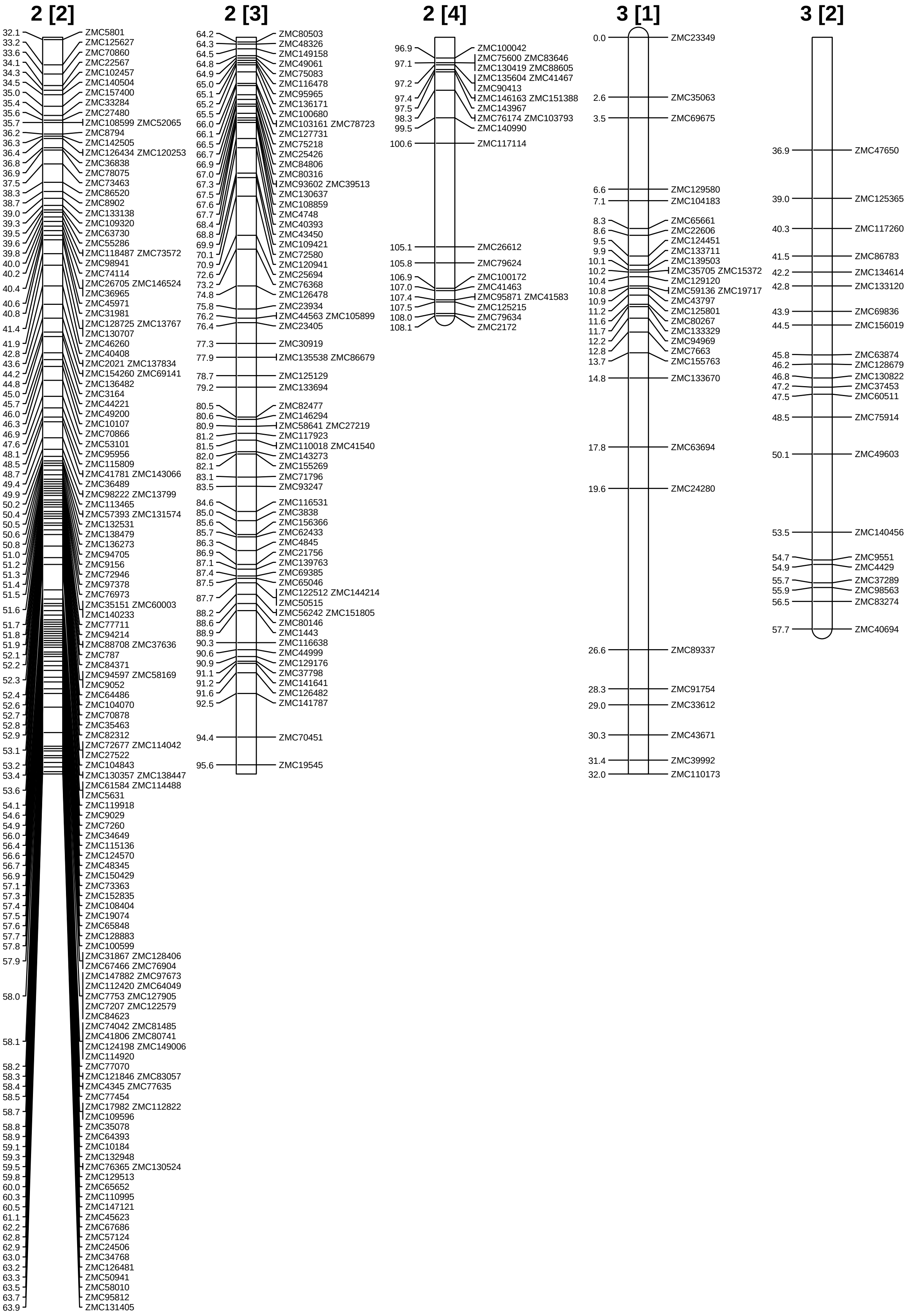
2 [2]
32.1
33.2
33.6
34.1
34.3
34.5
35.0
35.4
35.6
35.7
36.2
36.3
36.4
36.8
36.9
37.5
38.3
38.7
39.0
39.3
39.5
39.6
39.8
40.0
40.2
40.4
40.6
40.8
41.4
41.9
42.8
43.6
44.2
44.8
45.0
45.7
46.0
46.3
46.9
47.6
48.1
48.5
48.7
49.4
49.9
50.2
50.4
50.5
50.6
50.8
51.0
51.2
51.3
51.4
51.5
51.6
51.7
51.8
51.9
52.1
52.2
52.3
52.4
52.6
52.7
52.8
52.9
53.1
53.2
53.4
53.6
54.1
54.6
54.9
56.0
56.4
56.6
56.7
56.9
57.1
57.3
57.4
57.5
57.6
57.7
57.8
57.9
58.0
58.1
58.2
58.3
58.4
58.5
58.7
58.8
58.9
59.1
59.3
59.5
59.8
60.0
60.3
60.5
61.1
62.2
62.8
62.9
63.0
63.2
63.3
63.5
63.7
63.9
ZMC5801
ZMC125627
ZMC70860
ZMC22567
ZMC102457
ZMC140504
ZMC157400
ZMC33284
ZMC27480
ZMC108599 ZMC52065
ZMC8794
ZMC142505
ZMC126434 ZMC120253
ZMC36838
ZMC78075
ZMC73463
ZMC86520
ZMC8902
ZMC133138
ZMC109320
ZMC63730
ZMC55286
ZMC118487 ZMC73572
ZMC98941
ZMC74114
ZMC26705 ZMC146524
ZMC36965
ZMC45971
ZMC31981
ZMC128725 ZMC13767
ZMC130707
ZMC46260
ZMC40408
ZMC2021 ZMC137834
ZMC154260 ZMC69141
ZMC136482
ZMC3164
ZMC44221
ZMC49200
ZMC10107
ZMC70866
ZMC53101
ZMC95956
ZMC115809
ZMC41781 ZMC143066
ZMC36489
ZMC98222 ZMC13799
ZMC113465
ZMC57393 ZMC131574
ZMC132531
ZMC138479
ZMC136273
ZMC94705
ZMC9156
ZMC72946
ZMC97378
ZMC76973
ZMC35151 ZMC60003
ZMC140233
ZMC77711
ZMC94214
ZMC88708 ZMC37636
ZMC787
ZMC84371
ZMC94597 ZMC58169
ZMC9052
ZMC64486
ZMC104070
ZMC70878
ZMC35463
ZMC82312
ZMC72677 ZMC114042
ZMC27522
ZMC104843
ZMC130357 ZMC138447
ZMC61584 ZMC114488
ZMC5631
ZMC119918
ZMC9029
ZMC7260
ZMC34649
ZMC115136
ZMC124570
ZMC48345
ZMC150429
ZMC73363
ZMC152835
ZMC108404
ZMC19074
ZMC65848
ZMC128883
ZMC100599
ZMC31867 ZMC128406
ZMC67466 ZMC76904
ZMC147882 ZMC97673
ZMC112420 ZMC64049
ZMC7753 ZMC127905
ZMC7207 ZMC122579
ZMC84623
ZMC74042 ZMC81485
ZMC41806 ZMC80741
ZMC124198 ZMC149006
ZMC114920
ZMC77070
ZMC121846 ZMC83057
ZMC4345 ZMC77635
ZMC77454
ZMC17982 ZMC112822
ZMC109596
ZMC35078
ZMC64393
ZMC10184
ZMC132948
ZMC76365 ZMC130524
ZMC129513
ZMC65652
ZMC110995
ZMC147121
ZMC45623
ZMC67686
ZMC57124
ZMC24506
ZMC34768
ZMC126481
ZMC50941
ZMC58010
ZMC95812
ZMC131405
2 [3]
64.2
64.3
64.5
64.8
64.9
65.0
65.1
65.2
65.5
66.0
66.1
66.5
66.7
66.9
67.0
67.3
67.5
67.6
67.7
68.4
68.8
69.9
70.1
70.9
72.6
73.2
74.8
75.8
76.2
76.4
77.3
77.9
78.7
79.2
80.5
80.6
80.9
81.2
81.5
82.0
82.1
83.1
83.5
84.6
85.0
85.6
85.7
86.3
86.9
87.1
87.4
87.5
87.7
88.2
88.6
88.9
90.3
90.6
90.9
91.1
91.2
91.6
92.5
94.4
95.6
ZMC80503
ZMC48326
ZMC149158
ZMC49061
ZMC75083
ZMC116478
ZMC95965
ZMC136171
ZMC100680
ZMC103161 ZMC78723
ZMC127731
ZMC75218
ZMC25426
ZMC84806
ZMC80316
ZMC93602 ZMC39513
ZMC130637
ZMC108859
ZMC4748
ZMC40393
ZMC43450
ZMC109421
ZMC72580
ZMC120941
ZMC25694
ZMC76368
ZMC126478
ZMC23934
ZMC44563 ZMC105899
ZMC23405
ZMC30919
ZMC135538 ZMC86679
ZMC125129
ZMC133694
ZMC82477
ZMC146294
ZMC58641 ZMC27219
ZMC117923
ZMC110018 ZMC41540
ZMC143273
ZMC155269
ZMC71796
ZMC93247
ZMC116531
ZMC3838
ZMC156366
ZMC62433
ZMC4845
ZMC21756
ZMC139763
ZMC69385
ZMC65046
ZMC122512 ZMC144214
ZMC50515
ZMC56242 ZMC151805
ZMC80146
ZMC1443
ZMC116638
ZMC44999
ZMC129176
ZMC37798
ZMC141641
ZMC126482
ZMC141787
ZMC70451
ZMC19545
2 [4]
96.9
97.1
97.2
97.4
97.5
98.3
99.5
100.6
105.1
105.8
106.9
107.0
107.4
107.5
108.0
108.1
ZMC100042
ZMC75600 ZMC83646
ZMC130419 ZMC88605
ZMC135604 ZMC41467
ZMC90413
ZMC146163 ZMC151388
ZMC143967
ZMC76174 ZMC103793
ZMC140990
ZMC117114
ZMC26612
ZMC79624
ZMC100172
ZMC41463
ZMC95871 ZMC41583
ZMC125215
ZMC79634
ZMC2172
3 [1]
0.0 ZMC23349
2.6 ZMC35063
3.5 ZMC69675
6.6 ZMC129580
7.1 ZMC104183
8.3 ZMC65661
8.6 ZMC22606
9.5 ZMC124451
9.9 ZMC133711
10.1 ZMC139503
10.2 ZMC35705 ZMC15372
10.4 ZMC129120
10.8 ZMC59136 ZMC19717
10.9 ZMC43797
11.2 ZMC125801
11.6 ZMC80267
11.7 ZMC133329
12.2 ZMC94969
12.8 ZMC7663
13.7 ZMC155763
14.8 ZMC133670
17.8 ZMC63694
19.6 ZMC24280
26.6 ZMC89337
28.3 ZMC91754
29.0 ZMC33612
30.3 ZMC43671
31.4 ZMC39992
32.0 ZMC110173
3 [2]
36.9 ZMC47650
39.0 ZMC125365
40.3 ZMC117260
41.5 ZMC86783
42.2 ZMC134614
42.8 ZMC133120
43.9 ZMC69836
44.5 ZMC156019
45.8 ZMC63874
46.2 ZMC128679
46.8 ZMC130822
47.2 ZMC37453
47.5 ZMC60511
48.5 ZMC75914
50.1 ZMC49603
53.5 ZMC140456
54.7 ZMC9551
54.9 ZMC4429
55.7 ZMC37289
55.9 ZMC98563
56.5 ZMC83274
57.7 ZMC40694

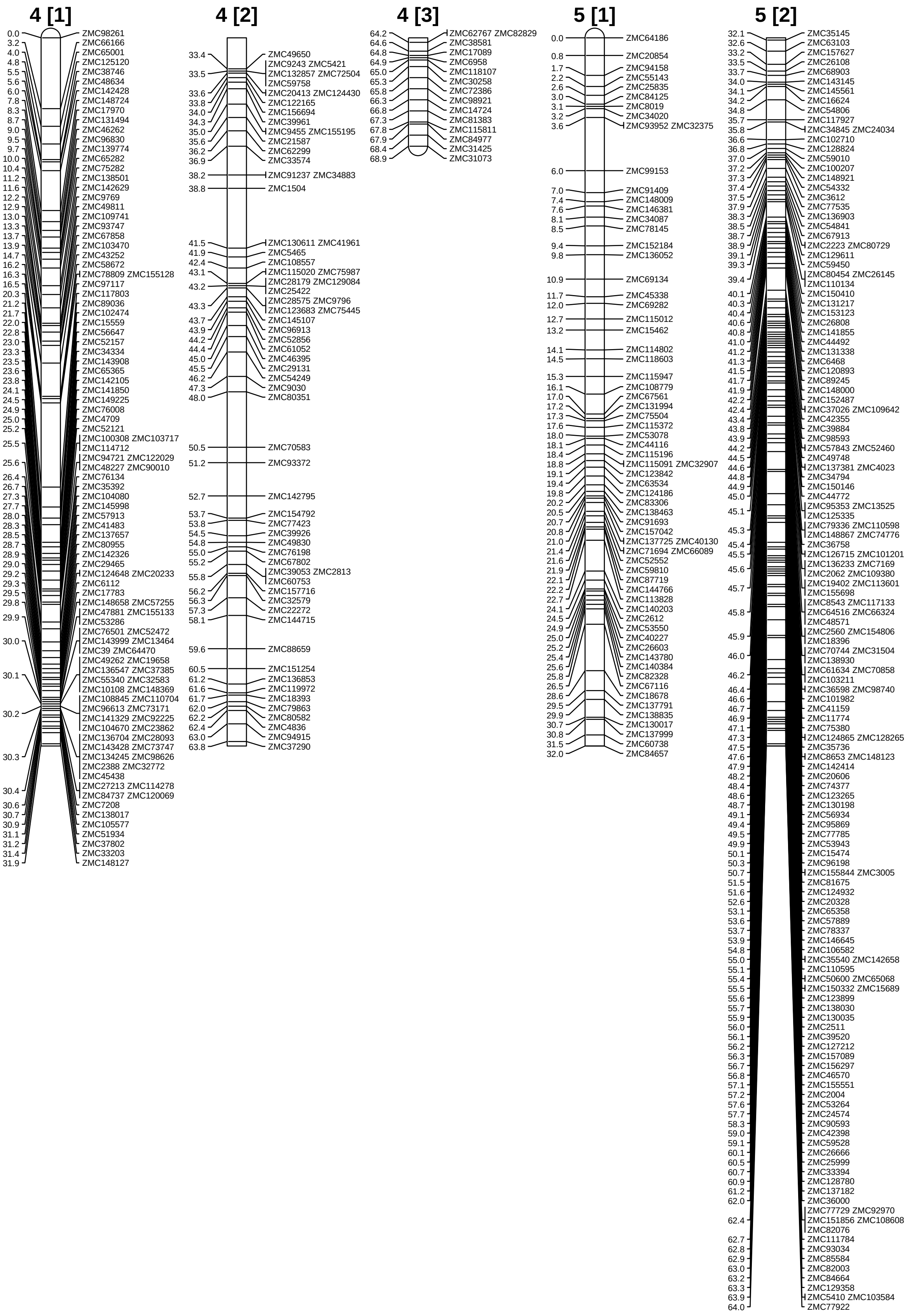
4 [1]
0.0
3.2
4.0
4.8
5.5
5.6
6.0
7.8
8.3
8.7
9.0
9.5
9.7
10.0
10.4
11.2
11.6
12.2
12.9
13.0
13.3
13.7
13.9
14.7
16.2
16.3
16.5
20.3
21.2
21.7
22.0
22.8
23.0
23.3
23.5
23.6
23.8
24.1
24.5
24.9
25.0
25.2
25.5
25.6
26.4
26.7
27.3
27.7
28.0
28.3
28.5
28.7
28.9
29.0
29.2
29.3
29.5
29.8
29.9
30.0
30.1
30.2
30.3
30.4
30.6
30.7
30.9
31.1
31.2
31.4
31.9
ZMC98261
ZMC66166
ZMC65001
ZMC125120
ZMC38746
ZMC48634
ZMC142428
ZMC148724
ZMC17970
ZMC131494
ZMC46262
ZMC96830
ZMC139774
ZMC65282
ZMC75282
ZMC138501
ZMC142629
ZMC9769
ZMC49811
ZMC109741
ZMC93747
ZMC67858
ZMC103470
ZMC43252
ZMC58672
ZMC78809 ZMC155128
ZMC97117
ZMC117803
ZMC89036
ZMC102474
ZMC15559
ZMC56647
ZMC52157
ZMC34334
ZMC143908
ZMC65365
ZMC142105
ZMC141850
ZMC149225
ZMC76008
ZMC4709
ZMC52121
ZMC100308 ZMC103717
ZMC114712
ZMC94721 ZMC122029
ZMC48227 ZMC90010
ZMC76134
ZMC35392
ZMC104080
ZMC145998
ZMC57913
ZMC41483
ZMC137657
ZMC80955
ZMC142326
ZMC29465
ZMC124648 ZMC20233
ZMC6112
ZMC17783
ZMC148658 ZMC57255
ZMC47881 ZMC155133
ZMC53286
ZMC76501 ZMC52472
ZMC143999 ZMC13464
ZMC39 ZMC64470
ZMC49262 ZMC19658
ZMC136547 ZMC37385
ZMC55340 ZMC32583
ZMC10108 ZMC148369
ZMC108845 ZMC110704
ZMC96613 ZMC73171
ZMC141329 ZMC92225
ZMC104670 ZMC23862
ZMC136704 ZMC28093
ZMC143428 ZMC73747
ZMC134245 ZMC98626
ZMC2388 ZMC32772
ZMC45438
ZMC27213 ZMC114278
ZMC84737 ZMC120069
ZMC7208
ZMC138017
ZMC105577
ZMC51934
ZMC37802
ZMC33203
ZMC148127
4 [2]
33.4
33.5
33.6
33.8
34.0
34.3
35.0
35.6
36.2
36.9
38.2
38.8
41.5
41.9
42.4
43.1
43.2
43.3
43.7
43.9
44.2
44.4
45.0
45.5
46.2
47.3
48.0
50.5
51.2
52.7
53.7
53.8
54.5
54.8
55.0
55.2
55.8
56.2
56.3
57.3
58.1
59.6
60.5
61.2
61.6
61.7
62.0
62.2
62.4
63.0
63.8
ZMC49650
ZMC9243 ZMC5421
ZMC132857 ZMC72504
ZMC59758
ZMC20413 ZMC124430
ZMC122165
ZMC156694
ZMC39961
ZMC9455 ZMC155195
ZMC21587
ZMC62299
ZMC33574
ZMC91237 ZMC34883
ZMC1504
ZMC130611 ZMC41961
ZMC5465
ZMC108557
ZMC115020 ZMC75987
ZMC28179 ZMC129084
ZMC25422
ZMC28575 ZMC9796
ZMC123683 ZMC75445
ZMC145107
ZMC96913
ZMC52856
ZMC61052
ZMC46395
ZMC29131
ZMC54249
ZMC9030
ZMC80351
ZMC70583
ZMC93372
ZMC142795
ZMC154792
ZMC77423
ZMC39926
ZMC49830
ZMC76198
ZMC67802
ZMC39053 ZMC2813
ZMC60753
ZMC157716
ZMC32579
ZMC22272
ZMC144715
ZMC88659
ZMC151254
ZMC136853
ZMC119972
ZMC18393
ZMC79863
ZMC80582
ZMC4836
ZMC94915
ZMC37290
4 [3]
64.2
64.6
64.8
64.9
65.0
65.3
65.8
66.3
66.8
67.3
67.8
67.9
68.4
68.9
ZMC62767 ZMC82829
ZMC38581
ZMC17089
ZMC6958
ZMC118107
ZMC30258
ZMC72386
ZMC98921
ZMC14724
ZMC81383
ZMC115811
ZMC84977
ZMC31425
ZMC31073
5 [1]
0.0
0.8
1.7
2.2
2.6
3.0
3.1
3.2
3.6
6.0
7.0
7.4
7.6
8.1
8.5
9.4
9.8
10.9
11.7
12.0
12.7
13.2
14.1
14.5
15.3
16.1
17.0
17.2
17.3
17.6
18.0
18.1
18.4
18.8
19.1
19.4
19.8
20.2
20.5
20.7
20.8
21.0
21.4
21.6
21.9
22.1
22.2
22.7
24.1
24.5
24.9
25.0
25.2
25.4
25.6
25.8
26.5
28.6
29.5
29.9
30.7
30.8
31.5
32.0
ZMC64186
ZMC20854
ZMC94158
ZMC55143
ZMC25835
ZMC84125
ZMC8019
ZMC34020
ZMC93952 ZMC32375
ZMC99153
ZMC91409
ZMC148009
ZMC146381
ZMC34087
ZMC78145
ZMC152184
ZMC136052
ZMC69134
ZMC45338
ZMC69282
ZMC115012
ZMC15462
ZMC114802
ZMC118603
ZMC115947
ZMC108779
ZMC67561
ZMC131994
ZMC75504
ZMC115372
ZMC53078
ZMC44116
ZMC115196
ZMC115091 ZMC32907
ZMC123842
ZMC63534
ZMC124186
ZMC83306
ZMC138463
ZMC91693
ZMC157042
ZMC137725 ZMC40130
ZMC71694 ZMC66089
ZMC52552
ZMC59810
ZMC87719
ZMC144766
ZMC113828
ZMC140203
ZMC2612
ZMC53550
ZMC40227
ZMC26603
ZMC143780
ZMC140384
ZMC82328
ZMC67116
ZMC18678
ZMC137791
ZMC138835
ZMC130017
ZMC137999
ZMC60738
ZMC84657
5 [2]
32.1
32.6
33.2
33.5
33.7
34.0
34.1
34.2
34.8
35.7
35.8
36.6
36.8
37.0
37.2
37.3
37.4
37.5
37.9
38.3
38.5
38.7
38.9
39.1
39.3
39.4
40.1
40.3
40.4
40.6
40.8
41.0
41.2
41.3
41.5
41.7
41.9
42.2
42.4
43.4
43.8
43.9
44.2
44.5
44.6
44.8
44.9
45.0
45.1
45.3
45.4
45.5
45.6
45.7
45.8
45.9
46.0
46.2
46.4
46.6
46.7
46.9
47.1
47.3
47.5
47.6
47.9
48.2
48.4
48.6
48.7
49.1
49.4
49.5
49.9
50.1
50.3
50.7
51.5
51.6
52.6
53.1
53.6
53.7
53.9
54.8
55.0
55.1
55.4
55.5
55.6
55.7
55.9
56.0
56.1
56.2
56.3
56.7
56.8
57.1
57.2
57.6
57.7
58.3
59.0
59.1
60.1
60.5
60.7
60.9
61.2
62.0
62.4
62.7
62.8
62.9
63.0
63.2
63.3
63.9
64.0
ZMC35145
ZMC63103
ZMC157627
ZMC26108
ZMC68903
ZMC143145
ZMC145561
ZMC16624
ZMC54806
ZMC117927
ZMC34845 ZMC24034
ZMC102710
ZMC128824
ZMC59010
ZMC100207
ZMC148921
ZMC54332
ZMC3612
ZMC77535
ZMC136903
ZMC54841
ZMC67913
ZMC2223 ZMC80729
ZMC129611
ZMC59450
ZMC80454 ZMC26145
ZMC110134
ZMC150410
ZMC131217
ZMC153123
ZMC26808
ZMC141855
ZMC44492
ZMC131338
ZMC6468
ZMC120893
ZMC89245
ZMC148000
ZMC152487
ZMC37026 ZMC109642
ZMC42355
ZMC39884
ZMC98593
ZMC57843 ZMC52460
ZMC49748
ZMC137381 ZMC4023
ZMC34794
ZMC150146
ZMC44772
ZMC95353 ZMC13525
ZMC125335
ZMC79336 ZMC110598
ZMC148867 ZMC74776
ZMC36758
ZMC126715 ZMC101201
ZMC136233 ZMC7169
ZMC2062 ZMC109380
ZMC19402 ZMC113601
ZMC155698
ZMC8543 ZMC117133
ZMC64516 ZMC66324
ZMC48571
ZMC2560 ZMC154806
ZMC18396
ZMC70744 ZMC31504
ZMC138930
ZMC61634 ZMC70858
ZMC103211
ZMC36598 ZMC98740
ZMC101982
ZMC41159
ZMC11774
ZMC75380
ZMC124865 ZMC128265
ZMC35736
ZMC8653 ZMC148123
ZMC142414
ZMC20606
ZMC74377
ZMC123265
ZMC130198
ZMC56934
ZMC95869
ZMC77785
ZMC53943
ZMC15474
ZMC96198
ZMC155844 ZMC3005
ZMC81675
ZMC124932
ZMC20328
ZMC65358
ZMC57889
ZMC78337
ZMC146645
ZMC106582
ZMC35540 ZMC142658
ZMC110595
ZMC50600 ZMC65068
ZMC150332 ZMC15689
ZMC123899
ZMC138030
ZMC130035
ZMC2511
ZMC39520
ZMC127212
ZMC157089
ZMC156297
ZMC46570
ZMC155551
ZMC2004
ZMC53264
ZMC24574
ZMC90593
ZMC42398
ZMC59528
ZMC26666
ZMC25999
ZMC33394
ZMC128780
ZMC137182
ZMC36000
ZMC77729 ZMC92970
ZMC151856 ZMC108608
ZMC82076
ZMC111784
ZMC93034
ZMC85584
ZMC82003
ZMC84664
ZMC129358
ZMC5410 ZMC103584
ZMC77922

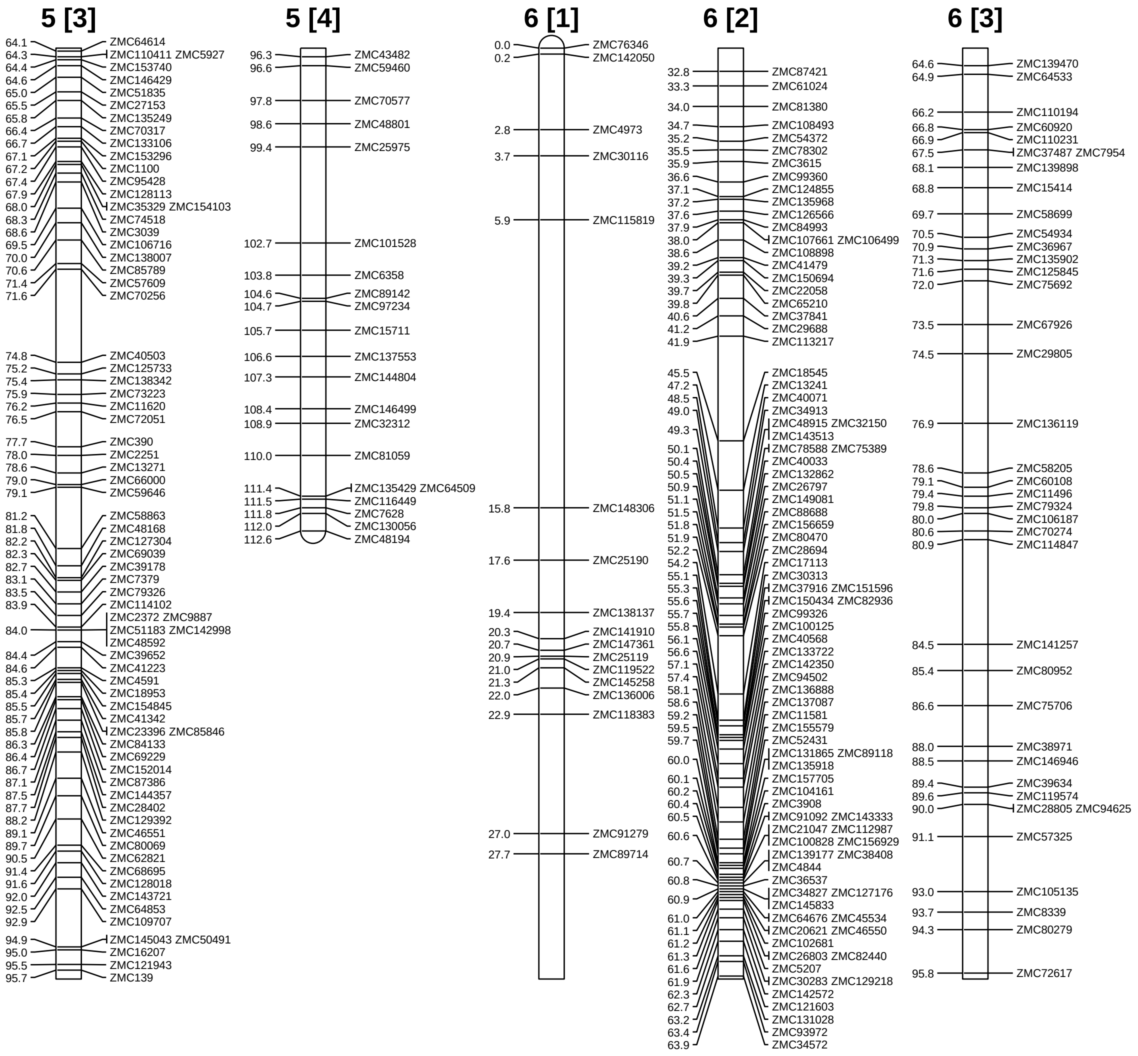
5 [3]
64.1 ZMC64614
64.3 ZMC110411 ZMC5927
64.4 ZMC153740
64.6 ZMC146429
65.0 ZMC51835
65.5 ZMC27153
65.8 ZMC135249
66.4 ZMC70317
66.7 ZMC133106
67.1 ZMC153296
67.2 ZMC1100
67.4 ZMC95428
67.9 ZMC128113
68.0 ZMC35329 ZMC154103
68.3 ZMC74518
68.6 ZMC3039
69.5 ZMC106716
70.0 ZMC138007
70.6 ZMC85789
71.4 ZMC57609
71.6 ZMC70256
74.8 ZMC40503
75.2 ZMC125733
75.4 ZMC138342
75.9 ZMC73223
76.2 ZMC11620
76.5 ZMC72051
77.7 ZMC390
78.0 ZMC2251
78.6 ZMC13271
79.0 ZMC66000
79.1 ZMC59646
81.2 ZMC58863
81.8 ZMC48168
82.2 ZMC127304
82.3 ZMC69039
82.7 ZMC39178
83.1 ZMC7379
83.5 ZMC79326
83.9 ZMC114102
ZMC2372 ZMC9887
84.0 ZMC51183 ZMC142998
ZMC48592
84.4 ZMC39652
84.6 ZMC41223
85.3 ZMC4591
85.4 ZMC18953
85.5 ZMC154845
85.7 ZMC41342
85.8 ZMC23396 ZMC85846
86.3 ZMC84133
86.4 ZMC69229
86.7 ZMC152014
87.1 ZMC87386
87.5 ZMC144357
87.7 ZMC28402
88.2 ZMC129392
89.1 ZMC46551
89.7 ZMC80069
90.5 ZMC62821
91.4 ZMC68695
91.6 ZMC128018
92.0 ZMC143721
92.5 ZMC64853
92.9 ZMC109707
94.9 ZMC145043 ZMC50491
95.0 ZMC16207
95.5 ZMC121943
95.7 ZMC139
5 [4]
96.3 ZMC43482
96.6 ZMC59460
97.8 ZMC70577
98.6 ZMC48801
99.4 ZMC25975
102.7 ZMC101528
103.8 ZMC6358
104.6 ZMC89142
104.7 ZMC97234
105.7 ZMC15711
106.6 ZMC137553
107.3 ZMC144804
108.4 ZMC146499
108.9 ZMC32312
110.0 ZMC81059
111.4 ZMC135429 ZMC64509
111.5 ZMC116449
111.8 ZMC7628
112.0 ZMC130056
112.6 ZMC48194
6 [1]
0.0 ZMC76346
0.2 ZMC142050
2.8 ZMC4973
3.7 ZMC30116
5.9 ZMC115819
15.8 ZMC148306
17.6 ZMC25190
19.4 ZMC138137
20.3 ZMC141910
20.7 ZMC147361
20.9 ZMC25119
21.0 ZMC119522
21.3 ZMC145258
22.0 ZMC136006
22.9 ZMC118383
27.0 ZMC91279
27.7 ZMC89714
6 [2]
32.8
33.3
34.0
34.7
35.2
35.5
35.9
36.6
37.1
37.2
37.6
37.9
38.0
38.6
39.2
39.3
39.7
39.8
40.6
41.2
41.9
45.5
47.2
48.5
49.0
49.3
50.1
50.4
50.5
50.9
51.1
51.5
51.8
51.9
52.2
54.2
55.1
55.3
55.6
55.7
55.8
56.1
56.6
57.1
57.4
58.1
58.6
59.2
59.5
59.7
60.0
60.1
60.2
60.4
60.5
60.6
60.7
60.8
60.9
61.0
61.1
61.2
61.3
61.6
61.9
62.3
62.7
63.2
63.4
63.9
ZMC87421
ZMC61024
ZMC81380
ZMC108493
ZMC54372
ZMC78302
ZMC3615
ZMC99360
ZMC124855
ZMC135968
ZMC126566
ZMC84993
ZMC107661 ZMC106499
ZMC108898
ZMC41479
ZMC150694
ZMC22058
ZMC65210
ZMC37841
ZMC29688
ZMC113217
ZMC18545
ZMC13241
ZMC40071
ZMC34913
ZMC48915 ZMC32150
ZMC143513
ZMC78588 ZMC75389
ZMC40033
ZMC132862
ZMC26797
ZMC149081
ZMC88688
ZMC156659
ZMC80470
ZMC28694
ZMC17113
ZMC30313
ZMC37916 ZMC151596
ZMC150434 ZMC82936
ZMC99326
ZMC100125
ZMC40568
ZMC133722
ZMC142350
ZMC94502
ZMC136888
ZMC137087
ZMC11581
ZMC155579
ZMC52431
ZMC131865 ZMC89118
ZMC135918
ZMC157705
ZMC104161
ZMC3908
ZMC91092 ZMC143333
ZMC21047 ZMC112987
ZMC100828 ZMC156929
ZMC139177 ZMC38408
ZMC4844
ZMC36537
ZMC34827 ZMC127176
ZMC145833
ZMC64676 ZMC45534
ZMC20621 ZMC46550
ZMC102681
ZMC26803 ZMC82440
ZMC5207
ZMC30283 ZMC129218
ZMC142572
ZMC121603
ZMC131028
ZMC93972
ZMC34572
6 [3]
64.6 ZMC139470
64.9 ZMC64533
66.2 ZMC110194
66.8 ZMC60920
66.9 ZMC110231
67.5 ZMC37487 ZMC7954
68.1 ZMC139898
68.8 ZMC15414
69.7 ZMC58699
70.5 ZMC54934
70.9 ZMC36967
71.3 ZMC135902
71.6 ZMC125845
72.0 ZMC75692
73.5 ZMC67926
74.5 ZMC29805
76.9 ZMC136119
78.6 ZMC58205
79.1 ZMC60108
79.4 ZMC11496
79.8 ZMC79324
80.0 ZMC106187
80.6 ZMC70274
80.9 ZMC114847
84.5 ZMC141257
85.4 ZMC80952
86.6 ZMC75706
88.0 ZMC38971
88.5 ZMC146946
89.4 ZMC39634
89.6 ZMC119574
90.0 ZMC28805 ZMC94625
91.1 ZMC57325
93.0 ZMC105135
93.7 ZMC8339
94.3 ZMC80279
95.8 ZMC72617

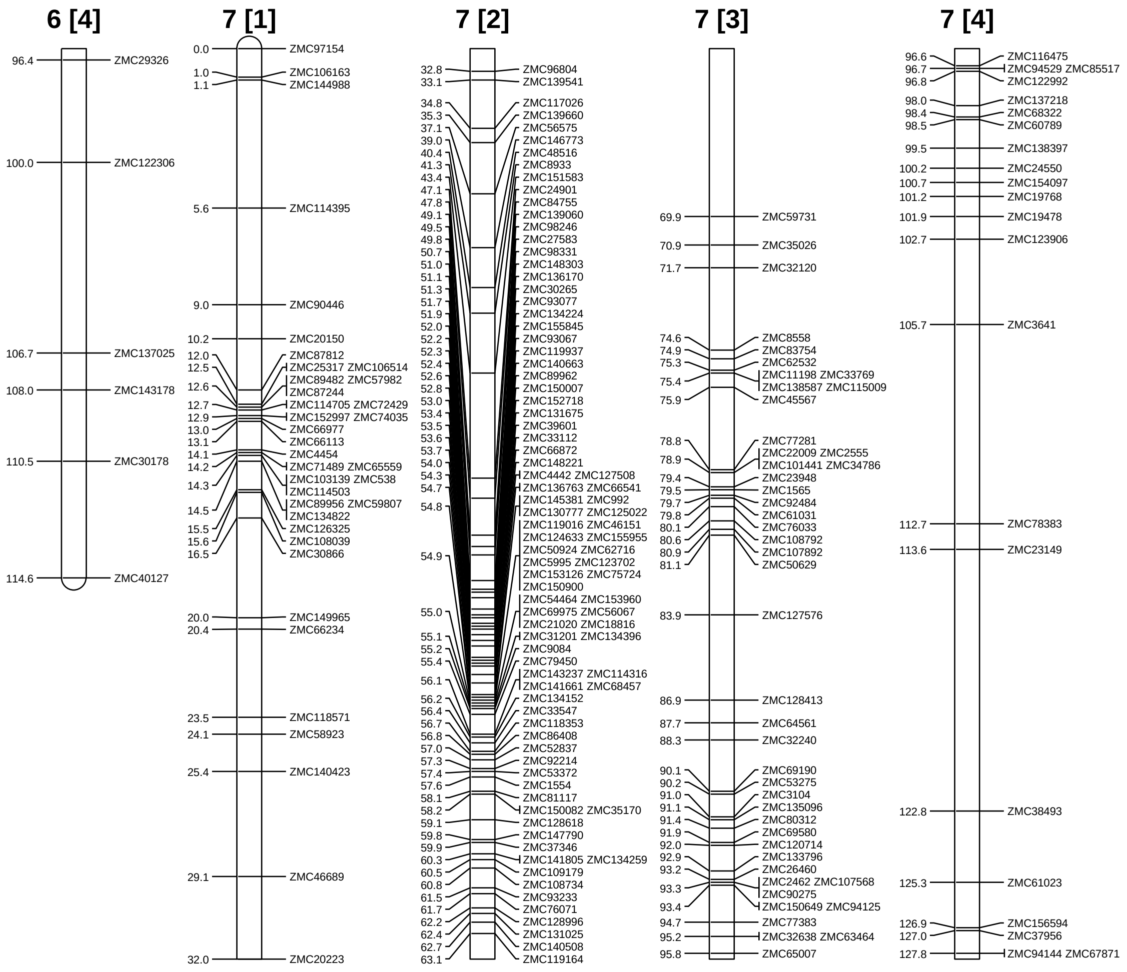
6 [4]
96.4 ZMC29326
100.0 ZMC122306
106.7 ZMC137025
108.0 ZMC143178
110.5 ZMC30178
114.6 ZMC40127
7 [1]
0.0 ZMC97154
1.0 ZMC106163
1.1 ZMC144988
5.6 ZMC114395
9.0 ZMC90446
10.2 ZMC20150
12.0 ZMC87812
12.5 ZMC25317 ZMC106514
12.6 ZMC89482 ZMC57982
ZMC87244
12.7 ZMC114705 ZMC72429
12.9 ZMC152997 ZMC74035
13.0 ZMC66977
13.1 ZMC66113
14.1 ZMC4454
14.2 ZMC71489 ZMC65559
14.3 ZMC103139 ZMC538
ZMC114503
14.5 ZMC89956 ZMC59807
ZMC134822
15.5 ZMC126325
15.6 ZMC108039
16.5 ZMC30866
20.0 ZMC149965
20.4 ZMC66234
23.5 ZMC118571
24.1 ZMC58923
25.4 ZMC140423
29.1 ZMC46689
32.0 ZMC20223
7 [2]
32.8 33.1 34.8 35.3 37.1 39.0 40.4 41.3 43.4 47.1 47.8 49.1 49.5 49.8 50.7 51.0 51.1 51.3 51.7 51.9 52.0 52.2 52.3 52.4 52.6 52.8 53.0 53.4 53.5 53.6 53.7 54.0 54.3 54.7 54.8 54.9 55.0 55.1 55.2 55.4 56.1 56.2 56.4 56.7 56.8 57.0 57.3 57.4 57.6 58.1 58.2 59.1 59.8 59.9 60.3 60.5 60.8 61.5 61.7 62.2 62.4 62.7 63.1
ZMC96804
ZMC139541
ZMC117026
ZMC139660
ZMC56575
ZMC146773
ZMC48516
ZMC8933
ZMC151583
ZMC24901
ZMC84755
ZMC139060
ZMC98246
ZMC27583
ZMC98331
ZMC148303
ZMC136170
ZMC30265
ZMC93077
ZMC134224
ZMC155845
ZMC93067
ZMC119937
ZMC140663
ZMC89962
ZMC150007
ZMC152718
ZMC131675
ZMC39601
ZMC33112
ZMC66872
ZMC148221
ZMC4442 ZMC127508
ZMC136763 ZMC66541
ZMC145381 ZMC992
ZMC130777 ZMC125022
ZMC119016 ZMC46151
ZMC124633 ZMC155955
ZMC50924 ZMC62716
ZMC5995 ZMC123702
ZMC153126 ZMC75724
ZMC150900
ZMC54464 ZMC153960
ZMC69975 ZMC56067
ZMC21020 ZMC18816
ZMC31201 ZMC134396
ZMC9084
ZMC79450
ZMC143237 ZMC114316
ZMC141661 ZMC68457
ZMC134152
ZMC33547
ZMC118353
ZMC86408
ZMC52837
ZMC92214
ZMC53372
ZMC1554
ZMC81117
ZMC150082 ZMC35170
ZMC128618
ZMC147790
ZMC37346
ZMC141805 ZMC134259
ZMC109179
ZMC108734
ZMC93233
ZMC76071
ZMC128996
ZMC131025
ZMC140508
ZMC119164
7 [3]
69.9 ZMC59731
70.9 ZMC35026
71.7 ZMC32120
74.6 ZMC8558
74.9 ZMC83754
75.3 ZMC62532
75.4 ZMC11198 ZMC33769
ZMC138587 ZMC115009
75.9 ZMC45567
78.8 ZMC77281
78.9 ZMC22009 ZMC2555
ZMC101441 ZMC34786
79.4 ZMC23948
79.5 ZMC1565
79.7 ZMC92484
79.8 ZMC61031
80.1 ZMC76033
80.6 ZMC108792
80.9 ZMC107892
81.1 ZMC50629
83.9 ZMC127576
86.9 ZMC128413
87.7 ZMC64561
88.3 ZMC32240
90.1 ZMC69190
90.2 ZMC53275
91.0 ZMC3104
91.1 ZMC135096
91.4 ZMC80312
91.9 ZMC69580
92.0 ZMC120714
92.9 ZMC133796
93.2 ZMC26460
93.3 ZMC2462 ZMC107568
ZMC90275
93.4 ZMC150649 ZMC94125
94.7 ZMC77383
95.2 ZMC32638 ZMC63464
95.8 ZMC65007
7 [4]
96.6 ZMC116475
96.7 ZMC94529 ZMC85517
96.8 ZMC122992
98.0 ZMC137218
98.4 ZMC68322
98.5 ZMC60789
99.5 ZMC138397
100.2 ZMC24550
100.7 ZMC154097
101.2 ZMC19768
101.9 ZMC19478
102.7 ZMC123906
105.7 ZMC3641
112.7 ZMC78383
113.6 ZMC23149
122.8 ZMC38493
125.3 ZMC61023
126.9 ZMC156594
127.0 ZMC37956
127.8 ZMC94144 ZMC67871

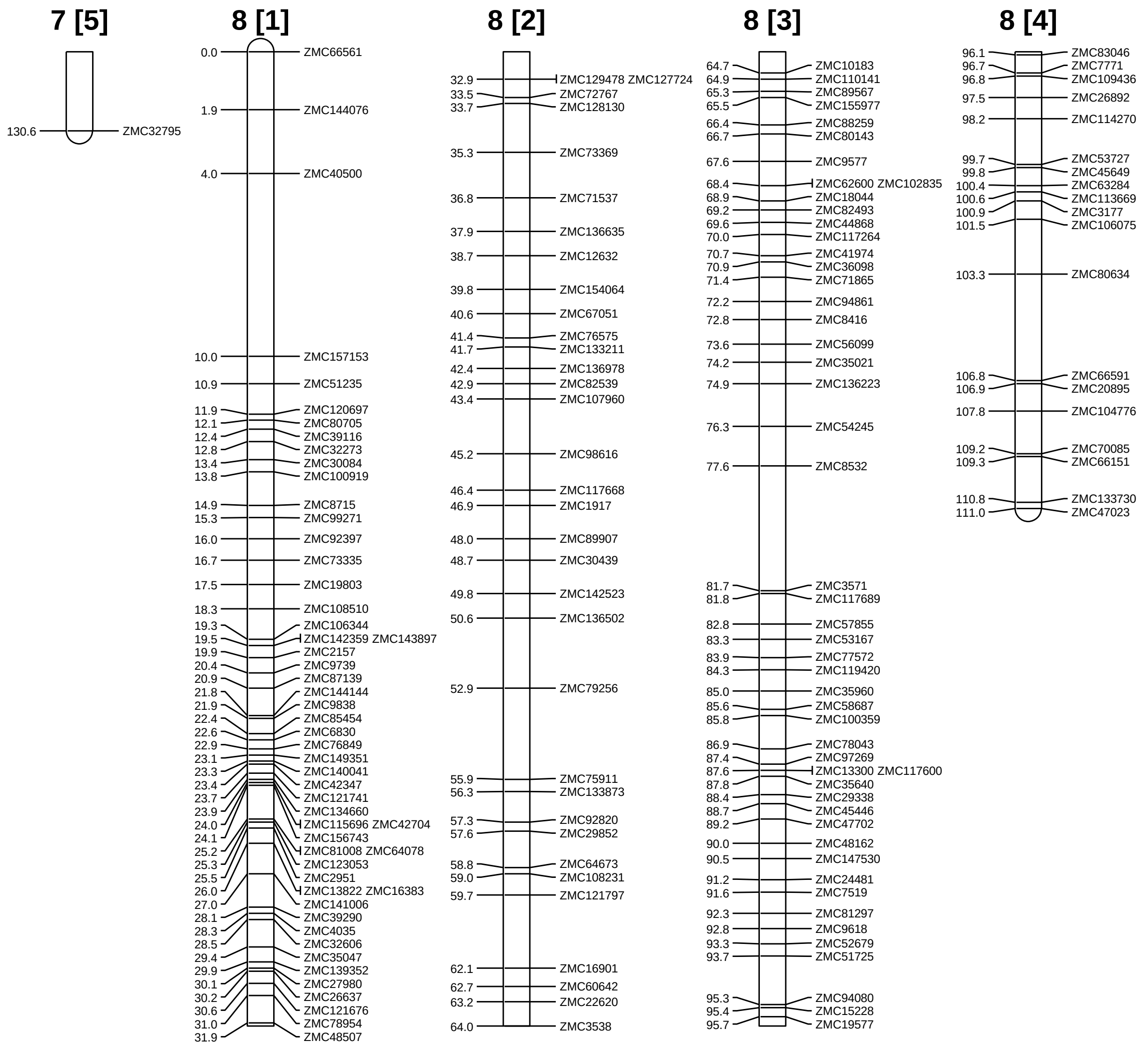
7 [5]
130.6 ZMC32795
8 [1]
0.0 ZMC66561
1.9 ZMC144076
4.0 ZMC40500
10.0 ZMC157153
10.9 ZMC51235
11.9 ZMC120697
12.1 ZMC80705
12.4 ZMC39116
12.8 ZMC32273
13.4 ZMC30084
13.8 ZMC100919
14.9 ZMC8715
15.3 ZMC99271
16.0 ZMC92397
16.7 ZMC73335
17.5 ZMC19803
18.3 ZMC108510
19.3 ZMC106344
19.5 ZMC142359 ZMC143897
19.9 ZMC2157
20.4 ZMC9739
20.9 ZMC87139
21.8 ZMC144144
21.9 ZMC9838
22.4 ZMC85454
22.6 ZMC6830
22.9 ZMC76849
23.1 ZMC149351
23.3 ZMC140041
23.4 ZMC42347
23.7 ZMC121741
23.9 ZMC134660
24.0 ZMC115696 ZMC42704
24.1 ZMC156743
25.2 ZMC81008 ZMC64078
25.3 ZMC123053
25.5 ZMC2951
26.0 ZMC13822 ZMC16383
27.0 ZMC141006
28.1 ZMC39290
28.3 ZMC4035
28.5 ZMC32606
29.4 ZMC35047
29.9 ZMC139352
30.1 ZMC27980
30.2 ZMC26637
30.6 ZMC121676
31.0 ZMC78954
31.9 ZMC48507
8 [2]
32.9 ZMC129478 ZMC127724
33.5 ZMC72767
33.7 ZMC128130
35.3 ZMC73369
36.8 ZMC71537
37.9 ZMC136635
38.7 ZMC12632
39.8 ZMC154064
40.6 ZMC67051
41.4 ZMC76575
41.7 ZMC133211
42.4 ZMC136978
42.9 ZMC82539
43.4 ZMC107960
45.2 ZMC98616
46.4 ZMC117668
46.9 ZMC1917
48.0 ZMC89907
48.7 ZMC30439
49.8 ZMC142523
50.6 ZMC136502
52.9 ZMC79256
55.9 ZMC75911
56.3 ZMC133873
57.3 ZMC92820
57.6 ZMC29852
58.8 ZMC64673
59.0 ZMC108231
59.7 ZMC121797
62.1 ZMC16901
62.7 ZMC60642
63.2 ZMC22620
64.0 ZMC3538
8 [3]
64.7 ZMC10183
64.9 ZMC110141
65.3 ZMC89567
65.5 ZMC155977
66.4 ZMC88259
66.7 ZMC80143
67.6 ZMC9577
68.4 ZMC62600 ZMC102835
68.9 ZMC18044
69.2 ZMC82493
69.6 ZMC44868
70.0 ZMC117264
70.7 ZMC41974
70.9 ZMC36098
71.4 ZMC71865
72.2 ZMC94861
72.8 ZMC8416
73.6 ZMC56099
74.2 ZMC35021
74.9 ZMC136223
76.3 ZMC54245
77.6 ZMC8532
81.7 ZMC3571
81.8 ZMC117689
82.8 ZMC57855
83.3 ZMC53167
83.9 ZMC77572
84.3 ZMC119420
85.0 ZMC35960
85.6 ZMC58687
85.8 ZMC100359
86.9 ZMC78043
87.4 ZMC97269
87.6 ZMC13300 ZMC117600
87.8 ZMC35640
88.4 ZMC29338
88.7 ZMC45446
89.2 ZMC47702
90.0 ZMC48162
90.5 ZMC147530
91.2 ZMC24481
91.6 ZMC7519
92.3 ZMC81297
92.8 ZMC9618
93.3 ZMC52679
93.7 ZMC51725
95.3 ZMC94080
95.4 ZMC15228
95.7 ZMC19577
8 [4]
96.1 ZMC83046
96.7 ZMC7771
96.8 ZMC109436
97.5 ZMC26892
98.2 ZMC114270
99.7 ZMC53727
99.8 ZMC45649
100.4 ZMC63284
100.6 ZMC113669
100.9 ZMC3177
101.5 ZMC106075
103.3 ZMC80634
106.8 ZMC66591
106.9 ZMC20895
107.8 ZMC104776
109.2 ZMC70085
109.3 ZMC66151
110.8 ZMC133730
111.0 ZMC47023

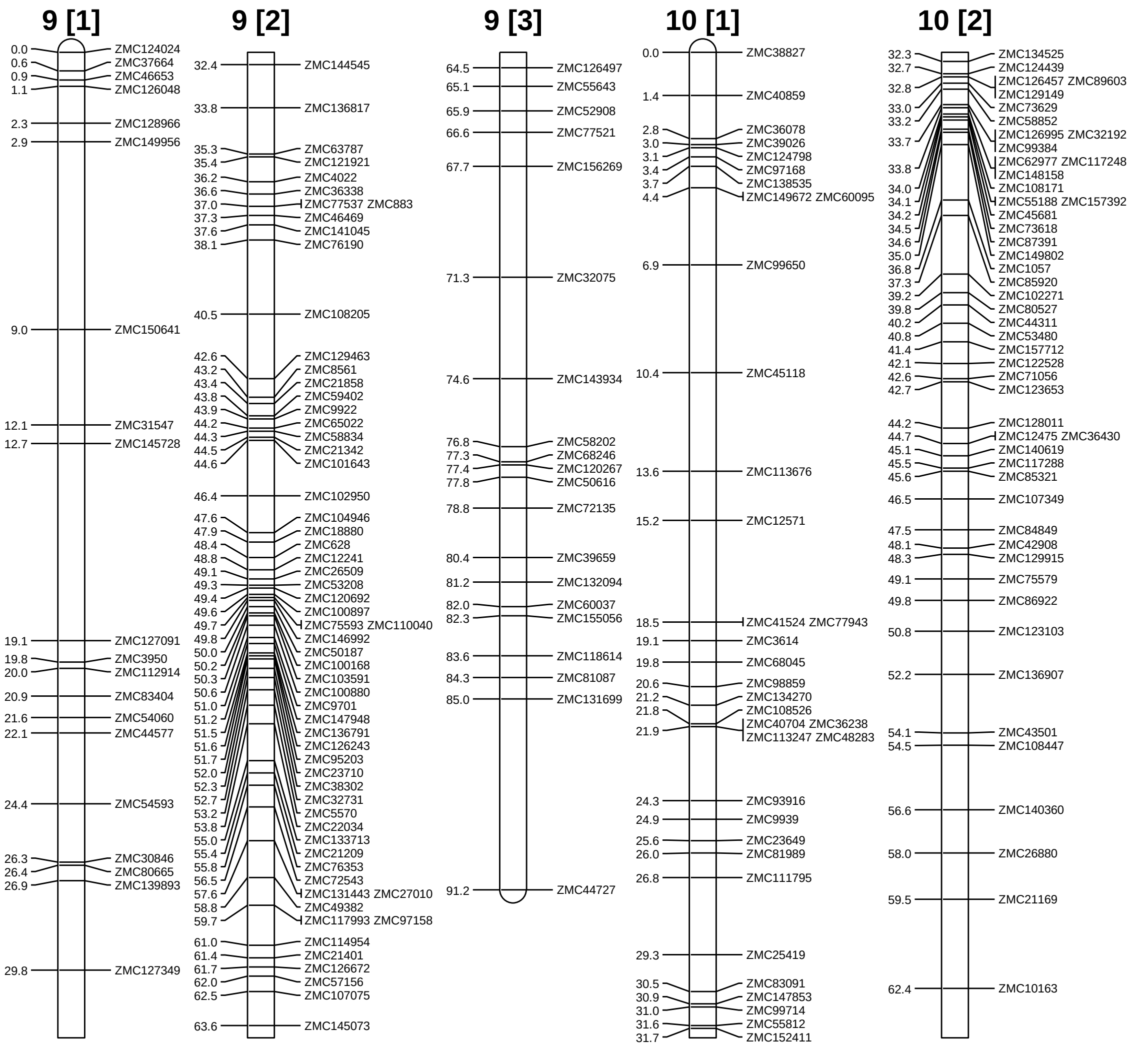
9 [1]
0.0 ZMC124024
0.6 ZMC37664
0.9 ZMC46653
1.1 ZMC126048
2.3 ZMC128966
2.9 ZMC149956
9.0 ZMC150641
12.1 ZMC31547
12.7 ZMC145728
19.1 ZMC127091
19.8 ZMC3950
20.0 ZMC112914
20.9 ZMC83404
21.6 ZMC54060
22.1 ZMC44577
24.4 ZMC54593
26.3 ZMC30846
26.4 ZMC80665
26.9 ZMC139893
29.8 ZMC127349
9 [2]
32.4 ZMC144545
33.8 ZMC136817
35.3 ZMC63787
35.4 ZMC121921
36.2 ZMC4022
36.6 ZMC36338
37.0 ZMC77537 ZMC883
37.3 ZMC46469
37.6 ZMC141045
38.1 ZMC76190
40.5 ZMC108205
42.6 ZMC129463
43.2 ZMC8561
43.4 ZMC21858
43.8 ZMC59402
43.9 ZMC9922
44.2 ZMC65022
44.3 ZMC58834
44.5 ZMC21342
44.6 ZMC101643
46.4 ZMC102950
47.6 ZMC104946
47.9 ZMC18880
48.4 ZMC628
48.8 ZMC12241
49.1 ZMC26509
49.3 ZMC53208
49.4 ZMC120692
49.6 ZMC100897
49.7 ZMC75593 ZMC110040
49.8 ZMC146992
50.0 ZMC50187
50.2 ZMC100168
50.3 ZMC103591
50.6 ZMC100880
51.0 ZMC9701
51.2 ZMC147948
51.5 ZMC136791
51.6 ZMC126243
51.7 ZMC95203
52.0 ZMC23710
52.3 ZMC38302
52.7 ZMC32731
53.2 ZMC5570
53.8 ZMC22034
55.0 ZMC133713
55.4 ZMC21209
55.8 ZMC76353
56.5 ZMC72543
57.6 ZMC131443 ZMC27010
58.8 ZMC49382
59.7 ZMC117993 ZMC97158
61.0 ZMC114954
61.4 ZMC21401
61.7 ZMC126672
62.0 ZMC57156
62.5 ZMC107075
63.6 ZMC145073
9 [3]
64.5 ZMC126497
65.1 ZMC55643
65.9 ZMC52908
66.6 ZMC77521
67.7 ZMC156269
71.3 ZMC32075
74.6 ZMC143934
76.8 ZMC58202
77.3 ZMC68246
77.4 ZMC120267
77.8 ZMC50616
78.8 ZMC72135
80.4 ZMC39659
81.2 ZMC132094
82.0 ZMC60037
82.3 ZMC155056
83.6 ZMC118614
84.3 ZMC81087
85.0 ZMC131699
91.2 ZMC44727
10 [1]
0.0 ZMC38827
1.4 ZMC40859
2.8 ZMC36078
3.0 ZMC39026
3.1 ZMC124798
3.4 ZMC97168
3.7 ZMC138535
4.4 ZMC149672 ZMC60095
6.9 ZMC99650
10.4 ZMC45118
13.6 ZMC113676
15.2 ZMC12571
18.5 ZMC41524 ZMC77943
19.1 ZMC3614
19.8 ZMC68045
20.6 ZMC98859
21.2 ZMC134270
21.8 ZMC108526
21.9 ZMC40704 ZMC36238 ZMC113247 ZMC48283
24.3 ZMC93916
24.9 ZMC9939
25.6 ZMC23649
26.0 ZMC81989
26.8 ZMC111795
29.3 ZMC25419
30.5 ZMC83091
30.9 ZMC147853
31.0 ZMC99714
31.6 ZMC55812
31.7 ZMC152411
10 [2]
32.3 ZMC134525
32.7 ZMC124439
32.8 ZMC126457 ZMC89603 ZMC129149
33.0 ZMC73629
33.2 ZMC58852
33.7 ZMC126995 ZMC32192 ZMC99384
33.8 ZMC62977 ZMC117248 ZMC148158
34.0 ZMC108171
34.1 ZMC55188 ZMC157392
34.2 ZMC45681
34.5 ZMC73618
34.6 ZMC87391
35.0 ZMC149802
36.8 ZMC1057
37.3 ZMC85920
39.2 ZMC102271
39.8 ZMC80527
40.2 ZMC44311
40.8 ZMC53480
41.4 ZMC157712
42.1 ZMC122528
42.6 ZMC71056
42.7 ZMC123653
44.2 ZMC128011
44.7 ZMC12475 ZMC36430
45.1 ZMC140619
45.5 ZMC117288
45.6 ZMC85321
46.5 ZMC107349
47.5 ZMC84849
48.1 ZMC42908
48.3 ZMC129915
49.1 ZMC75579
49.8 ZMC86922
50.8 ZMC123103
52.2 ZMC136907
54.1 ZMC43501
54.5 ZMC108447
56.6 ZMC140360
58.0 ZMC26880
59.5 ZMC21169
62.4 ZMC10163

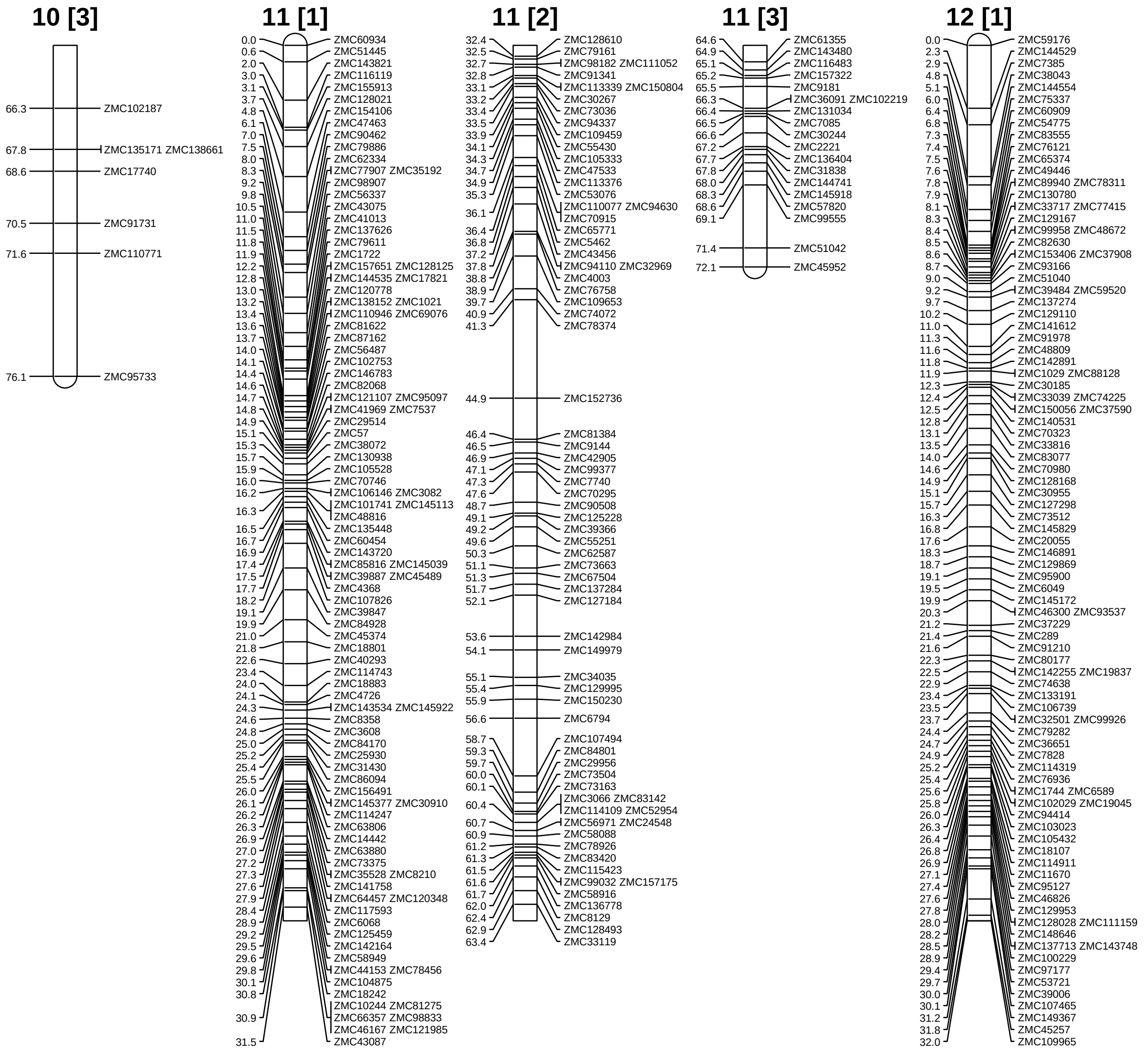
10 [3]
66.3 ZMC102187
67.8 ZMC135171 ZMC138661
68.6 ZMC17740
70.5 ZMC91731
71.6 ZMC110771
76.1 ZMC95733
11 [1]
0.0 ZMC60934
0.6 ZMC51445
2.0 ZMC143821
3.0 ZMC116119
3.1 ZMC155913
3.7 ZMC128021
4.8 ZMC154106
6.1 ZMC47463
7.0 ZMC90462
7.5 ZMC79886
8.0 ZMC62334
8.3 ZMC77907 ZMC35192
9.2 ZMC98907
9.8 ZMC56337
10.5 ZMC43075
11.0 ZMC41013
11.5 ZMC137626
11.8 ZMC79611
11.9 ZMC1722
12.2 ZMC157651 ZMC128125
12.8 ZMC144535 ZMC17821
13.0 ZMC120778
13.2 ZMC138152 ZMC1021
13.4 ZMC110946 ZMC69076
13.6 ZMC81622
13.7 ZMC87162
14.0 ZMC56487
14.1 ZMC102753
14.4 ZMC146783
14.6 ZMC82068
14.7 ZMC121107 ZMC95097
14.8 ZMC41969 ZMC7537
14.9 ZMC29514
15.1 ZMC57
15.3 ZMC38072
15.7 ZMC130938
15.9 ZMC105528
16.0 ZMC70746
16.2 ZMC106146 ZMC3082
16.3 ZMC101741 ZMC145113 ZMC48816
16.5 ZMC135448
16.7 ZMC60454
16.9 ZMC143720
17.4 ZMC85816 ZMC145039
17.5 ZMC39887 ZMC45489
17.7 ZMC4368
18.2 ZMC107826
19.1 ZMC39847
19.9 ZMC84928
21.0 ZMC45374
21.8 ZMC18801
22.6 ZMC40293
23.4 ZMC114743
24.0 ZMC18883
24.1 ZMC4726
24.3 ZMC143534 ZMC145922
24.6 ZMC8358
24.8 ZMC3608
25.0 ZMC84170
25.2 ZMC25930
25.4 ZMC31430
25.5 ZMC86094
26.0 ZMC156491
26.1 ZMC145377 ZMC30910
26.2 ZMC114247
26.3 ZMC63806
26.9 ZMC14442
27.0 ZMC63880
27.2 ZMC73375
27.3 ZMC35528 ZMC8210
27.6 ZMC141758
27.9 ZMC64457 ZMC120348
28.4 ZMC117593
28.9 ZMC6068
29.2 ZMC125459
29.5 ZMC142164
29.6 ZMC58949
29.8 ZMC44153 ZMC78456
30.1 ZMC104875
30.8 ZMC18242
30.9 ZMC10244 ZMC81275 ZMC66357 ZMC98833 ZMC46167 ZMC121985
31.5 ZMC43087
11 [2]
32.4 ZMC128610
32.5 ZMC79161
32.7 ZMC98182 ZMC111052
32.8 ZMC91341
33.1 ZMC113339 ZMC150804
33.2 ZMC30267
33.4 ZMC73036
33.5 ZMC94337
33.9 ZMC109459
34.1 ZMC55430
34.3 ZMC105333
34.7 ZMC47533
34.9 ZMC113376
35.3 ZMC53076
36.1 ZMC110077 ZMC94630
36.4 ZMC70915
36.8 ZMC65771
37.2 ZMC5462
37.8 ZMC43456
38.8 ZMC94110 ZMC32969
38.9 ZMC4003
39.7 ZMC76758
40.9 ZMC109653
41.3 ZMC74072
44.9 ZMC78374
46.4 ZMC152736
46.5 ZMC81384
46.9 ZMC9144
47.1 ZMC42905
47.3 ZMC99377
47.6 ZMC7740
48.7 ZMC70295
49.1 ZMC90508
49.2 ZMC125228
49.6 ZMC39366
50.3 ZMC55251
51.1 ZMC62587
51.3 ZMC73663
51.7 ZMC67504
52.1 ZMC137284
53.6 ZMC127184
54.1 ZMC142984
55.1 ZMC149979
55.4 ZMC34035
55.9 ZMC129995
56.6 ZMC150230
58.7 ZMC6794
59.3 ZMC107494
59.7 ZMC84801
60.0 ZMC29956
60.1 ZMC73504
60.4 ZMC73163
60.7 ZMC3066 ZMC83142
60.9 ZMC114109 ZMC52954
61.2 ZMC56971 ZMC24548
61.3 ZMC58088
61.5 ZMC78926
61.6 ZMC83420
61.7 ZMC115423
62.0 ZMC99032 ZMC157175
62.4 ZMC58916
62.9 ZMC136778
63.4 ZMC8129
ZMC128493
ZMC33119
11 [3]
64.6 ZMC61355
64.9 ZMC143480
65.1 ZMC116483
65.2 ZMC157322
65.5 ZMC9181
66.3 ZMC36091 ZMC102219
66.4 ZMC131034
66.5 ZMC7085
66.6 ZMC30244
67.2 ZMC2221
67.7 ZMC136404
67.8 ZMC31838
68.0 ZMC144741
68.3 ZMC145918
68.6 ZMC57820
69.1 ZMC99555
71.4 ZMC51042
72.1 ZMC45952
12 [1]
0.0 ZMC59176
2.3 ZMC144529
2.9 ZMC7385
4.8 ZMC38043
5.1 ZMC144554
6.0 ZMC75337
6.4 ZMC60909
6.8 ZMC54775
7.3 ZMC83555
7.4 ZMC76121
7.5 ZMC65374
7.6 ZMC49446
7.8 ZMC89940 ZMC78311
7.9 ZMC130780
8.1 ZMC33717 ZMC77415
8.3 ZMC129167
8.4 ZMC99958 ZMC48672
8.5 ZMC82630
8.6 ZMC153406 ZMC37908
8.7 ZMC93166
9.0 ZMC51040
9.2 ZMC39484 ZMC59520
9.7 ZMC137274
10.2 ZMC129110
11.0 ZMC141612
11.3 ZMC91978
11.6 ZMC48809
11.8 ZMC142891
11.9 ZMC1029 ZMC88128
12.3 ZMC30185
12.4 ZMC33039 ZMC74225
12.5 ZMC150056 ZMC37590
12.8 ZMC140531
13.1 ZMC70323
13.5 ZMC33816
14.0 ZMC83077
14.6 ZMC70980
14.9 ZMC128168
15.1 ZMC30955
15.7 ZMC127298
16.3 ZMC73512
16.8 ZMC145829
17.6 ZMC20055
18.3 ZMC146891
18.7 ZMC129869
19.1 ZMC95900
19.5 ZMC6049
19.9 ZMC145172
20.3 ZMC46300 ZMC93537
21.2 ZMC37229
21.4 ZMC289
21.6 ZMC91210
22.3 ZMC80177
22.5 ZMC142255 ZMC19837
22.9 ZMC74638
23.4 ZMC133191
23.5 ZMC106739
23.7 ZMC32501 ZMC99926
24.4 ZMC79282
24.7 ZMC36651
24.9 ZMC7828
25.2 ZMC114319
25.4 ZMC76936
25.6 ZMC1744 ZMC6589
25.8 ZMC102029 ZMC19045
26.0 ZMC94414
26.3 ZMC103023
26.4 ZMC105432
26.8 ZMC18107
26.9 ZMC114911
27.1 ZMC11670
27.4 ZMC95127
27.6 ZMC46826
27.8 ZMC129953
28.0 ZMC128028 ZMC111159
28.2 ZMC148646
28.5 ZMC137713 ZMC143748
28.9 ZMC100229
29.4 ZMC97177
29.7 ZMC53721
30.0 ZMC39006
30.1 ZMC107465
31.2 ZMC149367
31.8 ZMC45257
32.0 ZMC109965

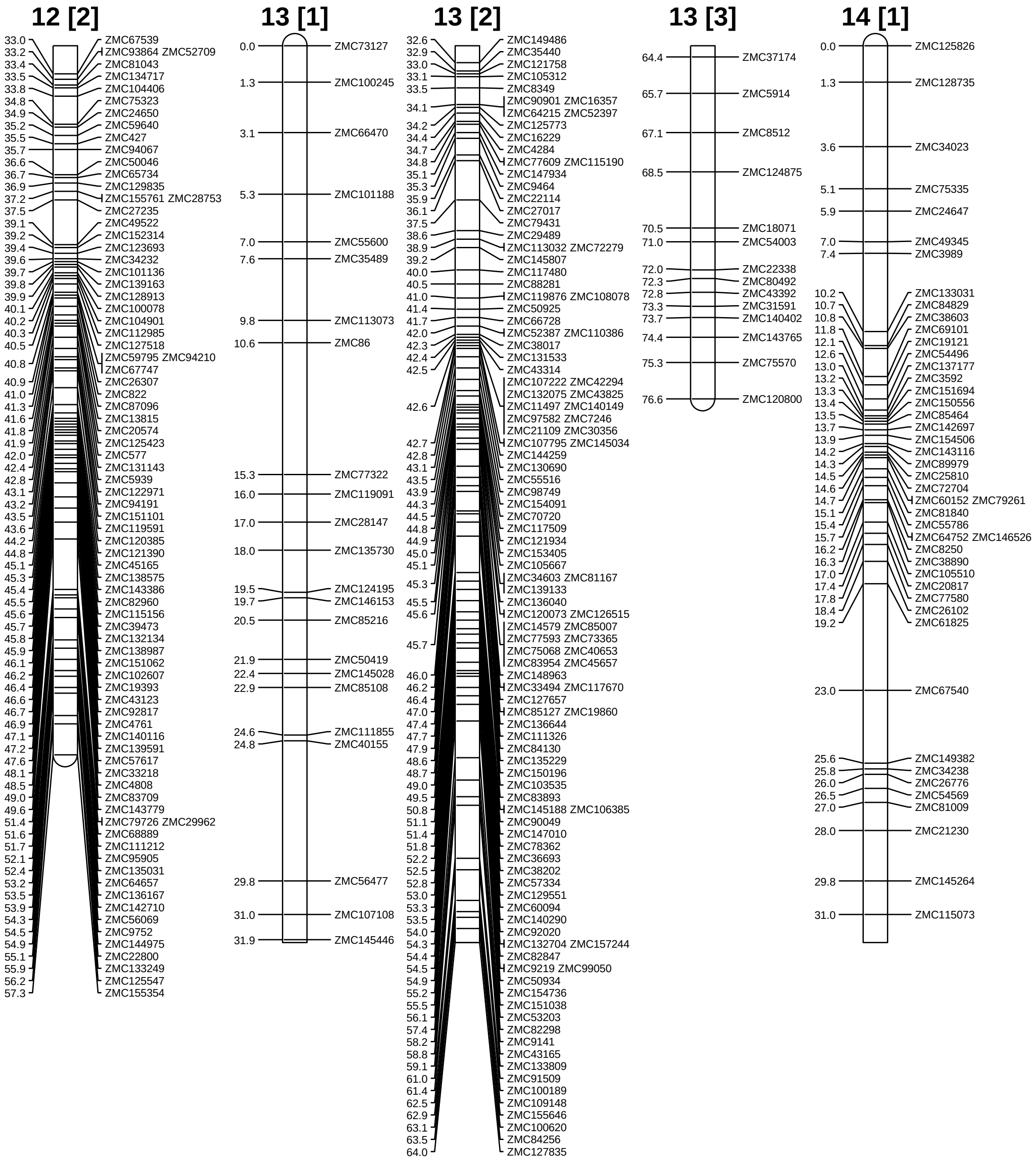
12 [2]
33.0
33.2
33.4
33.5
33.8
34.8
34.9
35.2
35.5
35.7
36.6
36.7
36.9
37.2
37.5
39.1
39.2
39.4
39.6
39.7
39.8
39.9
40.1
40.2
40.3
40.5
40.8
40.9
41.0
41.3
41.6
41.8
41.9
42.0
42.4
42.8
43.1
43.2
43.5
43.6
44.2
44.8
45.1
45.3
45.4
45.5
45.6
45.7
45.8
45.9
46.1
46.2
46.4
46.6
46.7
46.9
47.1
47.2
47.6
48.1
48.5
49.0
49.6
51.4
51.6
51.7
52.1
52.4
53.2
53.5
53.9
54.3
54.5
54.9
55.1
55.9
56.2
57.3
ZMC67539
ZMC93864 ZMC52709
ZMC81043
ZMC134717
ZMC104406
ZMC75323
ZMC24650
ZMC59640
ZMC427
ZMC94067
ZMC50046
ZMC65734
ZMC129835
ZMC155761 ZMC28753
ZMC27235
ZMC49522
ZMC152314
ZMC123693
ZMC34232
ZMC101136
ZMC139163
ZMC128913
ZMC100078
ZMC104901
ZMC112985
ZMC127518
ZMC59795 ZMC94210
ZMC67747
ZMC26307
ZMC822
ZMC87096
ZMC13815
ZMC20574
ZMC125423
ZMC577
ZMC131143
ZMC5939
ZMC122971
ZMC94191
ZMC151101
ZMC119591
ZMC120385
ZMC121390
ZMC45165
ZMC138575
ZMC143386
ZMC82960
ZMC115156
ZMC39473
ZMC132134
ZMC138987
ZMC151062
ZMC102607
ZMC19393
ZMC43123
ZMC92817
ZMC4761
ZMC140116
ZMC139591
ZMC57617
ZMC33218
ZMC4808
ZMC83709
ZMC143779
ZMC79726 ZMC29962
ZMC68889
ZMC111212
ZMC95905
ZMC135031
ZMC64657
ZMC136167
ZMC142710
ZMC56069
ZMC9752
ZMC144975
ZMC22800
ZMC133249
ZMC125547
ZMC155354
13 [1]
0.0 ZMC73127
1.3 ZMC100245
3.1 ZMC66470
5.3 ZMC101188
7.0 ZMC55600
7.6 ZMC35489
9.8 ZMC113073
10.6 ZMC86
15.3 ZMC77322
16.0 ZMC119091
17.0 ZMC28147
18.0 ZMC135730
19.5 ZMC124195
19.7 ZMC146153
20.5 ZMC85216
21.9 ZMC50419
22.4 ZMC145028
22.9 ZMC85108
24.6 ZMC111855
24.8 ZMC40155
29.8 ZMC56477
31.0 ZMC107108
31.9 ZMC145446
13 [2]
32.6
32.9
33.0
33.1
33.5
34.1
34.2
34.4
34.7
34.8
35.1
35.3
35.9
36.1
37.5
38.6
38.9
39.2
40.0
40.5
41.0
41.4
41.7
42.0
42.3
42.4
42.5
42.6
42.7
42.8
43.1
43.5
43.9
44.3
44.5
44.8
44.9
45.0
45.1
45.3
45.5
45.6
45.7
46.0
46.2
46.4
47.0
47.4
47.7
47.9
48.6
48.7
49.0
49.5
50.8
51.1
51.4
51.8
52.2
52.5
52.8
53.0
53.3
53.5
54.0
54.3
54.4
54.5
54.9
55.2
55.5
56.1
57.4
58.2
58.8
59.1
61.0
61.4
62.5
62.9
63.1
63.5
64.0
ZMC149486
ZMC35440
ZMC121758
ZMC105312
ZMC8349
ZMC90901 ZMC16357
ZMC64215 ZMC52397
ZMC125773
ZMC16229
ZMC4284
ZMC77609 ZMC115190
ZMC147934
ZMC9464
ZMC22114
ZMC27017
ZMC79431
ZMC29489
ZMC113032 ZMC72279
ZMC145807
ZMC117480
ZMC88281
ZMC119876 ZMC108078
ZMC50925
ZMC66728
ZMC52387 ZMC110386
ZMC38017
ZMC131533
ZMC43314
ZMC107222 ZMC42294
ZMC132075 ZMC43825
ZMC11497 ZMC140149
ZMC97582 ZMC7246
ZMC21109 ZMC30356
ZMC107795 ZMC145034
ZMC144259
ZMC130690
ZMC55516
ZMC98749
ZMC154091
ZMC70720
ZMC117509
ZMC121934
ZMC153405
ZMC105667
ZMC34603 ZMC81167
ZMC139133
ZMC136040
ZMC120073 ZMC126515
ZMC14579 ZMC85007
ZMC77593 ZMC73365
ZMC75068 ZMC40653
ZMC83954 ZMC45657
ZMC148963
ZMC33494 ZMC117670
ZMC127657
ZMC85127 ZMC19860
ZMC136644
ZMC111326
ZMC84130
ZMC135229
ZMC150196
ZMC103535
ZMC83893
ZMC145188 ZMC106385
ZMC90049
ZMC147010
ZMC78362
ZMC36693
ZMC38202
ZMC57334
ZMC129551
ZMC60094
ZMC140290
ZMC92020
ZMC132704 ZMC157244
ZMC82847
ZMC9219 ZMC99050
ZMC50934
ZMC154736
ZMC151038
ZMC53203
ZMC82298
ZMC9141
ZMC43165
ZMC133809
ZMC91509
ZMC100189
ZMC109148
ZMC155646
ZMC100620
ZMC84256
ZMC127835
13 [3]
64.4 ZMC37174
65.7 ZMC5914
67.1 ZMC8512
68.5 ZMC124875
70.5 ZMC18071
71.0 ZMC54003
72.0 ZMC22338
72.3 ZMC80492
72.8 ZMC43392
73.3 ZMC31591
73.7 ZMC140402
74.4 ZMC143765
75.3 ZMC75570
76.6 ZMC120800
14 [1]
0.0 ZMC125826
1.3 ZMC128735
3.6 ZMC34023
5.1 ZMC75335
5.9 ZMC24647
7.0 ZMC49345
7.4 ZMC3989
10.2 ZMC133031
10.7 ZMC84829
10.8 ZMC38603
11.8 ZMC69101
12.1 ZMC19121
12.6 ZMC54496
13.0 ZMC137177
13.2 ZMC3592
13.3 ZMC151694
13.4 ZMC150556
13.5 ZMC85464
13.7 ZMC142697
13.9 ZMC154506
14.2 ZMC143116
14.3 ZMC89979
14.5 ZMC25810
14.6 ZMC72704
14.7 ZMC60152 ZMC79261
15.1 ZMC81840
15.4 ZMC55786
15.7 ZMC64752 ZMC146526
16.2 ZMC8250
16.3 ZMC38890
17.0 ZMC105510
17.4 ZMC20817
17.8 ZMC77580
18.4 ZMC26102
19.2 ZMC61825
23.0 ZMC67540
25.6 ZMC149382
25.8 ZMC34238
26.0 ZMC26776
26.5 ZMC54569
27.0 ZMC81009
28.0 ZMC21230
29.8 ZMC145264
31.0 ZMC115073

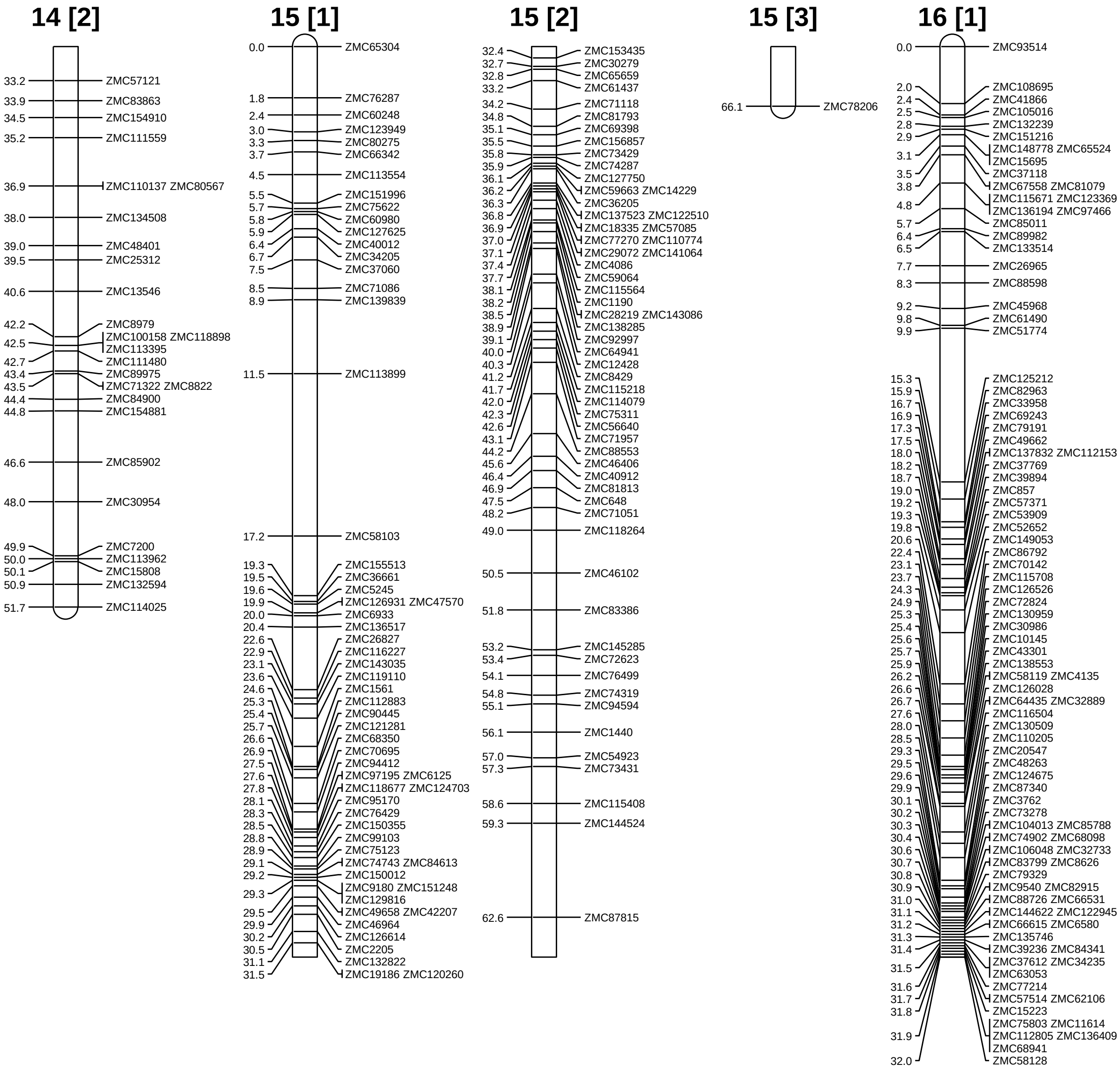
14 [2]
33.2 ZMC57121
33.9 ZMC83863
34.5 ZMC154910
35.2 ZMC111559
36.9 ZMC110137 ZMC80567
38.0 ZMC134508
39.0 ZMC48401
39.5 ZMC25312
40.6 ZMC13546
42.2 ZMC8979
42.5 ZMC100158 ZMC118898
ZMC113395
42.7 ZMC111480
43.4 ZMC89975
43.5 ZMC71322 ZMC8822
44.4 ZMC84900
44.8 ZMC154881
46.6 ZMC85902
48.0 ZMC30954
49.9 ZMC7200
50.0 ZMC113962
50.1 ZMC15808
50.9 ZMC132594
51.7 ZMC114025
15 [1]
0.0 ZMC65304
1.8 ZMC76287
2.4 ZMC60248
3.0 ZMC123949
3.3 ZMC80275
3.7 ZMC66342
4.5 ZMC113554
5.5 ZMC151996
5.7 ZMC75622
5.8 ZMC60980
5.9 ZMC127625
6.4 ZMC40012
6.7 ZMC34205
7.5 ZMC37060
8.5 ZMC71086
8.9 ZMC139839
11.5 ZMC113899
17.2 ZMC58103
19.3 ZMC155513
19.5 ZMC36661
19.6 ZMC5245
19.9 ZMC126931 ZMC47570
20.0 ZMC6933
20.4 ZMC136517
22.6 ZMC26827
22.9 ZMC116227
23.1 ZMC143035
23.6 ZMC119110
24.6 ZMC1561
25.3 ZMC112883
25.4 ZMC90445
25.7 ZMC121281
26.6 ZMC68350
26.9 ZMC70695
27.5 ZMC94412
27.6 ZMC97195 ZMC6125
27.8 ZMC118677 ZMC124703
28.1 ZMC95170
28.3 ZMC76429
28.5 ZMC150355
28.8 ZMC99103
28.9 ZMC75123
29.1 ZMC74743 ZMC84613
29.2 ZMC150012
ZMC9180 ZMC151248
29.3 ZMC129816
29.5 ZMC49658 ZMC42207
29.9 ZMC46964
30.2 ZMC126614
30.5 ZMC2205
31.1 ZMC132822
31.5 ZMC19186 ZMC120260
15 [2]
32.4 ZMC153435
32.7 ZMC30279
32.8 ZMC65659
33.2 ZMC61437
34.2 ZMC71118
34.8 ZMC81793
35.1 ZMC69398
35.5 ZMC156857
35.8 ZMC73429
35.9 ZMC74287
36.1 ZMC127750
36.2 ZMC59663 ZMC14229
36.3 ZMC36205
36.8 ZMC137523 ZMC122510
36.9 ZMC18335 ZMC57085
37.0 ZMC77270 ZMC110774
37.1 ZMC29072 ZMC141064
37.4 ZMC4086
37.7 ZMC59064
38.1 ZMC115564
38.2 ZMC1190
38.5 ZMC28219 ZMC143086
38.9 ZMC138285
39.1 ZMC92997
40.0 ZMC64941
40.3 ZMC12428
41.2 ZMC8429
41.7 ZMC115218
42.0 ZMC114079
42.3 ZMC75311
42.6 ZMC56640
43.1 ZMC71957
44.2 ZMC88553
45.6 ZMC46406
46.4 ZMC40912
46.9 ZMC81813
47.5 ZMC648
48.2 ZMC71051
49.0 ZMC118264
50.5 ZMC46102
51.8 ZMC83386
53.2 ZMC145285
53.4 ZMC72623
54.1 ZMC76499
54.8 ZMC74319
55.1 ZMC94594
56.1 ZMC1440
57.0 ZMC54923
57.3 ZMC73431
58.6 ZMC115408
59.3 ZMC144524
62.6 ZMC87815
15 [3]
66.1 ZMC78206
16 [1]
0.0 ZMC93514
2.0 ZMC108695
2.4 ZMC41866
2.5 ZMC105016
2.8 ZMC132239
2.9 ZMC151216
ZMC148778 ZMC65524
3.1 ZMC15695
3.5 ZMC37118
3.8 ZMC67558 ZMC81079
ZMC115671 ZMC123369
4.8 ZMC136194 ZMC97466
5.7 ZMC85011
6.4 ZMC89982
6.5 ZMC133514
7.7 ZMC26965
8.3 ZMC88598
9.2 ZMC45968
9.8 ZMC61490
9.9 ZMC51774
15.3 ZMC125212
15.9 ZMC82963
16.7 ZMC33958
16.9 ZMC69243
17.3 ZMC79191
17.5 ZMC49662
18.0 ZMC137832 ZMC112153
18.2 ZMC37769
18.7 ZMC39894
19.0 ZMC857
19.2 ZMC57371
19.3 ZMC53909
19.8 ZMC52652
20.6 ZMC149053
22.4 ZMC86792
23.1 ZMC70142
23.7 ZMC115708
24.3 ZMC126526
24.9 ZMC72824
25.3 ZMC130959
25.4 ZMC30986
25.6 ZMC10145
25.7 ZMC43301
25.9 ZMC138553
26.2 ZMC58119 ZMC4135
26.6 ZMC126028
26.7 ZMC64435 ZMC32889
27.6 ZMC116504
28.0 ZMC130509
28.5 ZMC110205
29.3 ZMC20547
29.5 ZMC48263
29.6 ZMC124675
29.9 ZMC87340
30.1 ZMC3762
30.2 ZMC73278
30.3 ZMC104013 ZMC85788
30.4 ZMC74902 ZMC68098
30.6 ZMC106048 ZMC32733
30.7 ZMC83799 ZMC8626
30.8 ZMC79329
30.9 ZMC9540 ZMC82915
31.0 ZMC88726 ZMC66531
31.1 ZMC144622 ZMC122945
31.2 ZMC66615 ZMC6580
31.3 ZMC135746
31.4 ZMC39236 ZMC84341
ZMC37612 ZMC34235
31.5 ZMC63053
31.6 ZMC77214
31.7 ZMC57514 ZMC62106
31.8 ZMC15223
ZMC75803 ZMC11614
31.9 ZMC112805 ZMC136409
ZMC68941
32.0 ZMC58128

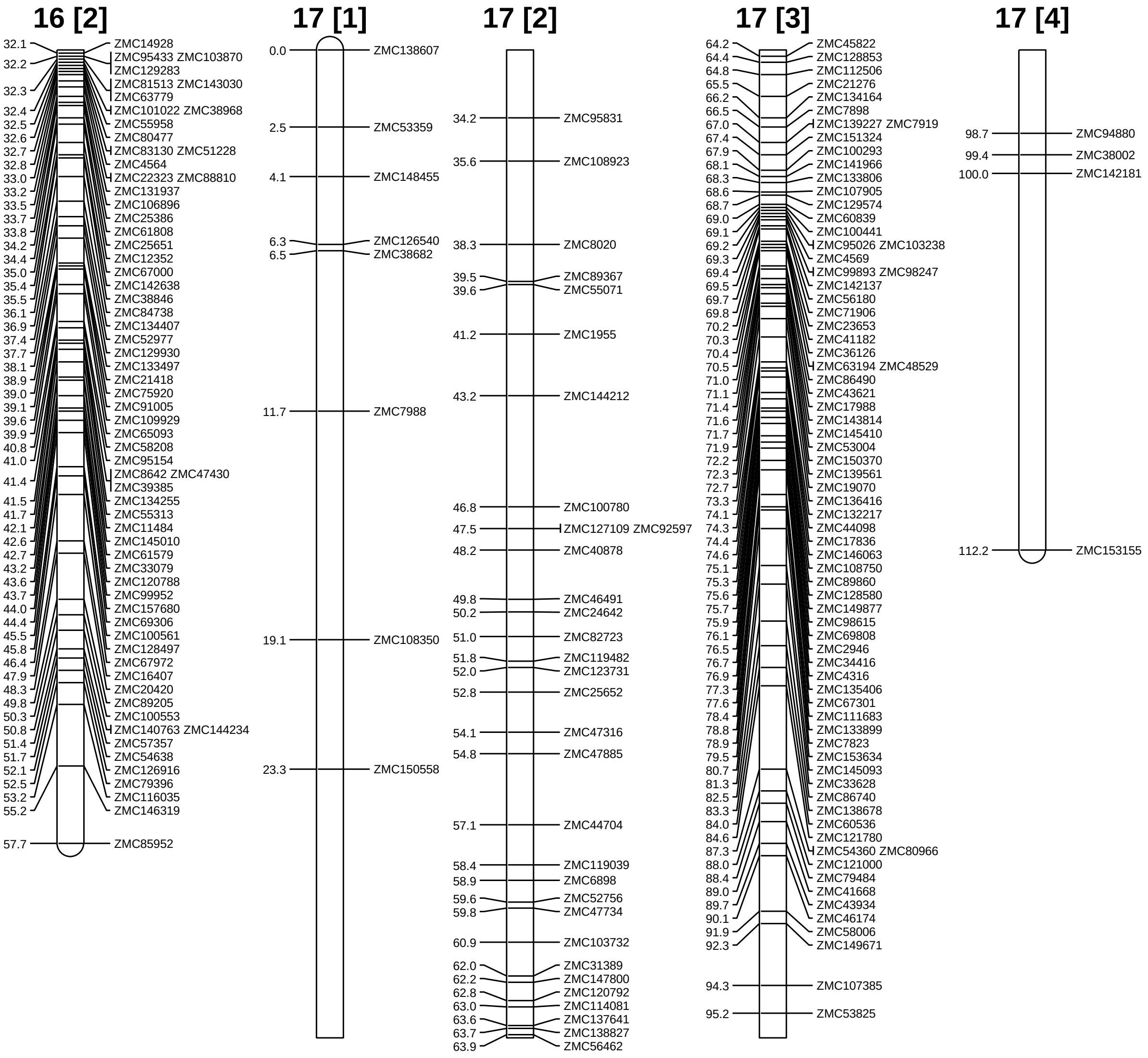
16 [2]
17 [1]
17 [2]
17 [3]
17 [4]

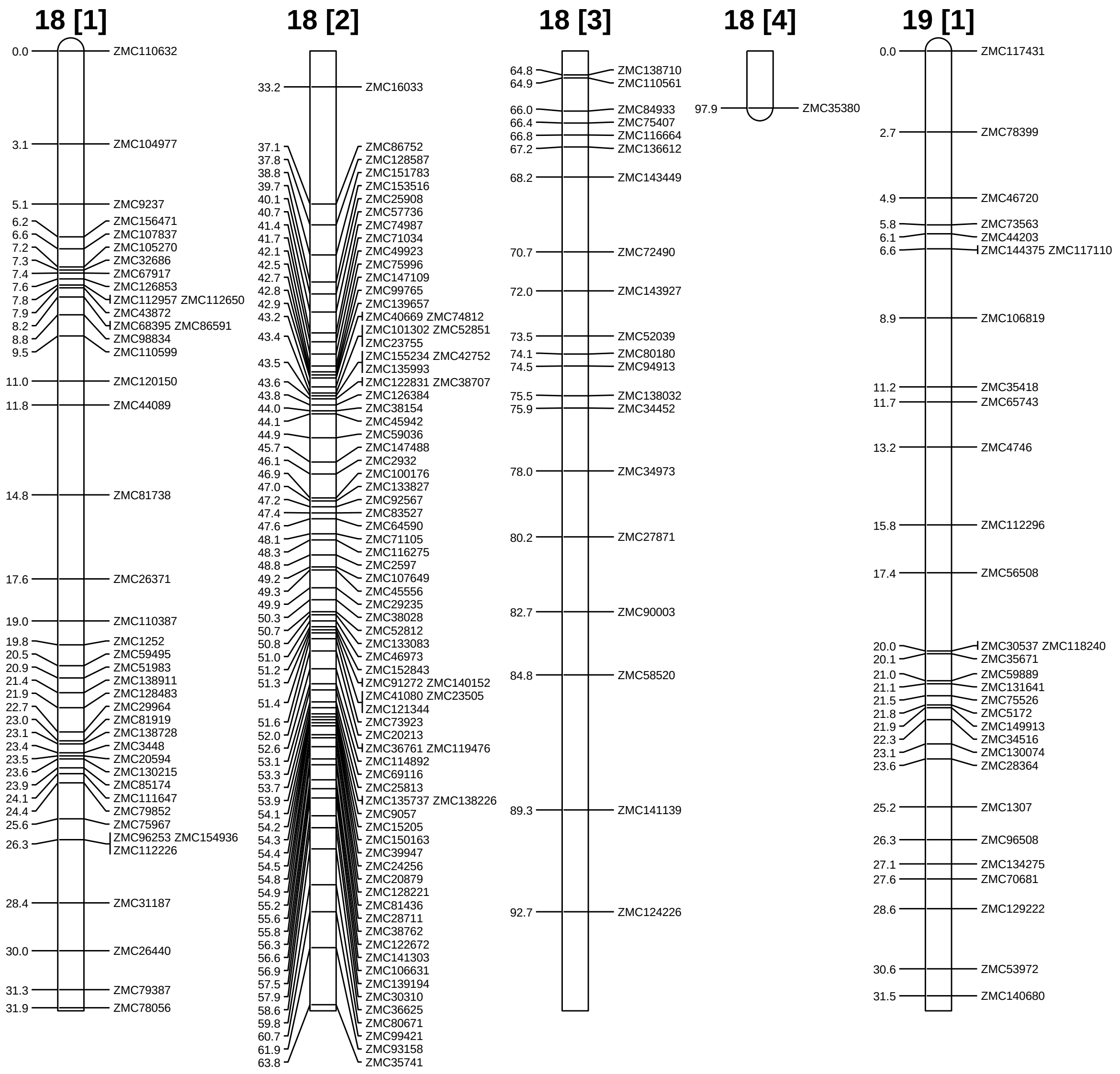
18 [1]
0.0 ZMC110632
3.1 ZMC104977
5.1 ZMC9237
6.2 ZMC156471
6.6 ZMC107837
7.2 ZMC105270
7.3 ZMC32686
7.4 ZMC67917
7.6 ZMC126853
7.8 ZMC112957 ZMC112650
7.9 ZMC43872
8.2 ZMC68395 ZMC86591
8.8 ZMC98834
9.5 ZMC110599
11.0 ZMC120150
11.8 ZMC44089
14.8 ZMC81738
17.6 ZMC26371
19.0 ZMC110387
19.8 ZMC1252
20.5 ZMC59495
20.9 ZMC51983
21.4 ZMC138911
21.9 ZMC128483
22.7 ZMC29964
23.0 ZMC81919
23.1 ZMC138728
23.4 ZMC3448
23.5 ZMC20594
23.6 ZMC130215
23.9 ZMC85174
24.1 ZMC111647
24.4 ZMC79852
25.6 ZMC75967
26.3 ZMC96253 ZMC154936 ZMC112226
28.4 ZMC31187
30.0 ZMC26440
31.3 ZMC79387
31.9 ZMC78056
18 [2]
33.2 ZMC16033
37.1 ZMC86752
37.8 ZMC128587
38.8 ZMC151783
39.7 ZMC153516
40.1 ZMC25908
40.7 ZMC57736
41.4 ZMC74987
41.7 ZMC71034
42.1 ZMC49923
42.5 ZMC75996
42.7 ZMC147109
42.8 ZMC99765
42.9 ZMC139657
43.2 ZMC40669 ZMC74812
43.4 ZMC101302 ZMC52851 ZMC23755
43.5 ZMC155234 ZMC42752 ZMC135993
43.6 ZMC122831 ZMC38707
43.8 ZMC126384
44.0 ZMC38154
44.1 ZMC45942
44.9 ZMC59036
45.7 ZMC147488
46.1 ZMC2932
46.9 ZMC100176
47.0 ZMC133827
47.2 ZMC92567
47.4 ZMC83527
47.6 ZMC64590
48.1 ZMC71105
48.3 ZMC116275
48.8 ZMC2597
49.2 ZMC107649
49.3 ZMC45556
49.9 ZMC29235
50.3 ZMC38028
50.7 ZMC52812
50.8 ZMC133083
51.0 ZMC46973
51.2 ZMC152843
51.3 ZMC91272 ZMC140152
51.4 ZMC41080 ZMC23505 ZMC121344
51.6 ZMC73923
52.0 ZMC20213
52.6 ZMC36761 ZMC119476
53.1 ZMC114892
53.3 ZMC69116
53.7 ZMC25813
53.9 ZMC135737 ZMC138226
54.1 ZMC9057
54.2 ZMC15205
54.3 ZMC150163
54.4 ZMC39947
54.5 ZMC24256
54.8 ZMC20879
54.9 ZMC128221
55.2 ZMC81436
55.6 ZMC28711
55.8 ZMC38762
56.3 ZMC122672
56.6 ZMC141303
56.9 ZMC106631
57.5 ZMC139194
57.9 ZMC30310
58.6 ZMC36625
59.8 ZMC80671
60.7 ZMC99421
61.9 ZMC93158
63.8 ZMC35741
18 [3]
64.8 ZMC138710
64.9 ZMC110561
66.0 ZMC84933
66.4 ZMC75407
66.8 ZMC116664
67.2 ZMC136612
68.2 ZMC143449
70.7 ZMC72490
72.0 ZMC143927
73.5 ZMC52039
74.1 ZMC80180
74.5 ZMC94913
75.5 ZMC138032
75.9 ZMC34452
78.0 ZMC34973
80.2 ZMC27871
82.7 ZMC90003
84.8 ZMC58520
89.3 ZMC141139
92.7 ZMC124226
18 [4]
97.9 ZMC35380
19 [1]
0.0 ZMC117431
2.7 ZMC78399
4.9 ZMC46720
5.8 ZMC73563
6.1 ZMC44203
6.6 ZMC144375 ZMC117110
8.9 ZMC106819
11.2 ZMC35418
11.7 ZMC65743
13.2 ZMC4746
15.8 ZMC112296
17.4 ZMC56508
20.0 ZMC30537 ZMC118240
20.1 ZMC35671
21.0 ZMC59889
21.1 ZMC131641
21.5 ZMC75526
21.8 ZMC5172
21.9 ZMC149913
22.3 ZMC34516
23.1 ZMC130074
23.6 ZMC28364
25.2 ZMC1307
26.3 ZMC96508
27.1 ZMC134275
27.6 ZMC70681
28.6 ZMC129222
30.6 ZMC53972
31.5 ZMC140680

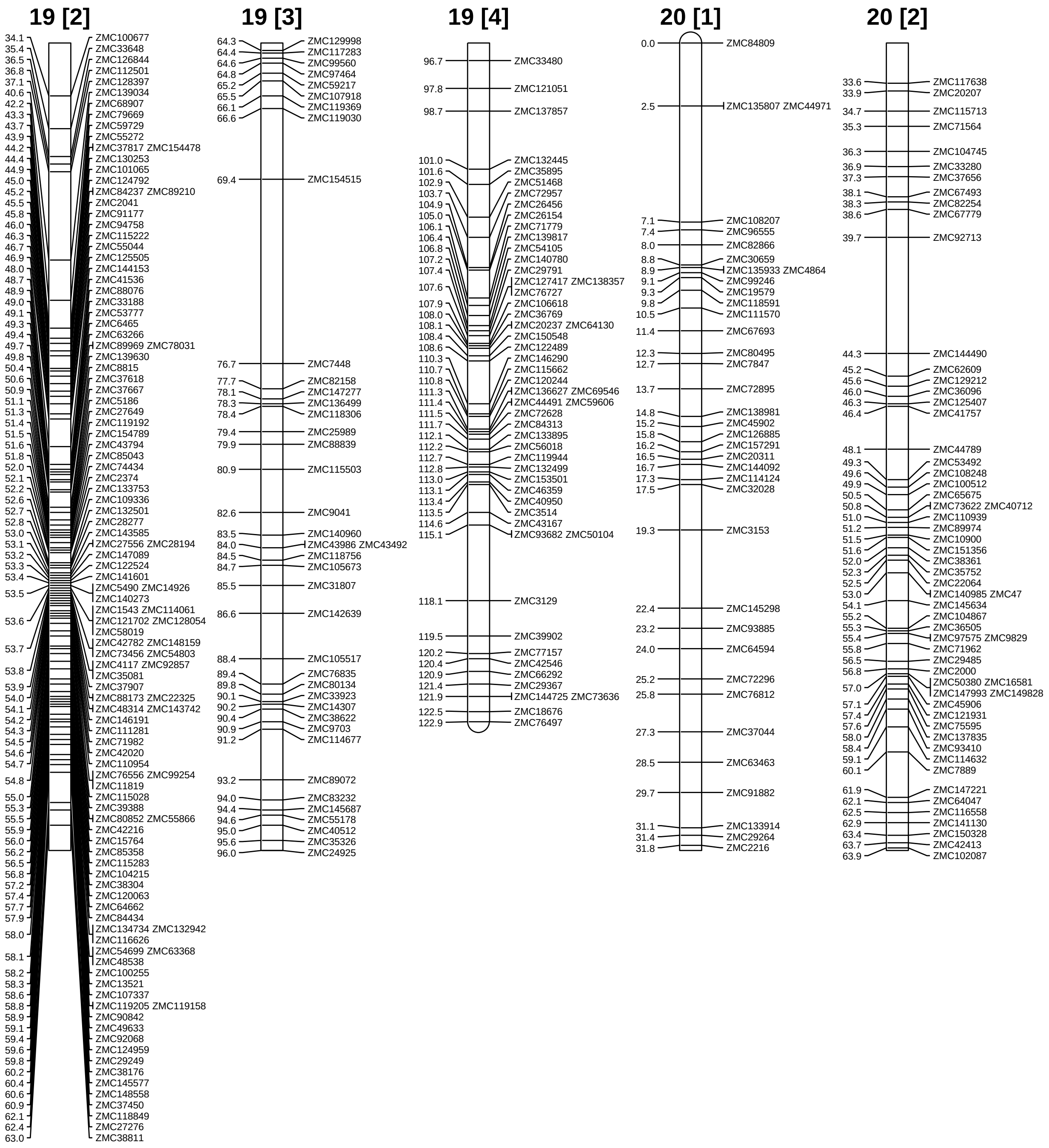
19 [2]
34.1 35.4 36.5 36.8 37.1 40.6 42.2 43.3 43.7 43.9 44.2 44.4 44.9 45.0 45.2 45.5 45.8 46.0 46.3 46.7 46.9 48.0 48.7 48.9 49.0 49.1 49.3 49.4 49.7 49.8 50.4 50.6 50.9 51.1 51.3 51.4 51.5 51.6 51.8 52.0 52.1 52.2 52.6 52.7 52.8 53.0 53.1 53.2 53.3 53.4 53.5 53.6 53.7 53.8 53.9 54.0 54.1 54.2 54.3 54.5 54.6 54.7 54.8 55.0 55.3 55.5 55.9 56.0 56.2 56.5 56.8 57.2 57.4 57.7 57.9 58.0 58.1 58.2 58.3 58.6 58.8 58.9 59.1 59.4 59.6 59.8 60.2 60.4 60.6 60.9 62.1 62.4 63.0
ZMC100677
ZMC33648
ZMC126844
ZMC112501
ZMC128397
ZMC139034
ZMC68907
ZMC79669
ZMC59729
ZMC55272
ZMC37817 ZMC154478
ZMC130253
ZMC101065
ZMC124792
ZMC84237 ZMC89210
ZMC2041
ZMC91177
ZMC94758
ZMC115222
ZMC55044
ZMC125505
ZMC144153
ZMC41536
ZMC88076
ZMC33188
ZMC53777
ZMC6465
ZMC63266
ZMC89969 ZMC78031
ZMC139630
ZMC8815
ZMC37618
ZMC37667
ZMC5186
ZMC27649
ZMC119192
ZMC154789
ZMC43794
ZMC85043
ZMC74434
ZMC2374
ZMC133753
ZMC109336
ZMC132501
ZMC28277
ZMC143585
ZMC27556 ZMC28194
ZMC147089
ZMC122524
ZMC141601
ZMC5490 ZMC14926
ZMC140273
ZMC1543 ZMC114061
ZMC121702 ZMC128054
ZMC58019
ZMC42782 ZMC148159
ZMC73456 ZMC54803
ZMC4117 ZMC92857
ZMC35081
ZMC37907
ZMC88173 ZMC22325
ZMC48314 ZMC143742
ZMC146191
ZMC111281
ZMC71982
ZMC42020
ZMC110954
ZMC76556 ZMC99254
ZMC11819
ZMC115028
ZMC39388
ZMC80852 ZMC55866
ZMC42216
ZMC15764
ZMC85358
ZMC115283
ZMC104215
ZMC38304
ZMC120063
ZMC64662
ZMC84434
ZMC134734 ZMC132942
ZMC116626
ZMC54699 ZMC63368
ZMC48538
ZMC100255
ZMC13521
ZMC107337
ZMC119205 ZMC119158
ZMC90842
ZMC49633
ZMC92068
ZMC124959
ZMC29249
ZMC38176
ZMC145577
ZMC148558
ZMC37450
ZMC118849
ZMC27276
ZMC38811
19 [3]
64.3 ZMC129998
64.4 ZMC117283
64.6 ZMC99560
64.8 ZMC97464
65.2 ZMC59217
65.5 ZMC107918
66.1 ZMC119369
66.6 ZMC119030
69.4 ZMC154515
76.7 ZMC7448
77.7 ZMC82158
78.1 ZMC147277
78.3 ZMC136499
78.4 ZMC118306
79.4 ZMC25989
79.9 ZMC88839
80.9 ZMC115503
82.6 ZMC9041
83.5 ZMC140960
84.0 ZMC43986 ZMC43492
84.5 ZMC118756
84.7 ZMC105673
85.5 ZMC31807
86.6 ZMC142639
88.4 ZMC105517
89.4 ZMC76835
89.8 ZMC80134
90.1 ZMC33923
90.2 ZMC14307
90.4 ZMC38622
90.9 ZMC9703
91.2 ZMC114677
93.2 ZMC89072
94.0 ZMC83232
94.4 ZMC145687
94.6 ZMC55178
95.0 ZMC40512
95.6 ZMC35326
96.0 ZMC24925
19 [4]
96.7 ZMC33480
97.8 ZMC121051
98.7 ZMC137857
101.0 ZMC132445
101.6 ZMC35895
102.9 ZMC51468
103.7 ZMC72957
104.9 ZMC26456
105.0 ZMC26154
106.1 ZMC71779
106.4 ZMC139817
106.8 ZMC54105
107.2 ZMC140780
107.4 ZMC29791
107.6 ZMC127417 ZMC138357 ZMC76727
107.9 ZMC106618
108.0 ZMC36769
108.1 ZMC20237 ZMC64130
108.4 ZMC150548
108.6 ZMC122489
110.3 ZMC146290
110.7 ZMC115662
110.8 ZMC120244
111.3 ZMC136627 ZMC69546
111.4 ZMC44491 ZMC59606
111.5 ZMC72628
111.7 ZMC84313
112.1 ZMC133895
112.2 ZMC56018
112.7 ZMC119944
112.8 ZMC132499
113.0 ZMC153501
113.1 ZMC46359
113.4 ZMC40950
113.5 ZMC3514
114.6 ZMC43167
115.1 ZMC93682 ZMC50104
118.1 ZMC3129
119.5 ZMC39902
120.2 ZMC77157
120.4 ZMC42546
120.9 ZMC66292
121.4 ZMC29367
121.9 ZMC144725 ZMC73636
122.5 ZMC18676
122.9 ZMC76497
20 [1]
0.0 ZMC84809
2.5 ZMC135807 ZMC44971
7.1 ZMC108207
7.4 ZMC96555
8.0 ZMC82866
8.8 ZMC30659
8.9 ZMC135933 ZMC4864
9.1 ZMC99246
9.3 ZMC19579
9.8 ZMC118591
10.5 ZMC111570
11.4 ZMC67693
12.3 ZMC80495
12.7 ZMC7847
13.7 ZMC72895
14.8 ZMC138981
15.2 ZMC45902
15.8 ZMC126885
16.2 ZMC157291
16.5 ZMC20311
16.7 ZMC144092
17.3 ZMC114124
17.5 ZMC32028
19.3 ZMC3153
22.4 ZMC145298
23.2 ZMC93885
24.0 ZMC64594
25.2 ZMC72296
25.8 ZMC76812
27.3 ZMC37044
28.5 ZMC63463
29.7 ZMC91882
31.1 ZMC133914
31.4 ZMC29264
31.8 ZMC2216
20 [2]
33.6 ZMC117638
33.9 ZMC20207
34.7 ZMC115713
35.3 ZMC71564
36.3 ZMC104745
36.9 ZMC33280
37.3 ZMC37656
38.1 ZMC67493
38.3 ZMC82254
38.6 ZMC67779
39.7 ZMC92713
44.3 ZMC144490
45.2 ZMC62609
45.6 ZMC129212
46.0 ZMC36096
46.3 ZMC125407
46.4 ZMC41757
48.1 ZMC44789
49.3 ZMC53492
49.6 ZMC108248
49.9 ZMC100512
50.5 ZMC65675
50.8 ZMC73622 ZMC40712
51.0 ZMC110939
51.2 ZMC89974
51.5 ZMC10900
51.6 ZMC151356
52.0 ZMC38361
52.3 ZMC35752
52.5 ZMC22064
53.0 ZMC140985 ZMC47
54.1 ZMC145634
55.2 ZMC104867
55.3 ZMC36505
55.4 ZMC97575 ZMC9829
55.8 ZMC71962
56.5 ZMC29485
56.8 ZMC2000
57.0 ZMC50380 ZMC16581 ZMC147993 ZMC149828
57.1 ZMC45906
57.4 ZMC121931
57.6 ZMC75595
58.0 ZMC137835
58.4 ZMC93410
59.1 ZMC114632
60.1 ZMC7889
61.9 ZMC147221
62.1 ZMC64047
62.5 ZMC116558
62.9 ZMC141130
63.4 ZMC150328
63.7 ZMC42413
63.9 ZMC102087

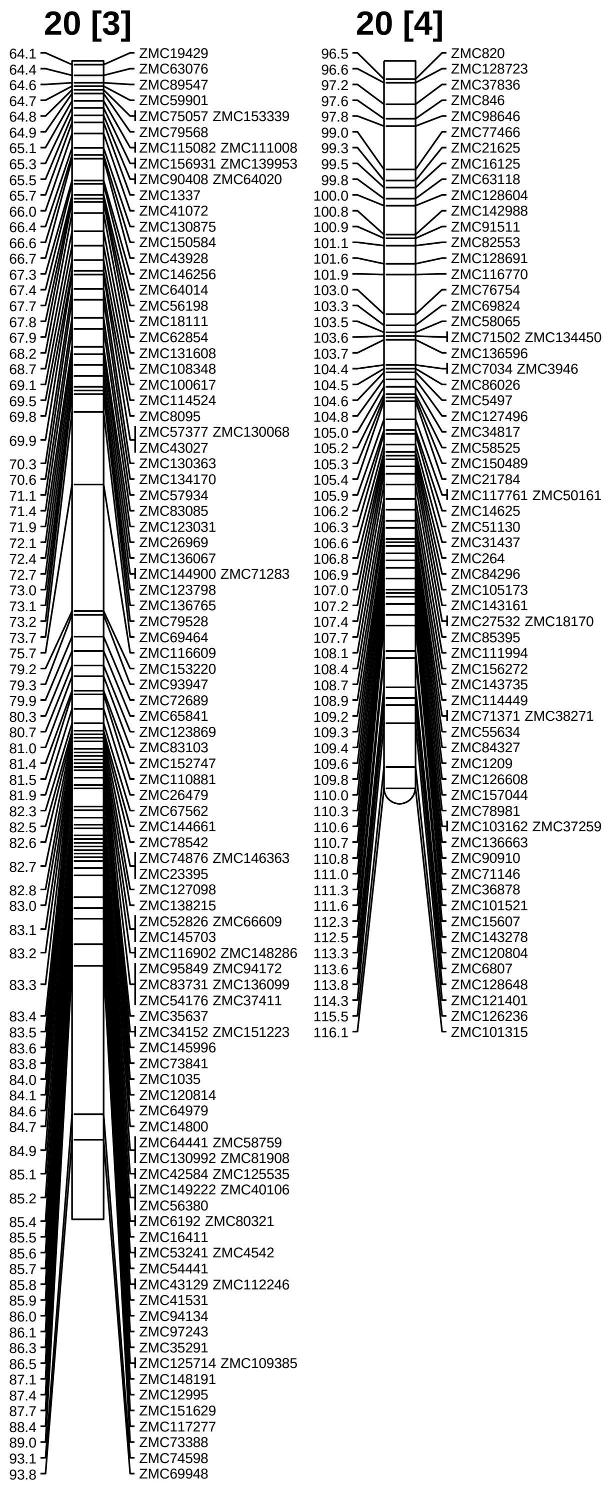

Figure S3 Detailed genetic map of Cavalier

Numbers on top of the maps: linkage group (LG); numbers on the left side of each LG: genetic distance (cM); numbers on the right side of each LG: marker name.
