# Supplementary material for: Construction of high-resolution genetic maps of Zoysia matrella (L.) Merrill and applications to comparative genomic analysis and QTL mapping of resistance to fall armyworm
Source: BMC Genomics. 2016 Aug 8;17:562. doi: 10.1186/s12864-016-2969-7 (PMC4977732; doi:10.1186/s12864-016-2969-7)
Supplement: Additional file 11: Figure S4. — The frequency distribution of the mortality rate of larvae fed leaf tissue from each individual of the mapping population. D: cultivar Diamond (susceptible); C: cultivar Cavalier (resistant). (PDF 311 kb) [file 12864_2016_2969_MOESM11_ESM.pdf]

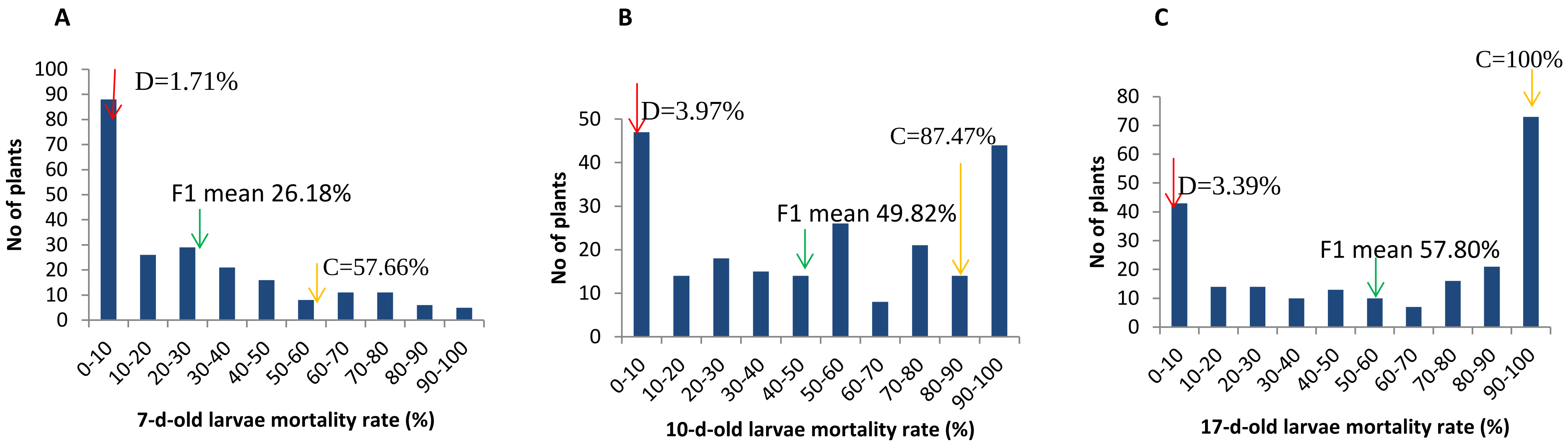

Figure S4 The frequency distribution of the mortality rate of larvae fed leaf tissue from each individual of the mapping population. D: cultivar 'Diamond' (susceptible); C: cultivar 'Cavalier' (resistant).
