# Supplementary material for: Construction of high-resolution genetic maps of Zoysia matrella (L.) Merrill and applications to comparative genomic analysis and QTL mapping of resistance to fall armyworm
Source: BMC Genomics. 2016 Aug 8;17:562. doi: 10.1186/s12864-016-2969-7 (PMC4977732; doi:10.1186/s12864-016-2969-7)
Supplement: Additional file 12: Figure S5. — Cavalier LG8 without distorted marekrs and after adding distorted markers. 8: LG8 without distorted markers; 8’: LG8 after adding distorted markers. (PDF 179 kb) [file 12864_2016_2969_MOESM12_ESM.pdf]

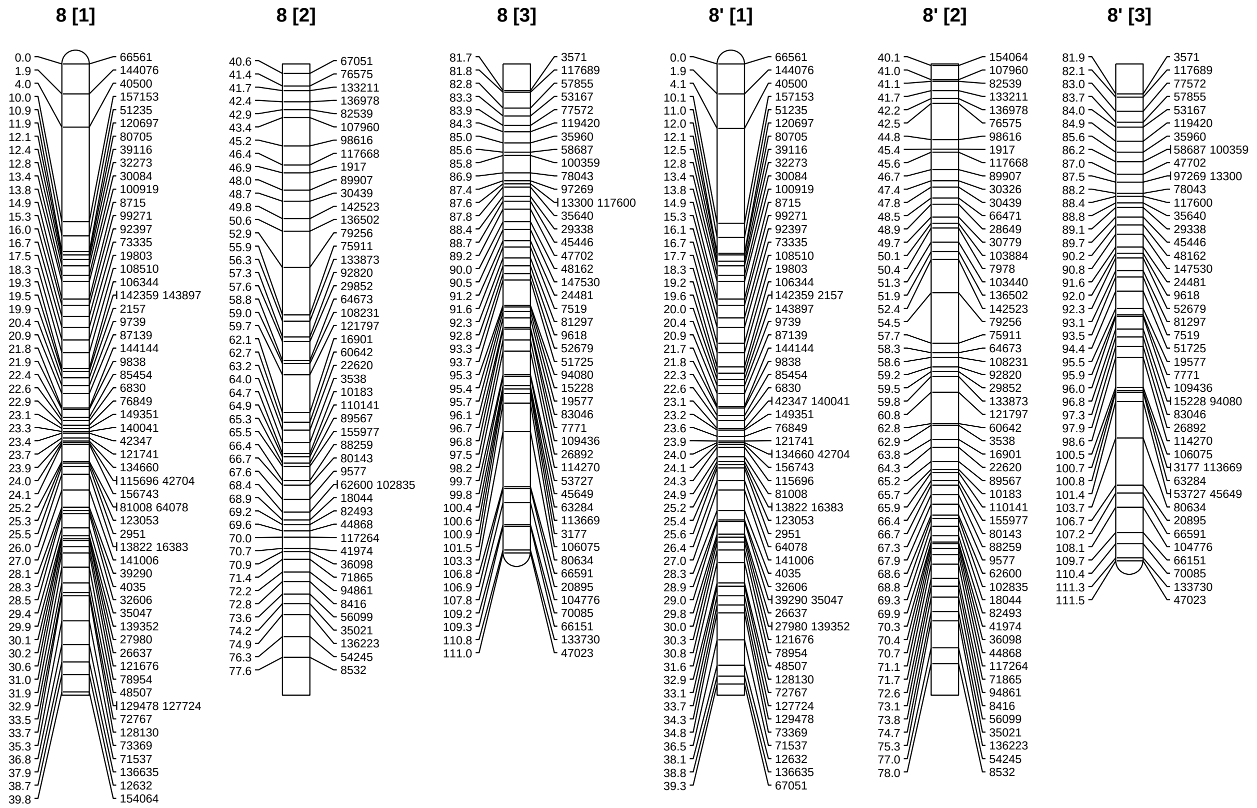

**Figure S5** Cavalier LG8 without distorted marekrs and after adding distorted markers. 8: LG8 without distorted markers; 8': LG8 after adding distorted markers.
